# Comparison of imputation methods for univariate categorical longitudinal data

**DOI:** 10.1007/s11135-024-02028-z

**Published:** 2024-12-26

**Authors:** Kevin Emery, Matthias Studer, André Berchtold

**Affiliations:** 1 Swiss Centre of Expertise in Life Course Research LIVES, Geneva, Switzerland; 2 Institute of Demographics and Socioeconomics, University of Geneva, Geneva, Switzerland; 3 Institute of Social Sciences, University of Lausanne, Lausanne, Switzerland

**Keywords:** Fully conditional specification, Imputation, Life course data, Missing data, Multiple imputation for categorical time series

## Abstract

The life course paradigm emphasizes the need to study not only the situation at a given point in time, but also its evolution over the life course in the medium and long term. These trajectories are often represented by categorical data. This article aims to provide a comprehensive review of the multiple imputation methods proposed so far in the context of univariate categorical data and to assess their practical relevance through a simulation study based on real data. The primary goal is to provide clear methodological guidelines and improve the handling of missing data in life course research. In parallel, we develop the MICT-timing algorithm, which is an extension of the MICT algorithm. This innovative multiple imputation method improves the quality of imputation in trajectories subject to time-varying transition rates, a situation often encountered in life course data.

**Supplementary Information:**

The online version contains supplementary material available at 10.1007/s11135-024-02028-z.

## Introduction

The life course paradigm has become increasingly influential in the social sciences and has made significant contributions to a variety of disciplines, including sociology, demography, gerontology, medicine, and psychology (Elder et al. 2003; Bernardi et al. 2019). This paradigm emphasizes the importance of not only studying an individual’s situation at a given point in time, but also of tracking how it evolves over the medium or long term. Life course trajectories are often described using univariate categorical data. For example, the school-to-work literature focuses on trajectories of occupational integration after compulsory education, distinguishing between education, employment, or unemployment (e.g. Brzinsky-Fay and Solga 2016).

This life course perspective therefore implies the use of longitudinal data over the medium to long term. This data requirement is highly sensitive to missing data because it multiplies the number of missing data occasions and retrospective questions tend to be more difficult to answer. Consequently, the treatment of missing data is one of the main challenges in life course methodology (Piccarreta and Studer 2018).

One should distinguish between two types of conceptually different missing information: data that could have been collected but is missing, and non-existent data (Liao et al. 2022). In the first case, the data is missing, but a meaningful value could exist. This typically occurs due to non-response. In this case, imputation is relevant. In contrast, non-existent data refers to situations where no value is applicable, such as when an individual dies. In this latter case, the use of imputation might be misleading. In this study, we focus on the first case, which we refer to as “missing data”.

Various strategies have been proposed to address missing data, with their relevance contingent on the missing data mechanism. Rubin (1987) delineates three missing data mechanisms. Data are classified as missing completely at random (MCAR) when no systematic difference exists between observed and missing data, although this assumption is generally unrealistic. Conversely, data are considered missing at random (MAR) when observed characteristics account for their missingness. Finally, when the missingness depends on the missing value itself, data are missing not at random (MNAR).

For MCAR, a common strategy is to remove observations with missing values, also known as complete case analysis or available case analysis. While this approach provides unbiased estimates, it can significantly reduce the statistical power of the analysis. Given the frequency of missing data in longitudinal analysis, this approach is often inefficient. In practice, missing data are more commonly MAR, occurring in association with specific profiles. For example, panel attrition, which refers to individuals leaving a longitudinal survey, is associated with vulnerable situations such as unemployment, migration background, or poor health (Rothenbühler and Voorpostel 2016). When dealing with MNAR cases, for instance when respondent “omit” to report unemployment spells in retrospective surveys, standard strategies may introduce bias. Therefore, it is necessary to use a model for the missing data generation process.

We can distinguish three strategies for dealing with missing data: weighting methods, likelihood-based approaches, and imputations, whether simple or multiple (Molenberghs et al. 2014). First, the fully observed trajectories can be weighted to make the sample more representative of the target population, at least in terms of sociodemographic characteristics. These weights are often based on the characteristics used to construct the original sample (Little et al. 2022), such as gender, age, or occupation. Second, likelihood-based approaches rely on a hypothesized distribution of the complete data, often the multivariate normal distribution. Provided the missing data mechanism is either MCAR or MAR, an unbiased estimate of the distribution parameters can be obtained. Finally, imputation methods work by replacing missing values with likely values. The simplest way to do this is to replace each missing value with a single value, resulting in a complete dataset. However, this approach does not take into account the inherent uncertainty of missing values. The multiple imputation framework aims to solve this problem by imputing the missing values *M* times, resulting in *M* completed datasets (Rubin 1987). Statistical analysis is then performed on each dataset separately before the results are aggregated. Multiple imputation is considered a highly efficient and flexible strategy (Molenberghs et al. 2014).

The multiple imputation framework requires an imputation method. The two most common are fully conditional specification (*FCS*) (Van Buuren et al. 2006), also called chained equations (Van Buuren and Groothuis-Oudshoorn 2011), and joint modeling (*JM*) (Schafer 1997). *FCS* uses a separate imputation model for each incomplete variable. The algorithm then iterates over each variable to impute the missing values. The entire imputation process is repeated until convergence is reached. *JM* is based on a multivariate model fitted to the data, usually assuming a normal distribution. The imputations are then randomly drawn from this model.

While *FCS* and *JM* are applicable to longitudinal data by treating repeated measurements of a variable over time as different variables, their standard forms do not directly account for the often strong relationships between successive observations of the same variable. To address this, several algorithm variants have been proposed, including the two-fold *FCS* (Nevalainen et al. 2009). This algorithm relies on the previous, current, and subsequent time points, excluding all other time points. In addition, the algorithm runs several times in a row at the same time point before moving on to the next time point.

Several imputation algorithms have been developed specifically for longitudinal categorical data. Gabadinho and Ritschard (2016) proposed the use of a variable length Markov model (*VLMC*) to impute missing values. Another method, introduced by Halpin (2016) and called “Multiple Imputation for Categorical Time Series” (*MICT*), focuses on imputing successive missing data, thereby forming a gap of missing data, recursively from the edges of this gap.

Previous studies have compared different multiple imputation methods on longitudinal data. Kalaycioglu et al. (2016) conducted an in-depth comparison of Bayesian multiple imputation, multivariate normal joint modeling, and different versions of *FCS* using real and simulated data of a numerical outcome with different types of explanatory variables. De Silva et al. (2017) conducted a simulation study to compare *FCS*, two-fold *FCS*, and *JM* in the presence of a time-varying covariate with a nonlinear association with time. Huque et al. (2018) used 12 algorithms that were different variations of the *FCS* and *JM* imputation algorithms and compared their impact on both a linear regression and a linear mixed effects model. All three studies focused on estimating regression parameters with a numerical outcome. The first study found that *FCS* should be preferred when successive data points are highly correlated, while the other two concluded that *FCS* and *JM* performed well. However, none of these comparisons involved categorical data. As a result, *MICT* and *VLMC*, which focus on longitudinal categorical data, were not evaluated. Furthermore, these studies focused on regression coefficients, whereas other methods, such as classification, are also widely used in life course research.

Longitudinal categorical data used in life course research typically have several characteristics that require a special approach to handling missing data:

- Because of their longitudinal structure, missing values usually appear as gaps, i.e., consecutive missing observations. This may be due, for example, to individuals dropping out of a survey temporarily, or to periods in a retrospective life history calendar that are not filled in.
- Categorical data are not normally distributed, making the application of likelihood-based methods and joint modeling challenging.
- The imputation of categorical data (except for ordinal variables) is generally either true or false, with no gradation between these two extremes.
- Although life course data may involve few transitions, life trajectories are often considered as a whole rather than as a time-ordered sequence of separate events (Piccarreta and Studer 2018).

This study aims to evaluate the strengths and weaknesses of different imputation algorithms in the context of life course research, with the goal of providing recommendations for researchers in the selection and parametrization of appropriate methods. Adopting a typical comparison framework (Oberman and Vink 2023), we use resampled real datasets to assess the impact on bias, coverage, and variance of selected estimands. Tailored to life course research, the framework includes six datasets that highlight common characteristics of univariate longitudinal categorical data. We then simulate missing data in these datasets using three models to replicate common missing data patterns. Finally, we assess the bias, coverage, and variance of estimands focusing on three key aspects of longitudinal categorical data analysis within a life course perspective: timing, duration, and sequencing of processes (Studer and Ritschard 2016).

We also propose two extensions to the MICT algorithm. First, we introduce the MICT-timing algorithm to address a limitation of the MICT algorithm, which assumes that position in the trajectory is irrelevant—an assumption that does not hold in many life course applications. For example, the transition rate between education and work may vary significantly over time, with individuals remaining in education during childhood and transitioning from education to work mostly between the ages of 16 and 30. In such cases, the MICT algorithm may induce too many transitions early in and even impute transitions to work during childhood. We designed the MICT-timing algorithm to overcome this limitation. Second, we explore the use of random forest instead of a multinomial model. Random forest is commonly used for missing data imputation (Burgette and Reiter 2010; Shah et al. 2014; Doove et al. 2014). It can handle nonlinear relationships, interaction effects, and is resistant to irrelevant predictors (Friedman et al. 2001). All of these features can be critical for longitudinal data, where combinations of states can trigger long-term effects. Furthermore, random forest is not too sensitive to the choice of its settings and performs well with a default parameterization (Cutler et al. 2012).

The remainder of this article is organized as follows: First, we introduce the imputation methods under comparison. Next, we present the simulation framework, including the datasets used, the missing data generation processes, and the evaluation criteria. Finally, we examine the results obtained and conclude with a discussion of the findings and general recommendations.

## Imputation methods

This section introduces the algorithms used in the simulations, along with proposed extensions. The algorithms considered are *FCS*, *MICT*, *MICT-timing*, and *VLMC*. We also discuss their parametrizations, aiming to ensure comparability of results by including similar parametrizations across imputation algorithms where possible.

### Fully conditional specification (FCS)

*Fully conditional specification* (*FCS*) is an iterative algorithm that imputes missing data for each variable by cycling sequentially through all variables multiple times, starting from an initial imputation (Van Buuren et al. 2006). It works like this for categorical data:

1. An initial imputation is performed for each missing value, using for each variable the marginal distribution.
2. A separate imputation model is defined for each variable, using multinomial regression or random forest for categorical data. All other variables are typically used as predictors in the imputation model.
3. The algorithm then iterates over the variables to impute again the values that originally had missing data. For each variable, it fits an imputation model using all observations except those that originally had missing values on the variable of interest. In the case of a multinomial model, the method of White et al. (2010) is applied to avoid the problem of perfect predictions (see section 3.6.2 of Van Buuren (2018)). Missing values are imputed based on a random draw according to the probabilities predicted by the models.
4. The previous operation is repeated until a predefined number of iterations is reached.
5. The values obtained in the last iteration are kept.
6. The process is repeated if multiple imputations are required.

*FCS* can be adapted for use with longitudinal data by treating repeated measurements as separate variables. However, collinearity problems may arise due to high associations between successive measurements (Kalaycioglu et al. 2016). *Two-fold FCS* attempts to mitigate this problem by restricting predictors to those observed at the same time points and to a limited number of previous and future measurements of the variable to be imputed. Furthermore, in situations where several variables are measured simultaneously, the algorithm iterates through the variables at each time point multiple times before moving on to the next. However, since our study focuses on univariate longitudinal data, this aspect is not applicable. Therefore, both approaches will be referred to as *FCS* throughout the rest of this article.

For univariate categorical longitudinal data, there are two critical considerations for *FCS*: the choice between a multinomial model or a random forest for the imputation model, and the choice of predictors, including the number of past and future measurements. In the following simulations, we explore both imputation models (random forest or multinomial) and different sets of predictors: only the previous measurement (P1), the previous and next measurements (PF1), the five previous measurements (P5), five measurements in the past and five in the future (PF5), all other measurements (all), and all previous measurements (past).

### Variable length Markov chains (VLMC)

*Variable length Markov chains (VLMC)* are a type of Markovian model that does not rely on a predefined constant number of time points to predict a current situation. Instead, the number of past states needed to summarize the entire past depends on each specific situation.

According to Gabadinho and Ritschard (2016), two algorithms are commonly used to fit a VLMC model: Learn-PSA (Ron et al. 1996; Bejerano and Yona 2001) and the context algorithm (Rissanen 1983; Bühlmann and Wyner 1999). These algorithms start by considering maximum-length subsequences and then compare the conditional distribution of a subsequence with that of its suffix, which is the subsequence without the last state. If the conditional distributions are sufficiently close, the conditional distribution of the suffix is kept instead of that of the entire subsequence. The two algorithms differ in the criteria used to compare the distributions. The criterion implemented in Learn-PSA is based on the ratios between the conditional probabilities. The idea is that if at least two probabilities are sufficiently different, the conditional distribution of a subsequence cannot be approximated by that of its suffix. The criterion used in the context algorithm is based on the differences in deviance between the conditional distribution induced by the subsequence and its suffix of maximum length. If the difference exceeds a given $\chi ^2$ quantile, the distribution of the current subsequence is chosen.

**Fig. 1:**
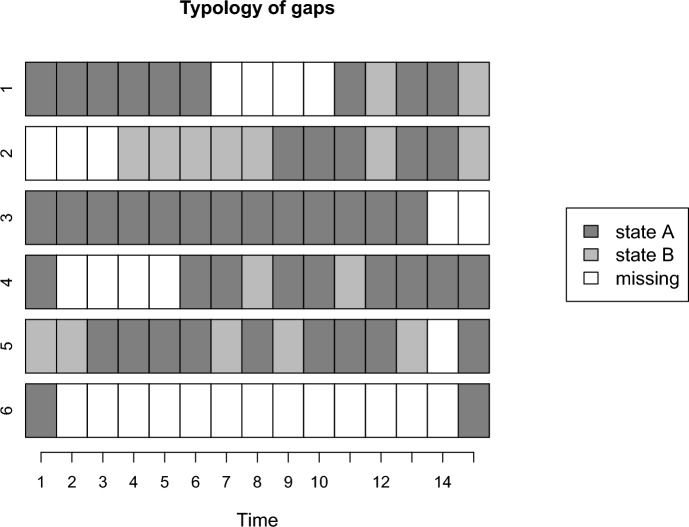
Typology of the different types of missing data gaps according to the *MICT* algorithm considering two predictors from the past and the future: 1. Internal gap, 2. Initial gap, 3. End gap, 4. Left-hand side gap, 5. Right-hand side gap, 6. Both-hand side gap

*VLMC* models can be used to impute missing data. Missing data gaps divide sequences into subsequences. For example, the first sequence in Fig. 1 is divided into two subsequences, one of length six and one of five. A *VLMC* model is fitted to the dataset consisting of all observed subsequences. The missing data gaps are then filled from the left based on the probabilities induced by the subsequence preceding the missing data to be imputed. In practice, one value is drawn to impute the first missing value of sequence 1 based on the probabilities induced by the six-state subsequence between times 1 and 6, and then the second missing value is imputed based on the subsequence consisting of the six observed states and the imputed value at time 7. For initial missing data gaps, such as the one shown in sequence number 2 in Fig. 1, the first missing data is imputed based on the distribution of states in the dataset. The process is then similar to other types of gaps.

We considered VLMC models fitted using either Learn-PSA or the context algorithm. Each case was then configured following the procedure suggested in Gabadinho and Ritschard (2016), which involves identifying the value that minimizes the AIC. For Learn-PSA, we tested the following thresholds 1, 1.05, 1.1, 1.2, or 1.5. For the context algorithm, we tested the following $\chi ^2$ quantiles: 0.1, 0.05, 0.04, 0.03, 0.02, 0.01, or 0.001.

### Multiple imputation for categorical time series (MICT)

The *Multiple Imputation for Categorical Time Series* (*MICT*) algorithm, introduced by Halpin (2012, 2013), handles missing data gaps, which are the typical form of missing data in longitudinal datasets, by imputing them recursively from their edges.

*MICT* uses a statistical model for imputation. It was originally developed using a multinomial model, but we have also explored its use with random forests. To impute a gap of missing values, the algorithm includes some past or future time points. In addition, it has the ability to include fixed covariates (e.g., gender) and time-varying covariates (e.g., number of children).

The algorithm recognizes six patterns of missing data, each of which is handled slightly differently. Assuming two future and past time points are used for imputation, Fig. 1 shows six sequences consisting of two states (A and B) and fifteen time points, each illustrating one of these patterns.

**Fig. 2:**
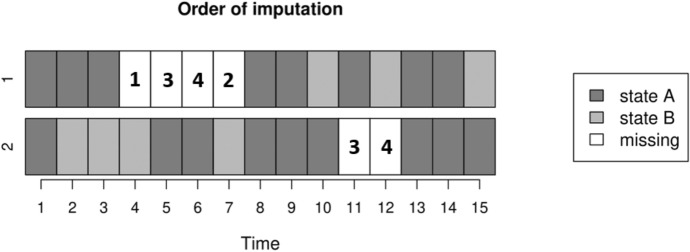
Illustration of the imputation order when the *MICT* algorithm is applied to two sequences with gaps of different lengths

Sequence 1 illustrates an internal gap characterized by at least two observations both before and after the gap. In such cases, the MICT imputation process fills gaps recursively from their edges. Figure 2 shows the order of imputations for two toy sequences with gaps of different lengths. Specifically, imputations are performed using a statistical model, such as a multinomial model. In our example, this model includes the two previous values in the sequence-whether observed or previously imputed values-and the two subsequent values. For example, the first missing value of sequence 1 in Fig. 2 is imputed with a model that uses the two values before the gap (i.e., the states at times 2 and 3) and the two values after the gap (i.e., the states at times 8 and 9). This multinomial model is first estimated using similar fully observed patterns within the dataset. For example, the first trajectory provides one observation: predicting the state at time 10 based on the states at times 8, 9, 14, and 15, while the second trajectory provides six observations: predicting the state at time 3 using the states at times 1, 2, 7, and 8 as predictors, and so on until predicting the state at time 10 based on the states at times 8, 9, 14, and 15.

Once all internal gaps are imputed, attention turns to the initial and terminal gaps. These gaps are exemplified in sequences 2 and 3 of Fig. 1. Given that initial gaps have only one edge, imputation starts from the far right to the left, using predictors from the future since there are no observed data points in the past. Conversely, terminal gaps employ the reverse strategy, imputing from the far left to the right.

Finally, the rarest cases-left-, right-, and both-hand side gaps-are addressed last. Left-hand side gaps (as shown in sequence 4 of Fig. 1) involve sufficient observations for the imputation model after the gap, but insufficient observations before the gap (only one in our example). In these instances, the algorithm considers only one time point in the past instead of two. Right-hand side gaps, on the other hand, feature enough observations before the gap but not after, while both-hand side gaps lack adequate information both before and after.

The *MICT* algorithm therefore takes two three arguments: the imputation model, which can be a multinomial model or a random forest, and the variables in the past and in the future to include. We used the following sets of predictors in the simulations: the previous measurement only (P1), the previous and next measurements (PF1), the five previous measurements (P5), five past measurements, and five future measurements (PF5).

### MICT-timing

Having explored the intricacies of the *MICT* algorithm, we now turn our attention to the *MICT-timing* algorithm, which extends the capabilities of *MICT* by considering the temporal dimension of missing data within trajectories.

It modifies the original *MICT* algorithm in two ways. First, the imputation of gaps of the same length is done separately depending on *when* they occur. Second, only similar fully observed patterns in a time frame around the missing data to be imputed are used to estimate the imputation model. As a result, the transition rates are specific to a time frame.

An additional parameter specifies the radius of the time window. A radius of zero includes only observations that occur at the same time as the missing data to be imputed. A radius of one also includes patterns that occur one time point before or after. Finally, a radius equal to the length of the sequence minus one is equivalent to the original *MICT* algorithm.

**Fig. 3:**
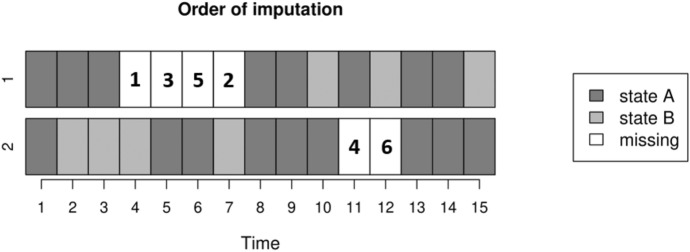
Illustration of the imputation order when the *MICT-timing* algorithm is applied to two sequences with gaps of different lengths

Figure 3 illustrates the difference between the two algorithms. The *MICT* algorithm imputes gaps of the same length simultaneously, regardless of their location. For example, only one imputation model is built to impute the missing values labeled “3” and “4”, because after “1” and “2” are imputed, “3” and “4” are both the first missing values of a gap of length 2. With the *MICT-timing* algorithm, two separate models are fitted, one to impute the missing value labeled “3” and another for the missing value labeled “4”, since they do not occur at the same time. Furthermore, only the observations that fall within the time frame of the predefined radius are used to fit the imputation models. For example, with a radius of length 1, the second sequence provides only three observations (times 3, 4, and 5 and their predictors) instead of the five with the *MICT* algorithm for imputing the first missing data (labeled “1”), and the first trajectory provides no observation.

The *MICT-timing* algorithm therefore takes four arguments: the imputation model, the variables in the past and in the future to include, and the radius. We have included the same parametrization as for the *MICT* algorithm with a radius of either 0 or 5.

## Simulation framework

This article aims to evaluate several imputation methods through simulations, with each step explicitly aligned with life course theory to ensure the relevance of the resulting recommendations.

The simulations were conducted using several univariate longitudinal datasets, each of which embodies different characteristics encountered in life course research. The datasets were resampled with replacement, and missing data were randomly generated using three models designed to replicate common patterns observed in longitudinal studies. These missing datasets were then imputed using the imputation methods and their different parametrizations. Finally, the quality of the imputations was assessed in terms of bias, coverage, and variance induced on estimands relevant to life course research.

In each scenario (dataset x missing data model), we performed one hundred iterations. This decision was made to strike a balance between computational time and the size of the Monte Carlo standard errors, which assess the uncertainty arising from the finite number of simulations.

The presentation of the simulation framework is structured as follows: First, we outline the selection of datasets and their characteristics. Next, we describe the missing data generation models. Finally, we discuss the criteria used to evaluate the methods.

### Data

The life course paradigm emphasizes understanding how trajectories unfold over time, and data aim to describe these trajectories. Such data can be characterized in different ways, depending on the research questions and the data at hand. In this study, we relied on six complete real-world datasets to encompass different configurations. Before detailing these datasets, we first outline the considerations that guided their selection.

First, some datasets have very strong temporal aspects, meaning that some states or transitions typically occur at certain points in time. For example, most people live with their parents at age 10, which is generally not the case at age 40. In other databases, the timing aspect is less pronounced. For example, in occupational trajectories, changes in work status can occur at any time between the ages of 20 and 40. There is often a strong temporal aspect  when trajectories are described in terms of calendar time rather than process time (typically age).

Second, trajectories differ in the characteristics of their transitions. Some processes have few transitions, while others are more volatile. In addition, certain processes follow a strict order and rarely return to a previously visited state. This pattern is common in developmental trajectories or when certain transitions, such as from dead to alive, are impossible.

Third, the encoding of the process itself may vary. While time is sometimes measured on a monthly scale, generally resulting in longer sequences, it is often measured on a yearly basis. Furthermore, the level of detail used to describe the possible states that occur in the trajectories may vary. More complex trajectories are generally more difficult to impute because of the increased potential for incorrect imputation. These states may be ordered or unordered. With unordered states, imputations are either correct or incorrect, while with ordered states, an imputation may be more or less correct. Finally, the number of cases typically varies from study to study. Small datasets are more prone to overfitting.

In this study, we selected six datasets to illustrate the common configurations of aspects of life course research. Before detailing how they were constructed, we first summarize their main characteristics. Table 1 presents these datasets, providing a brief description and the data source, as well as information on the number of cases, the time unit, the sequence length, the states and their frequencies. The table also includes information on the total percentage of transitions between time points and the average number of visits to previously visited states (AVN), which measures the recurrence of previously visited states (Pelletier et al. 2020). It varies from one, when the return to previously visited states never occurs, to half the sequence length, when the process strictly alternates between two states.

**Table 1:** Datasets and their main characteristics. For each of the six datasets, the source of the data, the timing aspect, the number of trajectories (n), the time unit, the length of the trajectories, the average number of visits to visited states (ANV), the percentage of transition, the detail of the states (which are mutually exclusive) with their frequencies in the dataset, are shown

Trajectory	Data source	Timing	*n*	Time unit	Length	ANV	Transition (in %)	States	Freq. (In %)
Professional	SHP (retrospective)	Strong/process time	3382	Year	26	1.33	10.1	Full-time work	50.1
								Part-time work	10.3
								Non-working	12.6
								Education	27
Cohabitational (4 states)	SHP (retrospective)	Strong/process time	3710	Year	26	1.08	9.8	With child	34.3
								With partner, no child	16.6
								With parent(s)	32.2
								Other	16.8
Cohabitational (8 states)	SHP (retrospective)	Strong/process time	3710	Year	26	1.08	11.1	Living alone	13
								With both parents	27.1
								With one parent	5.2
								With partner, no child	16.6
								With partner and child	32.7
								With child, no partner	1.6
								With relative(s)	1.1
								Other	2.7
Civil status	SHP (panel)	Weak/calendar time	2324	Year	21	1.01	1.6	Married	59.1
								Separated	1.1
								Divorced	6.8
								Widowed	3.1
								Single, never married	29.9
Health satisfaction	SHP (panel)	Weak/calendar time	1259	Year	21	3.16	36.5	Low	3.7
								Average	11.4
								High	49.3
								Very high	35.6
School-to-work transition	mvad	Strong/process time	712	Month	72	1.27	3.6	School	8.5
								Further education (FE)	16.2
								Higher education (HE)	11.7
								Training	10.3
								Employment	44.7
								Joblessness	8.6

**Fig. 4:**
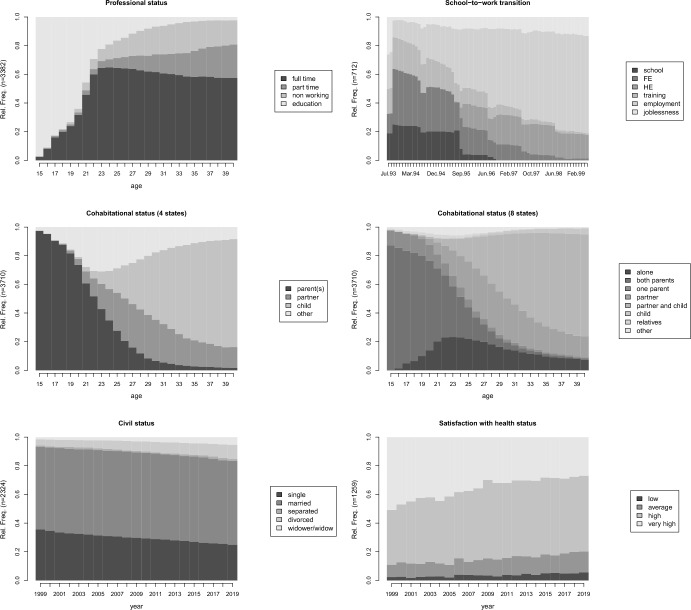
Chronograms of the datasets

Figure 4 regroups the chronograms of the trajectories in each of the six datasets. These chronograms show the proportion of individuals in each state at each time point. Among other things, it illustrates the temporal regularities in the trajectories and the overall frequencies of each state. Additionally, this information is summarized in Table 1.

More precisely, we constructed three datasets using a retrospective life history survey of the Swiss Household Panel (SHP) (Tillmann et al. 2016): one professional and two cohabitational trajectories. They all encode trajectories measured in process time and have strong temporal regularities as shown in Figure 4. The professional and cohabitational trajectories have similar characteristics, except that the return to previously visited states is more frequent in the professional trajectories. The two codings of cohabitational trajectories with four and eight states were selected to demonstrate the effect of coding on imputation. These three databases contain the largest number of cases. We built two datasets using the prospective survey of the yearly Swiss Household panel from 1999 to 2019. These trajectories are measured on calendar time with much weaker timing regularities than the previous ones. The first database encodes civil status over time, while the second focuses on health status satisfaction. These trajectories differ in their general transition rates. While civil status trajectories are highly stable, health satisfaction is the most volatile (see the percentage of transition displayed in Table 1).

Finally, we also considered transitions from education to employment in Northern Ireland (McVicar and Anyadike-Danes 2002). The trajectories are measured monthly, resulting in longer and more stable trajectories, because transitions are less frequent with smaller time units. Notably, this dataset exhibits strong time heterogeneity, with school transitions occurring predominantly during the summer months.

In summary, we selected six datasets to represent the diversity of data characteristics encountered in life course research. We aimed to capture differences in temporal regularities, overall transition rates, ability to visit previously visited states, time measurement (monthly or annual data), and coding detail. In addition, sample size varies across datasets, and this aspect is further explored in the simulation, as it could influence any of the above configurations of data characteristics.

### Missing data generation

We describe the models used to generate missing data in the six complete datasets. The goal of these models is to simulate realistic missing data patterns, with three different generating models chosen to represent common scenarios in life course research. Notably, each of these models can affect the performance of the algorithms in different ways. Specifically, these models simulate a MAR mechanism, attrition, and a MAR mechanism within a small sample size environment.

The remainder of the presentation is organized as follows. First, we describe the simulation framework before presenting each model separately. Finally, we present descriptive statistics of the generated missing data patterns to ensure that the models work as intended.

In all models, we started by randomly selecting 40% of the observations that remain complete. This step ensures a sufficient number of complete sequences. Missing data on these selected sequences were then simulated using one of the three models. If more than 75% of the data on a sequence were missing, the simulation process was repeated for that sequence. This procedure ensures that all sequences have sufficient information.

**MAR model** This model simulates a MAR mechanism in which missing data tend to manifest as gaps, and the occurrence of a gap depends on the previous observed state. The purpose of this simulation is to evaluate the ability of each algorithm to handle a typical longitudinal MAR mechanism.

At the initial time point, where no previous situation is available, the probability of a gap starting is 0.06. For subsequent time points, the probability of missingness depends on the previous state. For certain predefined states (see Table A1 for the list), the probability of starting a gap is 0.20, while for other states it is 0.03. We chose these states to ensure that missing data can occur at any time. Once a gap is initiated, it has a 0.66 probability of continuing and therefore a 0.34 probability of ending.

**Attrition model** This model simulates an attrition process in which individuals stop participating in a prospective survey. Consequently, attrition induces missing data for all subsequent waves, which persists until the end of the sequence. This is a very common pattern of missing data in life course research. Sequence three in Fig. 1 exemplifies attrition, where an individual stopped participating in the survey in 2018 and did not participate thereafter.

In terms of imputation methods, the operation of *FCS* and *MICT-timing* in the case of attrition is very similar. Both algorithms, like *MICT*, can only use past observations. *VLMC* should not be affected by attrition since it only uses past information.

The model works like this. Starting from the middle of the sequence, the probability of triggering attrition depends on the previous state, as in the MAR model. For simplicity, we use the same predefined list of states as before. The probability of starting attrition is 0.10 if the previous state is in the list and 0.015 otherwise. These percentages ensure an overall amount of missing data in the simulations close to the MAR model.

**Small sample** The final model aims to examine the effect of sample size on the performance of each method. We expect small sample sizes to affect each imputation method differently. On the one hand, sample size affects the predictive performance of multinomial models (de Jong et al. 2019). On the other hand, random forests work well with small samples (Biau and Scornet 2016). Also, the *MICT* imputation algorithm uses comparatively more observations to estimate the imputation model than the *FCS* or *MICT-timing* algorithms. Therefore, *MICT* may be more robust to a reduction in sample size.

This procedure randomly selects 200 cases from a dataset before generating missing data according to the MAR model.

In summary, the missing data generation models aim to simulate the missing MAR mechanism. The models also aim to document attrition, a typical pattern for longitudinal data, and small sample size behavior. Table A2 shows the (average) percentages of complete sequences and the total (average) percentages of missing data generated by these models. The overall percentages of missing data are relatively consistent across the datasets for the MAR and small sample models. The percentages of complete sequences depend on the length of the sequences, with lower percentages for longer sequences. The attrition process creates longer gaps of missing data than the other two mechanisms. The percentage of missing data is lower for the professional and health satisfaction datasets because the states with a higher probability of triggering a missing value are rare at the end of the trajectories. However, we kept it the same for consistency with the first process.

### Evaluation criteria

Following an extensive literature review, Studer and Ritschard (2016) identified three pivotal dimensions for analyzing longitudinal data through a life course lens. These dimensions formed the basis for selecting the targets of our simulation study, referred to as “estimands” throughout the article. We assessed the multiple imputation methods based on the biases, coverages, and variances they induce on each estimand. Bias, coverage, and variance are standard performance measures used to evaluate the effectiveness of multiple imputation methods (Oberman and Vink 2023).

We first describe the three key dimensions highlighted by Studer and Ritschard (2016), together with the estimands we selected based on these three key dimensions, which serve as the targets in the simulation framework. Finally, we explain how the multiple imputation methods were specifically evaluated, focusing on bias, coverage, and variance.

Three main aspects are of interest when studying longitudinal data from a life course perspective. First, the timing of events or states is crucial, as the consequences of a situation often depend on when it occurs. For example, experiencing unemployment at age 15 versus age 50 has different consequences. We assess this dimension by examining the frequencies of states at each point in time.

The second aspect of interest in life course research is duration, which refers to the consecutive time spent in a particular state. Duration is important because it often reflects the impact of exposure to a particular situation, such as extended periods of unemployment. To assess this aspect, we use a second set of estimands: the average spell length in each state.

Finally, sequencing, or the order of different states, is another crucial aspect of interest in trajectories. It encapsulates the dynamics of trajectories, which can have lasting consequences. For example, the sequence ”unemployed followed by employed” may have different implications than ”employed followed by unemployed”. Specifically, we analyze the relative risks of transitions computed from sequences of different states.

In summary, we focus on three pools of estimands to assess the quality of imputations tailored to three aspects of interest in life course research, namely timing, sequencing, and duration. Table A3 shows, for each combination of dataset and missing data procedure, the number of estimands related to the three aspects.

When evaluating the quality of imputation methods, three performance measures are generally considered (Oberman and Vink 2023). First, imputation methods should have minimal bias, which is the difference between the estimated value and the “true” value. Second, they should provide adequate coverage, that is, the correct proportion of confidence intervals that contain the true value, to ensure correct inference. Finally, when multiple methods provide adequate coverage, those that yield smaller variance should be preferred. For each estimand, we examine these three performance measures.

For each estimand, its estimated value and variance are calculated for each imputed dataset and then combined using Rubin’s rule. The variance is computed using 1000 resampling iterations.

All calculations were done using the R statistical environment. The *mice* (Van Buuren and Groothuis-Oudshoorn 2011) and *PST* (Gabadinho and Ritschard 2016) packages were used to apply the *FCS* and *VLMC* imputation algorithms, respectively, while *seqimpute* (Emery et al. 2024) was used to apply both *MICT* and *MICT-timing*. The computation of the performance measures was done with the *TraMineR* package (Gabadinho et al. 2011). The *rsimsum* package (Gasparini 2018) was used to compute the Monte Carlo errors and their plots.

## Results

We divided the analysis of the results into two steps. First, we analyzed the different parametrizations for each algorithm. Then we compared the algorithms using the best performing parametrizations. The goal is to derive recommendations for the most appropriate algorithm(s) in life course research.

We examined three sets of estimands related to duration, timing, and sequencing. We measured the bias, coverage, and variance induced by each imputation method on each estimands. Since there were no clear differences in terms of variance, we focus on bias and coverage.

To streamline the presentation, details on the parametrization of the algorithms are provided in Appendix B. We highlight three important results from this section. First, the optimal parametrization may differ between the attrition process and the other two processes. In general, the attrition process is better handled with more variables included in the imputation models. Second, the best parametrization for FCS multinomial varies significantly across scenarios, while parametrizations for other algorithms are more consistent. Finally, MICT-timing performs better with a shorter time frame for datasets with time heterogeneity. Table 2 summarizes the selected parametrizations for each algorithm, which are the parametrization that provide minimal bias, most often appropriate coverage and minimal variance.

**Table 2:** Configuration(s) of each algorithm selected for comparison

Algorithm	MAR/small	Attrition
FCS multinomial	Five observations both in past and future	Five past observations
FCS random forest	Every other observations	Every past observations
MICT multinomial	Five observations both in past and future	Five past observations
MICT random forest	Five observations both in past and future	Five observations both in past and future
MICT-timing multinomial	Timeframe of radius 0 with	Timeframe of radius 0 with
	One observation both in past and future	One observation both in past and future
MICT-timing random forest	Timeframe of radius 0 with	Timeframe of radius 0 with
		Five observations both in past and future
VLMC	Learn-PSA	Learn-PSA

**Fig. 5:**
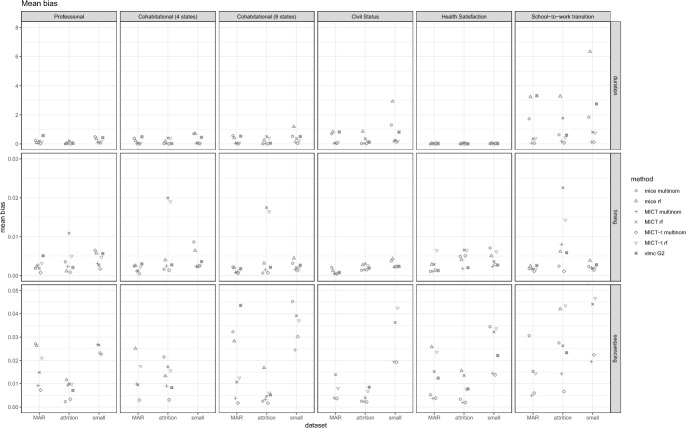
Comparison of the mean absolute bias between the best parametrization of each algorithm. Each panel displays the results of a dataset and a criterion. Within each panel, the results are segmented based on the three missing data generation processes

**Fig. 6:**
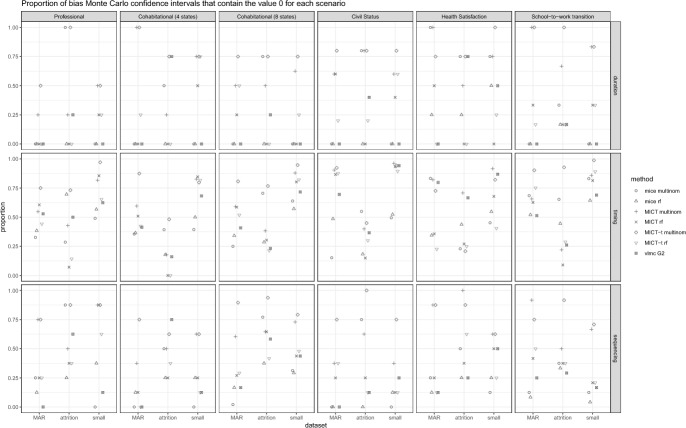
Comparison of the proportion of Monte Carlo confidence intervals that contains the 0 bias between the best parametrization of each algorithm. Each panel displays the results of a dataset and a criterion. Within each panel, the results are segmented based on the three missing data generation processes

**Fig. 7:**
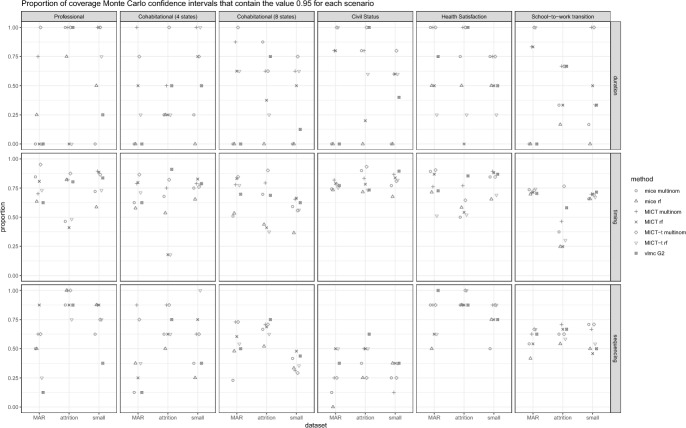
Comparison of the proportion of Monte Carlo confidence intervals that contains the 0.95 coverage between the best parametrization of each algorithm. Each panel displays the results of a dataset and a criterion. Within each panel, the results are segmented based on the three missing data generation processes

We now turn to comparing the imputation algorithms using the best parametrizations identified. The goal is to derive recommendations for the most suitable algorithm(s) in life course research. To facilitate the presentation of the results, we report for each scenario (dataset x missing data generation process) and criterion the mean absolute bias (Fig. 5), the proportion of Monte Carlo intervals containing the value 0 for the bias (Fig. 6), and the value 0.95 for the coverage (Fig. 7). While we lose some information, such as which individual estimand is biased and the differences between overcoverage and undercoverage, it already captures the trends that appear in the detailed results. The bias and coverage of each estimand, along with their Monte Carlo confidence intervals, are shown in the Appendix. We look at each algorithm in turn.

**Fig. 8:**
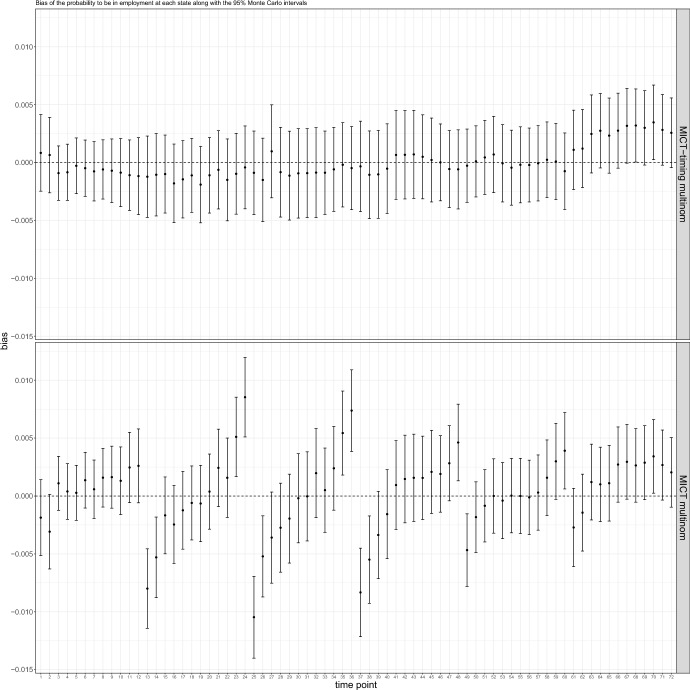
Bias of the probability to be in employment at each of the 72 time points in the school-to-work transition, along with their respective 95% confidence intervals. The top panel presents the results pertaining to MICT-timing, while the bottom panel showcases the outcomes of MICT

*MICT* multinomial exhibits low bias and adequate coverage on most estimands related to duration and sequencing. However, it may show bias and undercoverage on some estimands related to timing with time heterogeneous trajectories. For example, focusing on the school-to-work transition where a MAR process was simulated, we observe a positive bias in the probability of being employed at time 24, followed by a negative bias at time 25 (Fig. 8). This pattern repeats itself every twelve months. This phenomenon can be explained by the fact that most school-to-work transitions occur during the summer months. *MICT* predicts transitions evenly throughout the year, resulting in an excess of transitions to employment throughout the non-summer months and a shortage during the summer months.

The use of *MICT* with random forest models instead of multinomial models reduces the overall quality of the results. This is more pronounced for estimands related to the timing of attrition, where the use of random forest imputation models can show the greater bias, and thus undercoverage, among the algorithms. On the other hand, the differences are reduced for small samples.

*MICT-timing* multinomial outperforms *MICT* multinomial on datasets with time heterogeneity. In particular, the algorithm addresses problems associated with the use of *MICT* in cases of time heterogeneity. In addition, it reduces the bias of some other estimands, such as the sequencing estimands related to professional and cohabitational status in the case of a *MAR* process. On datasets with time-homogeneous transition rates, *MICT-timing* is sometimes beaten by MICT. Furthermore, the application of random forest imputation models shares the concerns raised with the *MICT* algorithm, while not showing a clear improvement in the case of time heterogeneity.

As a reminder, the optimal setting was not consistent for *FCS* multinomial. In MAR and small simulations, except for satisfaction with health status, the parametrization retained for comparison exhibits more biased and undercovered results than other algorithms, such as *MICT-timing* multinomial. Conversely, in attrition simulations, *FCS* multinomial with a predictor in the past stands out as one of the best performing algorithms. In this case, its operation is close to that of *MICT-timing* with a time frame of length 0, leading to similar results.

Although *FCS* random forest occasionally approaches the performance of the best algorithms, as seen on the timing estimands in the attrition simulation based on professional status, it performs inadequately in most scenarios.

Finally, *VLMC* emerges as one of the best performing algorithms in the attrition simulations, although it is outperformed by *MICT-timing* multinomial in specific cases (e.g. the school-to-work transition). For the other two processes, however, *VLMC* generally shows inferior performance. Looking more closely at the results, it appears that *VLMC* performs particularly poorly when it comes to imputing initial time points.

## Discussion

In this study, we conducted a comparative analysis of several imputation methods for univariate longitudinal categorical data and evaluated their optimal parametrizations. In addition, we introduced two extensions of the MICT algorithm. First, we explored the use of random forest models. However, our results indicated that random forest did not offer significant advantages over multinomial regression in the various scenarios studied. Second, we developed the MICT-timing algorithm to address the challenge of heterogeneous transition rates within trajectories. Our results showed that the MICT-timing algorithm achieved its goal and proved to be a viable alternative to the original MICT algorithm, especially when trajectories exhibited time-varying transition rates.

The comparison of imputation methods was based on six real-world datasets, each carefully selected to represent different scenarios encountered in life course research. To evaluate the performance of these methods under different conditions, we simulated missing data using three different models. First, we simulated missing data to reflect a MAR process in which missing data occurred as successive time points with missing data, also known as gaps. In addition, some states were assigned higher probabilities of triggering a missing data gap, reflecting common scenarios observed in life course research. For example, situations such as unemployment or poor health often have a higher risk of being associated with missingness. Second, we designed simulations to assess missing data due to attrition, a common problem in prospective longitudinal surveys. In cases of attrition, only past information is available, which poses a challenge for imputation algorithms. Finally, we conducted simulations to evaluate the performance of each algorithm under conditions of small sample size, providing insight into their robustness in such scenarios. These simulations allowed us to thoroughly evaluate the effectiveness of each imputation method across a range of realistic scenarios encountered in longitudinal data analysis.

From each complete dataset and missing data model, we generated missing data for 100 resampled datasets. These simulated missing data were then imputed with the different algorithms, namely *FCS*, *MICT*, *MICT-timing*, both with multinomial and random forest imputation models, and *VLMC*. We have also included several settings for each of these algorithms. The algorithms and their settings were then evaluated. We measured bias, coverage, and variance on estimands related to three key characteristics of trajectories for life course research, namely timing, duration, and sequencing of successive events.

Among the tested algorithms, MICT-timing multinomial emerged as the most effective imputation method in these simulations. It consistently demonstrated unbiased results and adequate coverage in most scenarios. While its performance was comparable to that of the MICT algorithm under conditions of time homogeneity, it significantly outperformed it under conditions of time heterogeneity, particularly in estimands related to timing. Initially, we hypothesized that in scenarios with small sample sizes, the MICT-timing multinomial might be outperformed by the MICT multinomial because the latter uses more observations for model fitting. However, our simulations did not support this hypothesis. Nevertheless, the Monte Carlo standard errors are larger for small sample sizes, and increasing the number of simulations may provide further insight into potential differences.

The use of random forest as an imputation model did not produce satisfactory results, consistently lagging behind the multinomial model. There are several possible explanations for this behavior. First, random forest may not have been appropriate for the specific missing data scenarios considered. The probability of missing data was determined based solely on the previous time point, which, while a reasonable approximation, may not fully capture the complexity of real missing data relationships. These relationships are likely to depend on more complex patterns of experience. Random forest may be better equipped to account for these complex relationships. In addition, its implementation in MICT and MICT-timing computed probabilities as the proportion of trees predicting a class. However, each tree often predicted the same class, making the proportion of trees unsatisfactory for accurately approximating probabilities. In this context, the adoption of probability forests (Malley et al. 2012) may serve as an alternative to obtain more accurate probability estimates. Additionally, it is worth noting that random forests, and thus MICT random forests, are more robust to reductions in sample size. Therefore, in scenarios with small sample sizes, the results are closer to those of MICT multinomial than the other missing data generation methods.

The *FCS* algorithm with a multinomial imputation model stands out among the best algorithms in the case of attrition and datasets with more transitions, but is less efficient in the other situations. In the case of attrition, its operation closely mirrors that of the *MICT-timing* multinomial with a time frame of length 0. Consequently, both algorithms exhibit similar characteristics in this case. With more transitions, the gain obtained with the core function of *MICT* and *MICT-timing*, which fills gaps recursively from their edges, is less pronounced, and thus *FCS* competes, while with fewer transitions the opposite is true. While it can occasionally perform well, *FCS* random forest is never the best performing algorithm in all scenarios. In fact, it occasionally performs the worst. Therefore, *FCS* appears to be a less efficient imputation algorithm than *MICT* for datasets typical of life course studies.

*VLMC* is effective in imputing attrition, but its performance was generally poor in the context of MAR and small missing data processes. The limitation of VLMC to use only past observations significantly affects the quality of imputations, especially at the beginning of trajectories.

The six datasets used for our comparison were carefully selected to represent the variety of situations encountered in life course research. Before outlining the guidelines, we discuss how the characteristics of these situations influence the imputation process.

First, the datasets differ in their timing structure, indicating different degrees of time heterogeneity in the transitions. As previously noted, the stronger the timing structure, the better the performance of the MICT-timing algorithm compared to MICT. Time heterogeneity also affects VLMC, as gaps of missing data break sequences into sub-sequences that are then realigned to fit the model. However, attrition processes can reduce sequence lengths but do not break them into sub-sequences, and thus have less effect on VLMC. The effect of timing on FCS is less obvious.

Second, the datasets differ in the characteristics of their transitions. For datasets with highly stable trajectories, imputation tends to be easier and requires less information. MICT and MICT-timing algorithms perform significantly better than FCS in such cases, as FCS tends to create artificial transitions. Conversely, imputation is more difficult in volatile datasets, sometimes resulting in impossible transitions. Checking for such inconsistencies in the original data before imputation is crucial, as correcting them after imputation can be cumbersome and may affect consistency with other imputed values.

Third, the datasets differ in their allowance for returns to previously visited states, such as in occupational and marital status histories. This feature does not significantly affect the quality of the imputations or the performance of the algorithms.

Fourth, the datasets differ in their coding, with cohabitation status coded with either four or eight states. More detailed coding of states leads to poorer imputations, with differences between algorithms more pronounced for more detailed coding. However, the ranking of the algorithms remains consistent. Therefore, it is advisable to use the level of detail necessary for the analysis during the imputation process.

Finally, our simulations highlighted the effect of sample size. As sample size decreases, the differences between multinomial and random forest models diminish, although multinomial models still outperform random forest. Larger Monte Carlo standard errors in such scenarios make it difficult to draw definitive conclusions.

### Guidelines

The purpose of this article is to provide guidelines for imputing missing data in longitudinal categorical databases, with a particular focus on datasets commonly used in life course research. Such datasets typically have a moderate number of transitions over time. These guidelines include considerations for both data preparation and the selection of appropriate imputation algorithms.

Before starting the imputation process, it is crucial to prepare the data carefully. Three key aspects deserve particular attention. First, researchers should avoid overly detailed categories, as these will affect the quality of the imputations, regardless of the algorithm used. It is therefore advisable to limit categories to the most relevant distinctions for the research question.

Second, researchers must carefully check and correct the longitudinal coherence of the trajectories. Any inconsistencies or errors in the data, such as impossible transitions, may persist if not corrected before the imputation process begins. Therefore, researchers should correct these errors before proceeding with imputation.

Finally, researchers should carefully identify missing values that might correspond to non-existent data. Since no valid values can exist for these cases, imputation is likely inappropriate, and these instances should remain empty.

When it comes to selecting the imputation algorithm, MICT-timing multinomial emerges as the preferred option, particularly in scenarios characterized by time heterogeneity. A radius of zero and one predictor in the past and future generally yields good results in most simulations. However, increasing the number of predictors can improve performance, especially in the case of attrition. Conversely, in situations where the data do not exhibit time heterogeneity, the MICT multinomial is the recommended choice.

### Limitations

This study includes different datasets, imputation algorithms, and simulations, but it also has limitations. First, we focused on univariate data, but as Bernardi et al. (2019) points out, different life domains should be considered as interdependent. For example, women’s family and career trajectories are often closely linked in many countries. It is therefore advisable to impute these trajectories together. Although we used three different missing data generation processes in our simulations, in practice MAR and MNAR missing data are likely occurring simultaneously. Nevertheless, these processes were sufficient to reveal differences between the imputation algorithms. In addition, our imputation models only considered past or future time points. However, most algorithms can include other covariates, such as sex or cohort information. While the inclusion of such data is critical to improving imputation quality, it is unlikely to change the ranking of the algorithms.

In this study, we have reviewed algorithms for handling missing data in univariate categorical longitudinal data and proposed the MICT-timing algorithm. However, further research is needed to address several key aspects. First, it is essential to explore the interplay between imputation algorithms and subsequent statistical analyses. This includes considering methods for creating typologies of trajectories or estimating models such as multistate or hidden Markov models. Then, as discussed earlier, it is imperative to extend the algorithms outlined in this study to accommodate multivariate longitudinal categorical data. While FCS extends seamlessly to this scenario, the MICT algorithm lacks this capability, despite its efficacy for univariate data. Finally, our focus was only on data that could have been collected, but is missing. Future research should consider all forms of missing information, including cases of non-existent data, such as when an individual is deceased. Furthermore, missing data is only one of the many source of errors. Among others, sampling and measurement errors should also be taken into account, including misclassification due to recall bias in longitudinal studies (see e.g. Pina-Sánchez et al. 2019). A comprehensive framework is therefore needed to deal with all these sources of error.

## Supplementary Information

Below is the link to the electronic supplementary material.

Supplementary file 1 (pdf 4020 KB)

## Data Availability

The Swiss Household Panel datasets analysed in this study are freely available to researchers on acceptance of a user contract from SWISSUbase (https://www.swissubase.ch). Replication files are available from https://github.com/emerykevin/comp_catlong.
