# Supplementary material for: Comparison of imputation methods for univariate categorical longitudinal data
Source: Qual Quant. 2024 Dec 26;59(2):1767–91. doi: 10.1007/s11135-024-02028-z (PMC12104099; doi:10.1007/s11135-024-02028-z)
Supplement: Supplementary file 1 — (pdf 4020 KB) [file 11135_2024_2028_MOESM1_ESM.pdf]

A Tables

Trajectory	States
Professional	education<br>non-working
Cohabitational (4 states)	with a child<br>other
Cohabitational (8 states)	living alone<br>with a child<br>with one parent<br>with a partner
Civil status	separated<br>divorced<br>single, never married
Health satisfaction	low<br>average
School-to-work transition	further education (FE)<br>higher education (HE)<br>training

Table A1: For each datasets, states with a higher probability to trigger a missing value for the second and third process of missing data are detailed.

Process	Dataset	% incomplete sequences	% missing data	mean length gap	% gaps of length 1
MAR	Professional	51.4	10.0	2.8	35.2
	Cohabitational (4 states)	55.4	12.8	2.6	37.6
	Cohabitational (8 states)	51.8	11.1	2.8	36.1
	Civil status	42.1	10.6	2.6	37.3
	Health Satisfaction	38.3	7.3	2.6	37.4
	mvad	58.8	11.5	2.9	34.6
Attrition	Professional	14.8	4.8	8.5	5.3
	Cohabitational (4 states)	40.3	12.9	8.4	4.5
	Cohabitational (8 states)	29.6	10.7	9.4	3.3
	Civil status	23.6	8.4	7.5	5.2
	Health satisfaction	17.6	5.7	6.9	7.1
	mvad	39.1	14.2	26.1	0.9
Small sample	Professional	51.3	10	2.8	35.5
	Cohabitational (4 states)	55.4	12.9	2.7	37.2
	Cohabitational (8 states)	51.8	11.1	2.8	36.2
	Civil status	41.9	10.6	2.7	37.3
	Health satisfaction	38.2	7.4	2.6	36.7
	mvad	58.8	11.7	2.9	34.7

Table A2: Average percentage of incomplete sequences, missing data by dataset, mean length of the gaps of missing data and percentage of gaps of length 1 by dataset and missing data generation process.

Trajectory	Missing process	Duration	Timing	Sequencing
Professional	MAR / small<br>attrition	4	104<br>52	8
Cohabitational<br>(4 states)	MAR / small<br>attrition	4	104<br>52	8
Cohabitational<br>(8 states)	MAR / small<br>attrition	8	208<br>104	48
Civil Status	MAR / small<br>attrition	5	105<br>55	12
Health Satisfaction	MAR / small<br>attrition	4	84<br>44	8
School-to-work<br>transition	MAR / small<br>attrition	6	432<br>216	24

Table A3: Number of parameters for each of the three aspects (duration, timing and sequencing) for each scenario (dataset x missing data process). For the duration aspect, the number of parameters is equal to the number of states (s), for the sequencing s*(s-2) and for the timing s*length of the trajectory. The attrition process differ from the two others regarding the number of parameters related to timing, because the first half of the trajectories is not subject to missing data in this case.

B Algorithms Configuration

In this section, we compare the results of various settings for each algorithm.

FCS multinomial

Figures A1, A2, and A3 illustrate, respectively, the results regarding the mean absolute bias, the proportion of Monte Carlo confidence intervals containing zero bias, and the proportion of Monte Carlo confidence intervals encompassing the target coverage of 0.95, obtained with *FCS* multinomial.

For *MAR* and *small* processes, the optimal setting varies across scenarios and may depend on the specific dataset considered. For instance, the configuration with one predictor both in the past and future is among the best configurations on the professional status dataset, but performs poorly on the mvad dataset, where five predictors both in the past and future prove is best.

In the case of attrition, the use of future observations diminishes imputation quality, as expected. Attrition, being a monotone pattern of missing data, should ideally rely only on past observations with the *FCS* imputation method (see section 4.3 of Van Buuren (2018)). Future observations, being randomly drawn according to the marginal distribution at the beginning of the imputation process, may mislead the *FCS* multinomial model, unable to refine these imputations in subsequent steps. The use of one predictor in the past performs best for cohabitational status coded as four states and mvad, but notably lags behind for professional status and satisfaction with health status, where using five predictors in the past yields superior results.

For the sake of comparison with other algorithms, we have selected five predictors both in the past and future, except for the attrition process, where we selected five predictors in the past. While neither configuration is the best in every case, they present a trade-off.

FCS random forest

As depicted in Figures A4, A5, and A6, employing a significant number of predictors—whether five predictors in both past and future instances or across all other time points within the *MAR* and *small* processes, and considering all past time-points within the *attrition* process—generally results in improved performance, particularly in reducing the magnitude of bias.

As observed in *FCS* multinomial, the inclusion of future time-points tends to deteriorate performance. However, the impact on random forest is less pronounced than what was observed in multinomial models.

Consequently, we selected *FCS* random forest with all potential predictors, except in situations characterized by monotone patterns of missing data, such as attrition, where all past observations are used.

MICT multinomial

Figures A7, A8, and A9 showcase the results for the *MICT* multinomial algorithm.

In the cases of *MAR* and *small* missing data processes, the incorporation of future predictors noticeably enhances the quality of the results. However, for the attrition process, where no observations exist beyond the gaps, the inclusion of future predictors is irrelevant.

Concerning the optimal number of predictors to include in the models, we do not observe any significant differences in most cases linked to *MAR* and *small* processes. On the other hand, increasing the number of predictors may improve the quality of the results in the case of attrition, particularly in terms of parameters related to timing.

Summarizing, we applied *MICT* multinomial with five predictors in the past and the future, which is equivalent in the case of attrition to apply only five predictors in the past.

MICT random forest

Figures A10, A11, and A12 the results of the *MICT* random forest algorithm.

While there are exceptions, primarily associated with the proportion of confidence intervals that contains the null bias, the configuration featuring five predictors in both the past and future consistently ranks among the best in most cases. Similar to *MICT* multinomial, settings with and without future observations are identical in the case of attrition.

Therefore, we kept using *MICT* random forest with five predictors in both the past and the future.

MICT-timing multinomial

Figures A13, A14, and A15 presents the results of the *MICT-timing* multinomial algorithm.

The results show that, apart from satisfaction with health status, the optimal configuration is generally achieved with a timeframe of length 0 and one predictor in both the past and future in most other cases.

Extending the length of the timeframe may diminish the quality of the results in some specific cases, such as the parameters related to timing in the case of a *MAR* process simulated on the professional status trajectories. However, in most cases, the results are close. Moreover, increasing the number of predictors introduces bias in specific cases, such as the parameters related to duration in the case of MAR missing data on the mvad dataset, and most parameters related to duration in the case of the *small* process. This outcome was anticipated, given that multinomial models are susceptible to the impact of small sample sizes (de Jong et al., 2019).

Regarding the satisfaction with health status, augmenting the number of predictors may yield improved results in instances involving attrition processes, such as parameters related to timing in the case of satisfaction with health status.

We considered one predictor in the past and the future with a timeframe of radius 0.

MICT-timing random forest

The results are largely independent of the length of the timeframe (Figures A16, A17, and A18). A closer look to the imputed datasets shows that most imputed values are identical across the different lengths of the timeframe.

Similar to other algorithms based on MICT, distinctions must be made between attrition and general patterns of missing data.

For MAR and small processes, the configuration with one predictor in both the past and future emerges as the best compromise. Increasing the number of predictors may slightly enhance the quality of the results in some cases, such as the timing parameters on the professional status trajectories, but is clearly worse on the scenarios related to the mvad dataset.

Increasing the number of predictors generally enhances results in terms of timing parameters but comes at the expense of duration and sequencing parameters. Conversely, using only past predictors may slightly improve results in terms of duration and sequencing (e.g., on the mvad dataset) but compromises timing parameters.

In the case of attrition, increasing the number of predictors may mitigate bias in specific instances (e.g., parameters related to duration and timing in professional trajectories).

For these reasons, we applied one predictor in both the past and future with a radius of zero, except for the attrition process, where we selected using five predictors in both the past and future with a radius of zero.

VLMC

In most situations, the results obtained with both gain functions are close (Figures A19, A20, and A21). Since fitting *VLMC* with Learn-PSA is marginally better in some cases, we selected it for comparison with the other algorithms.

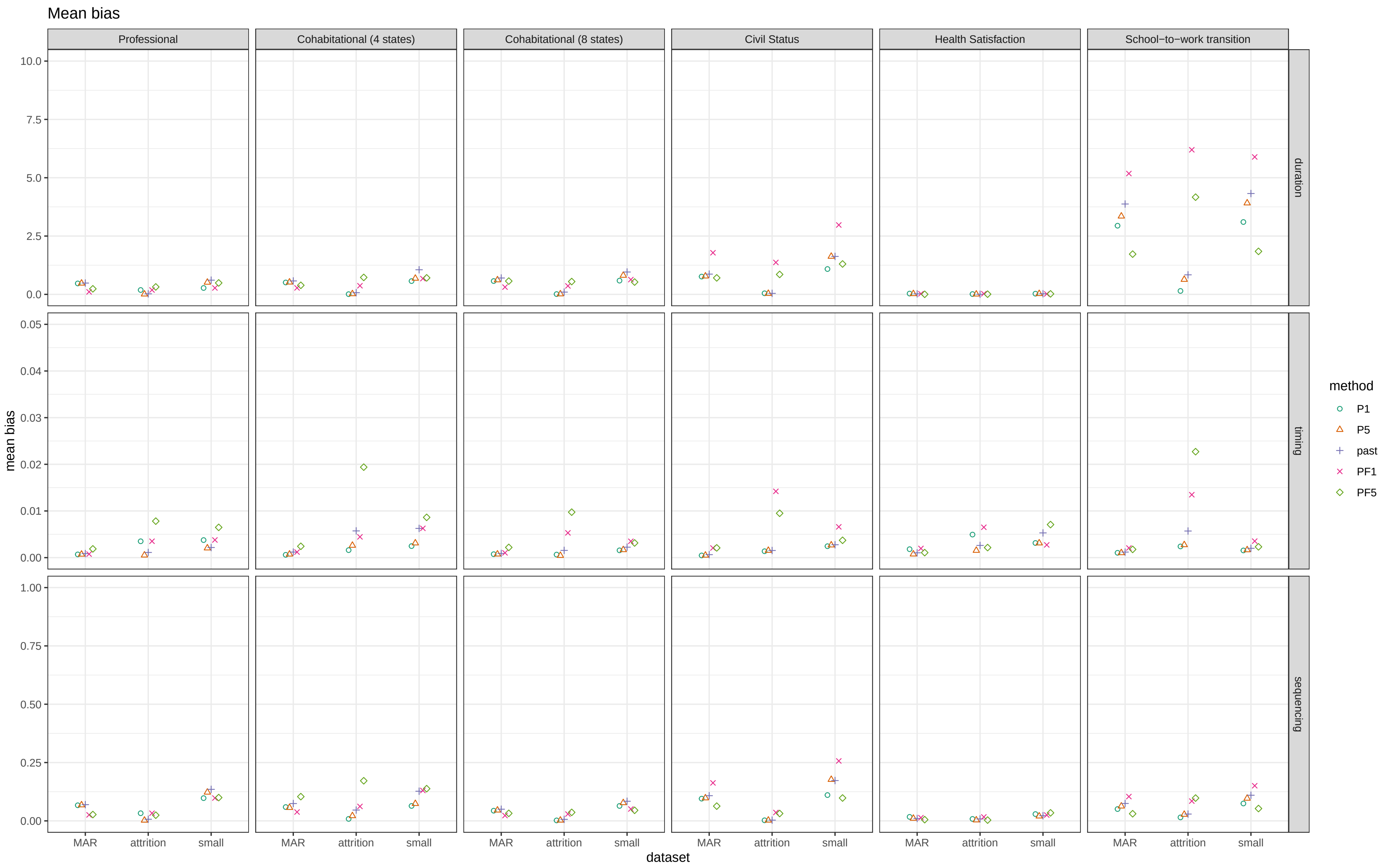

Figure A1: Comparison of the mean absolute bias between the different configurations of the *FCS* multinomial algorithm. Each panel displays the results of a dataset and a criterion. Within each panel, the results are separated according to the three missing data generation processes.

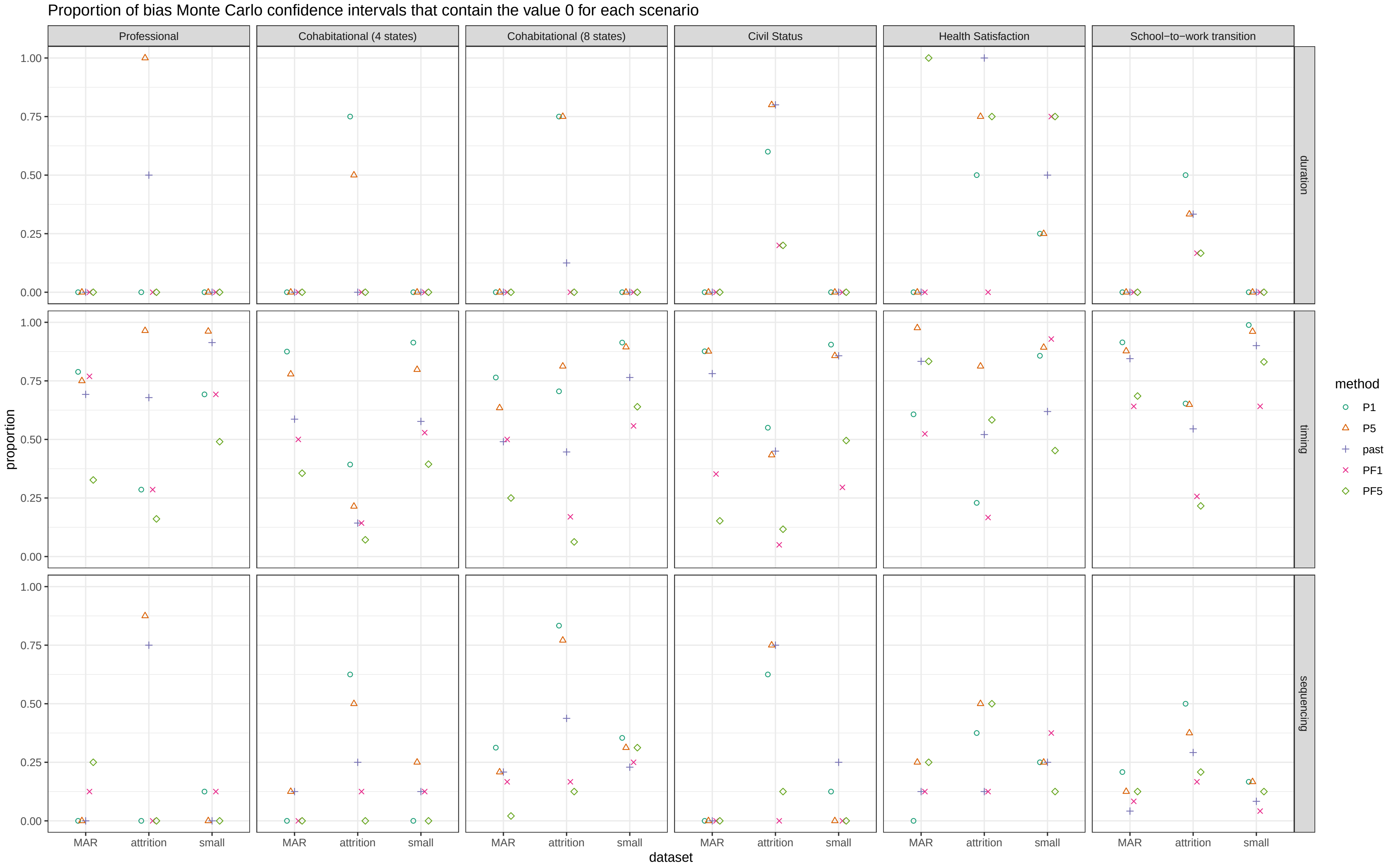

Figure A2: Comparison of the proportion of Monte Carlo confidence intervals that contains the 0 bias between the different configurations of the *FCS* multinomial algorithm. Each panel displays the results of a dataset and a criterion. Within each panel, the results are separated according to the three missing data generation processes.

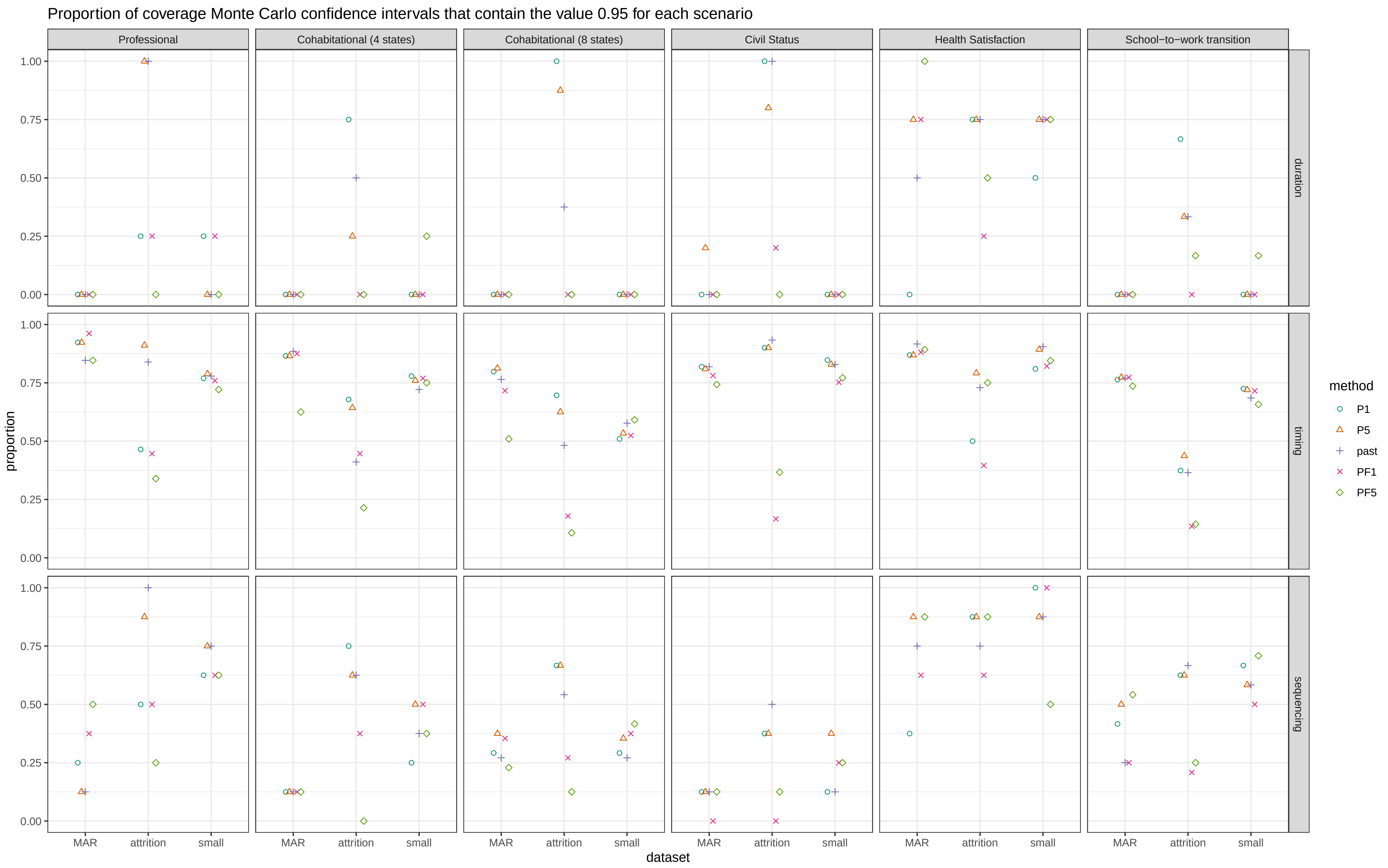

Figure A3: Comparison of the proportion of Monte Carlo confidence intervals that contains the 0.95 coverage between the different configurations of the *FCS* multinomial algorithm. Each panel displays the results of a dataset and a criterion. Within each panel, the results are separated according to the three missing data generation processes.

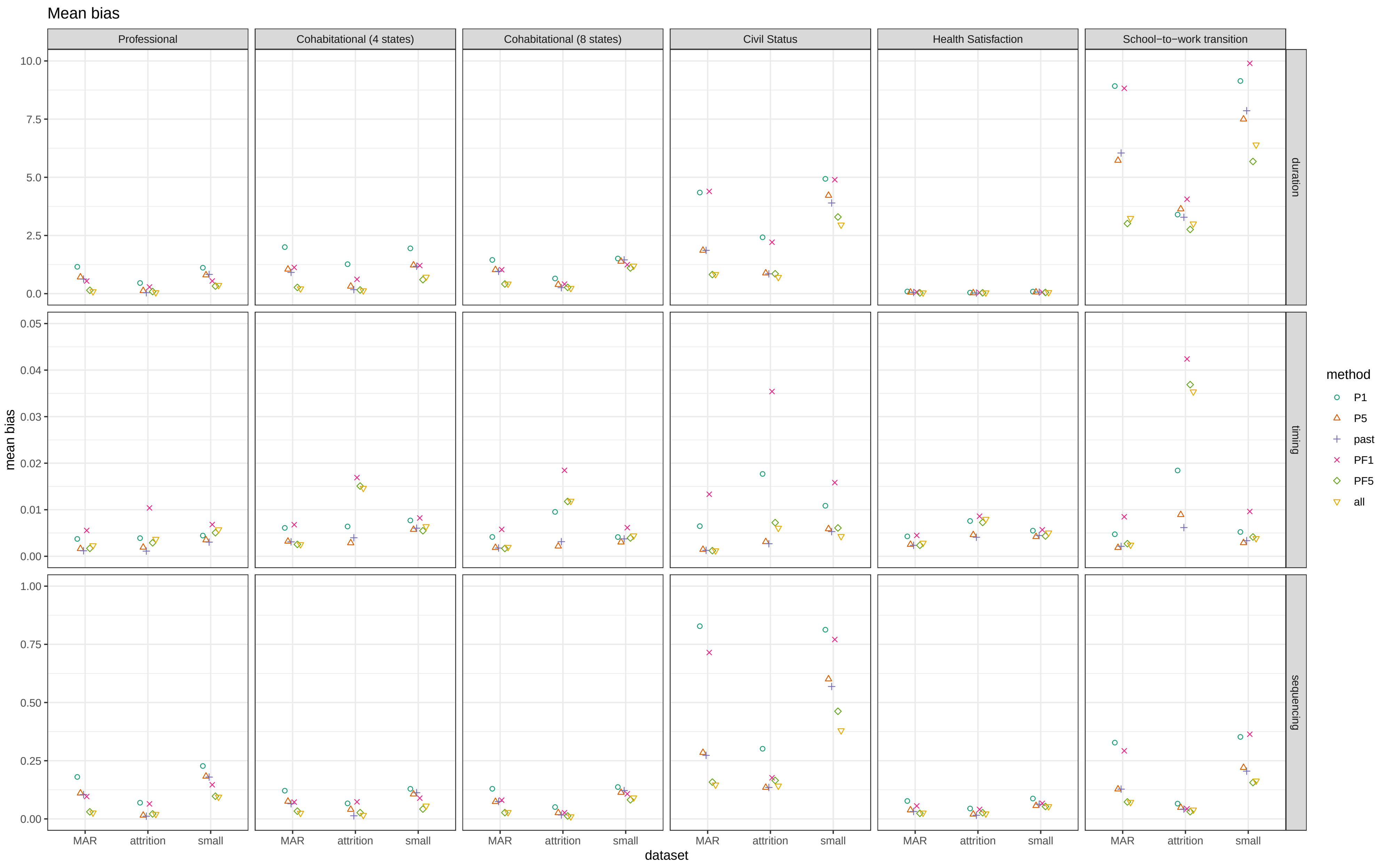

Figure A4: Comparison of the mean absolute bias between the different configurations of the *FCS* random forest algorithm. Each panel displays the results of a dataset and a criterion. Within each panel, the results are separated according to the three missing data generation processes.

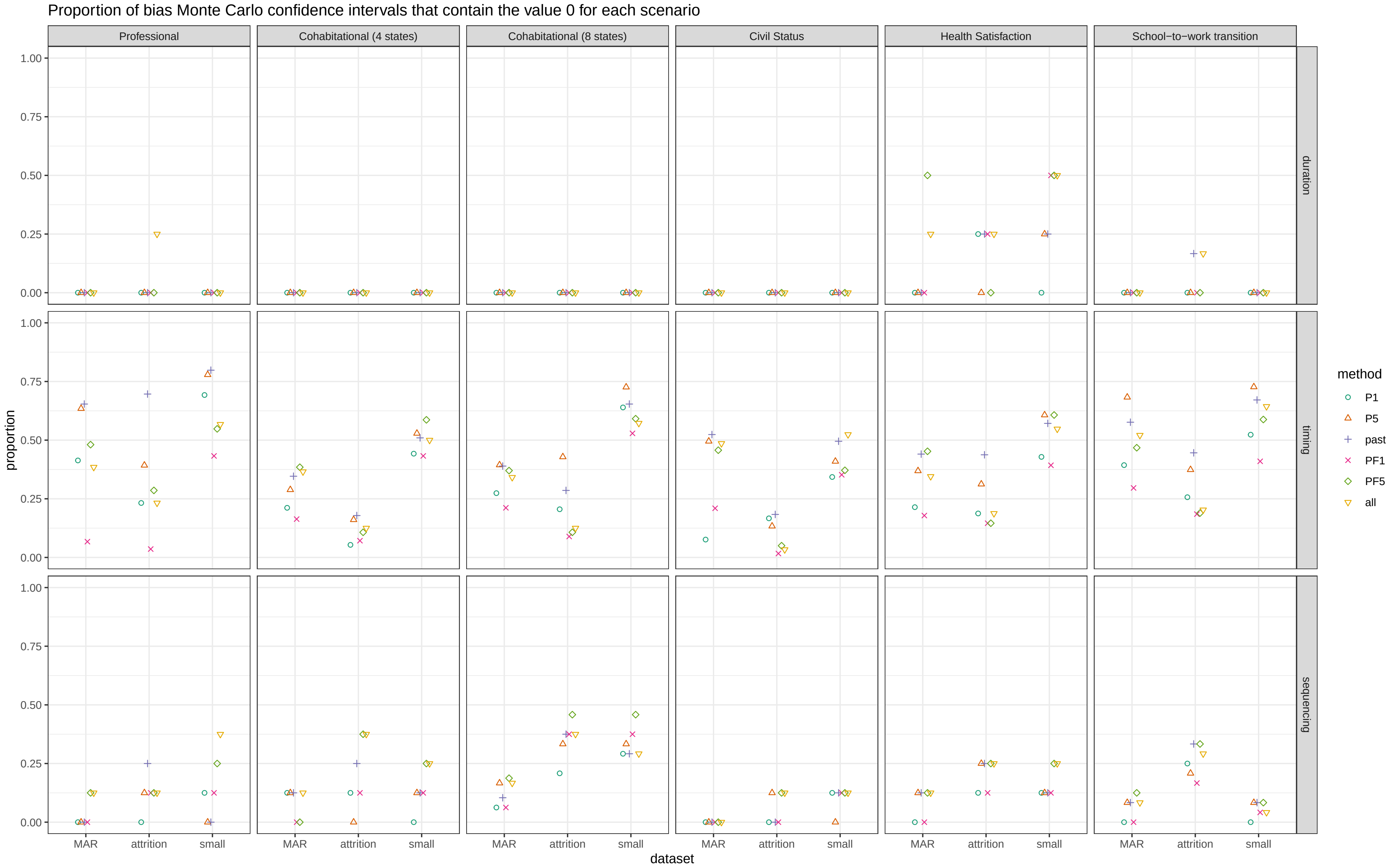

Figure A5: Comparison of the proportion of Monte Carlo confidence intervals that contains the 0 bias between the different configurations of the *FCS* random forest algorithm. Each panel displays the results of a dataset and a criterion. Within each panel, the results are separated according to the three missing data generation processes.

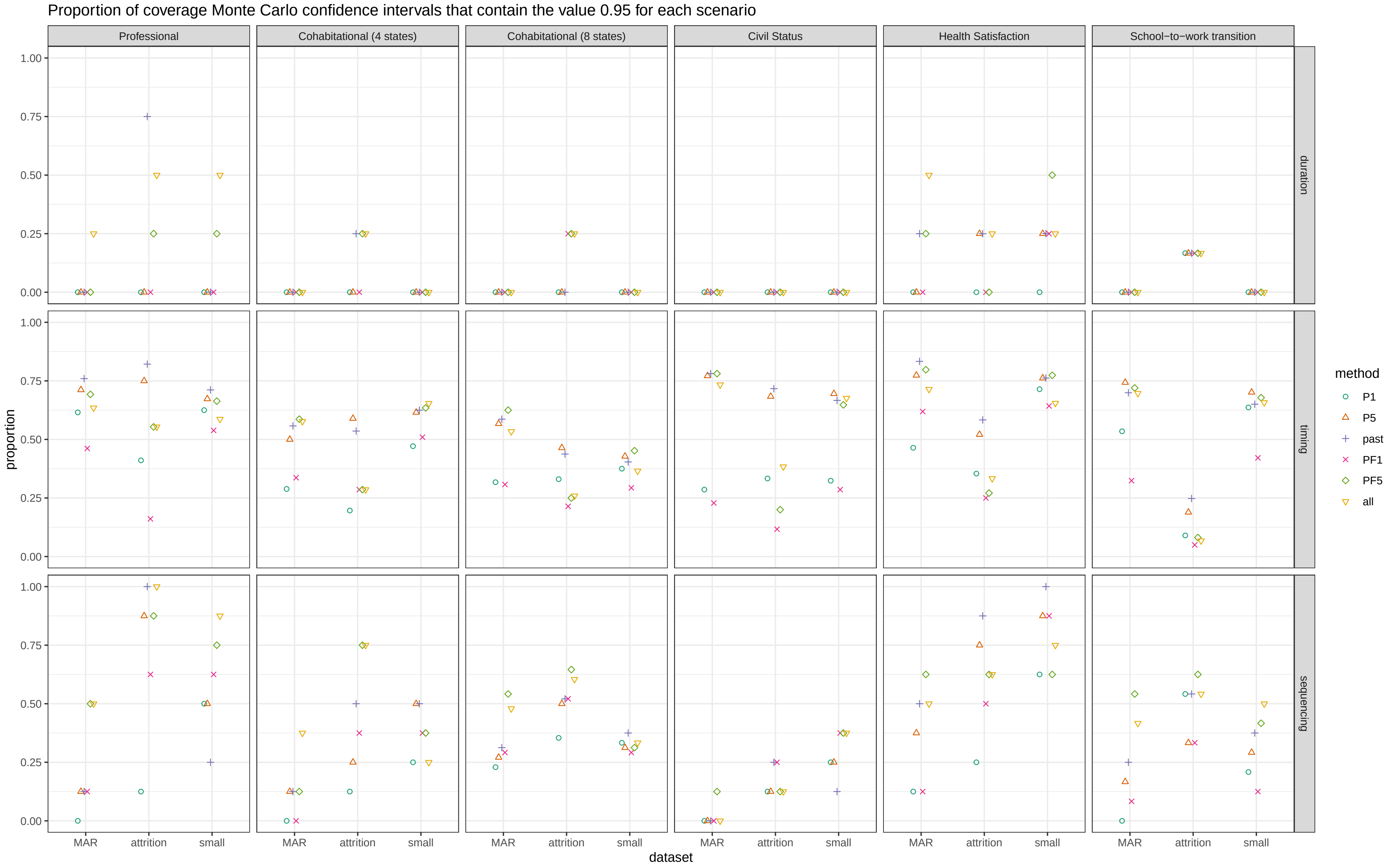

Figure A6: Comparison of the proportion of Monte Carlo confidence intervals that contains the 0.95 coverage between the different configurations of the *FCS* random forest algorithm. Each panel displays the results of a dataset and a criterion. Within each panel, the results are separated according to the three missing data generation processes.

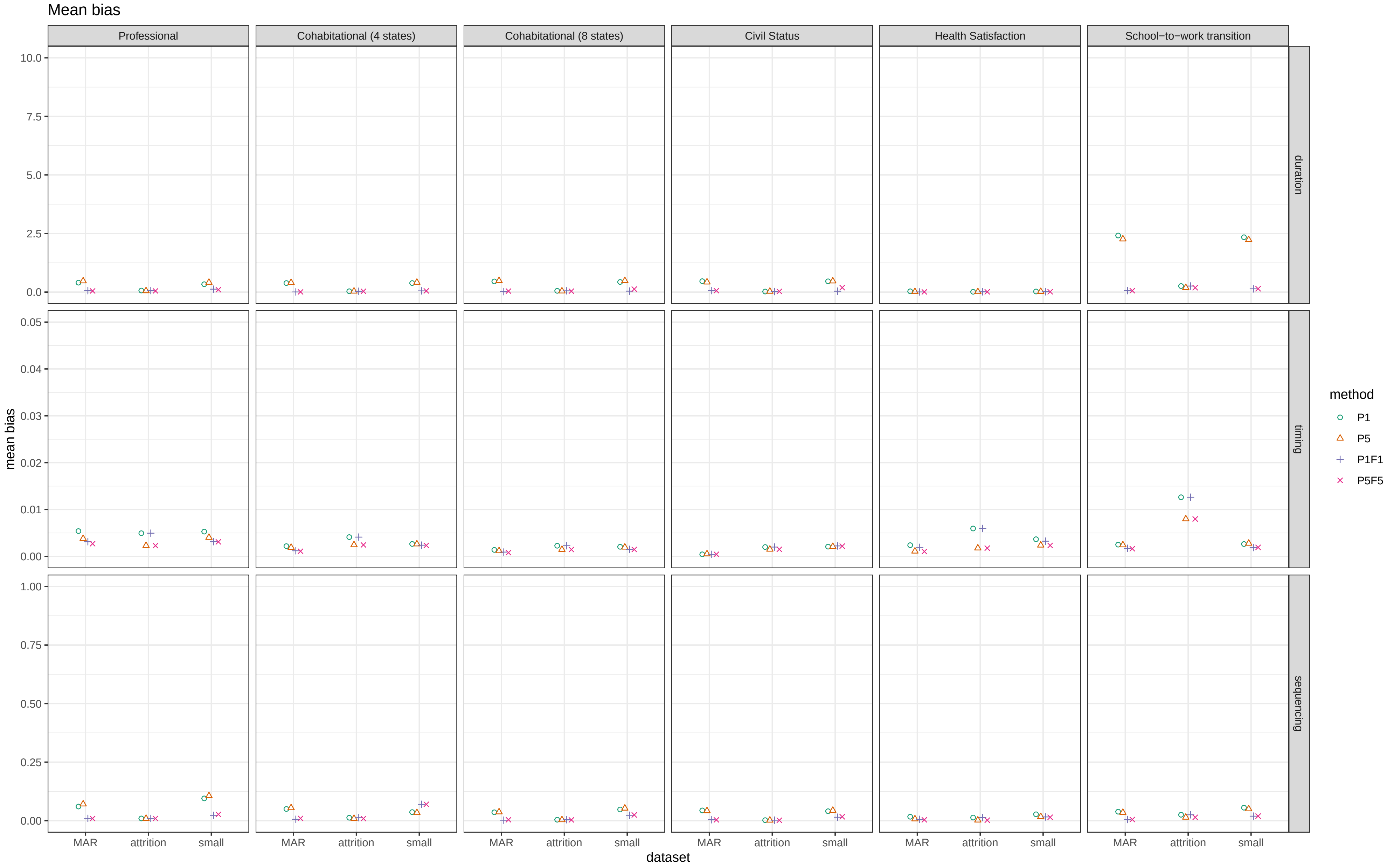

Figure A7: Comparison of the mean absolute bias between the different configurations of the *MICT* multinomial algorithm. Each panel displays the results of a dataset and a criterion. Within each panel, the results are separated according to the three missing data generation processes.

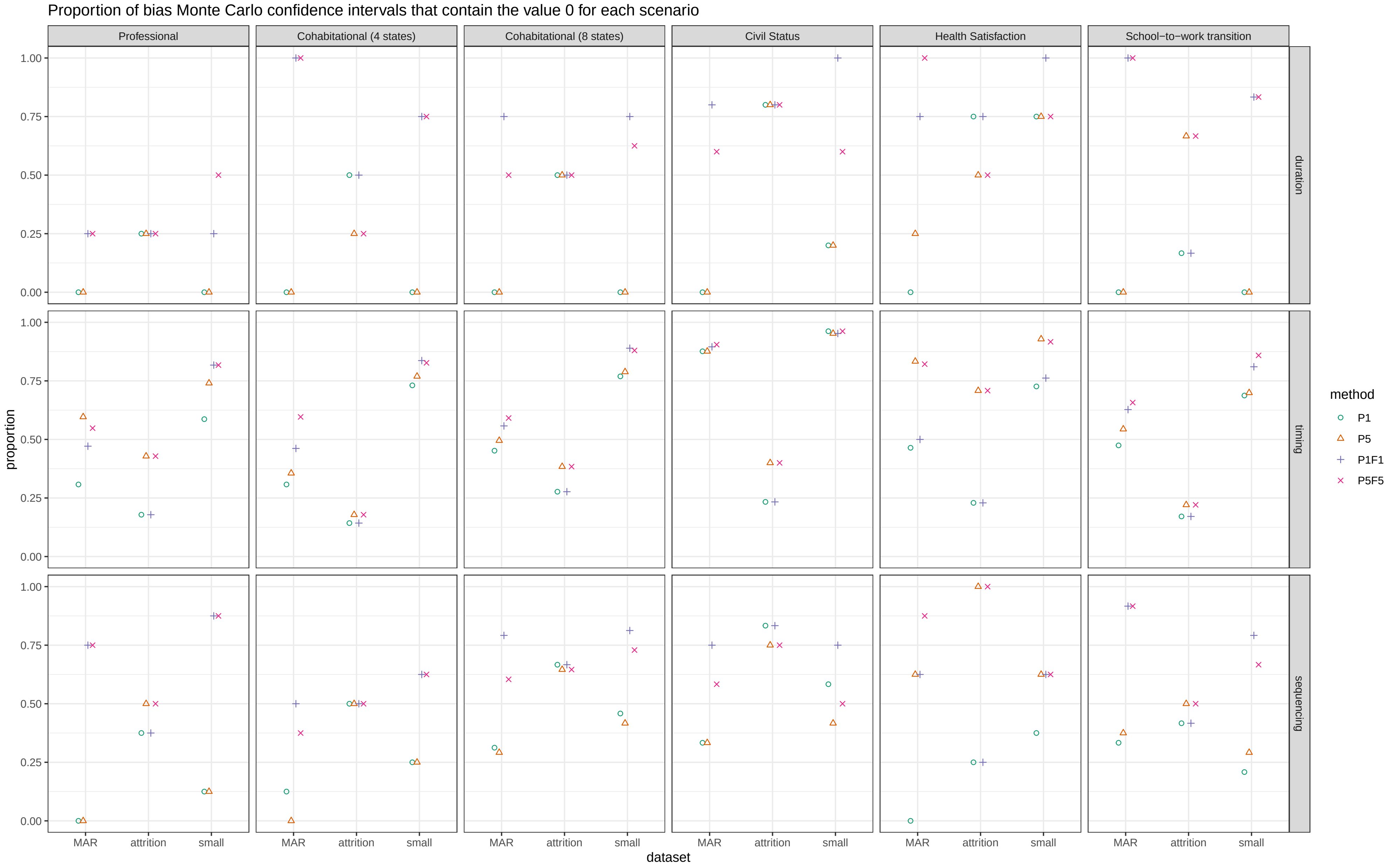

Figure A8: Comparison of the proportion of Monte Carlo confidence intervals that contains the 0 bias between the different configurations of the *MICT* multinomial algorithm. Each panel displays the results of a dataset and a criterion. Within each panel, the results are separated according to the three missing data generation processes.

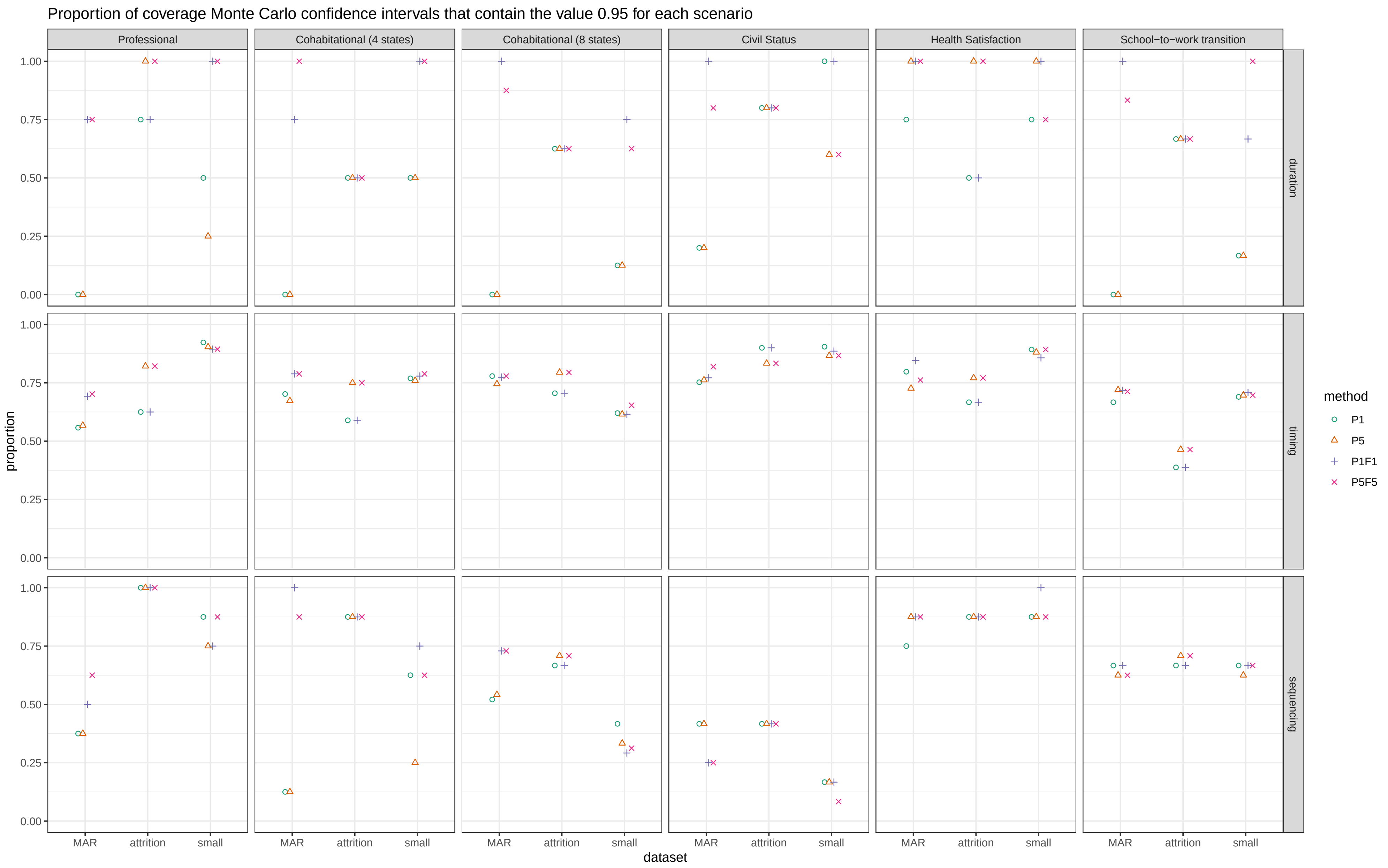

Figure A9: Comparison of the proportion of Monte Carlo confidence intervals that contains the 0.95 coverage between the different configurations of the *MICT* multinomial algorithm. Each panel displays the results of a dataset and a criterion. Within each panel, the results are separated according to the three missing data generation processes.

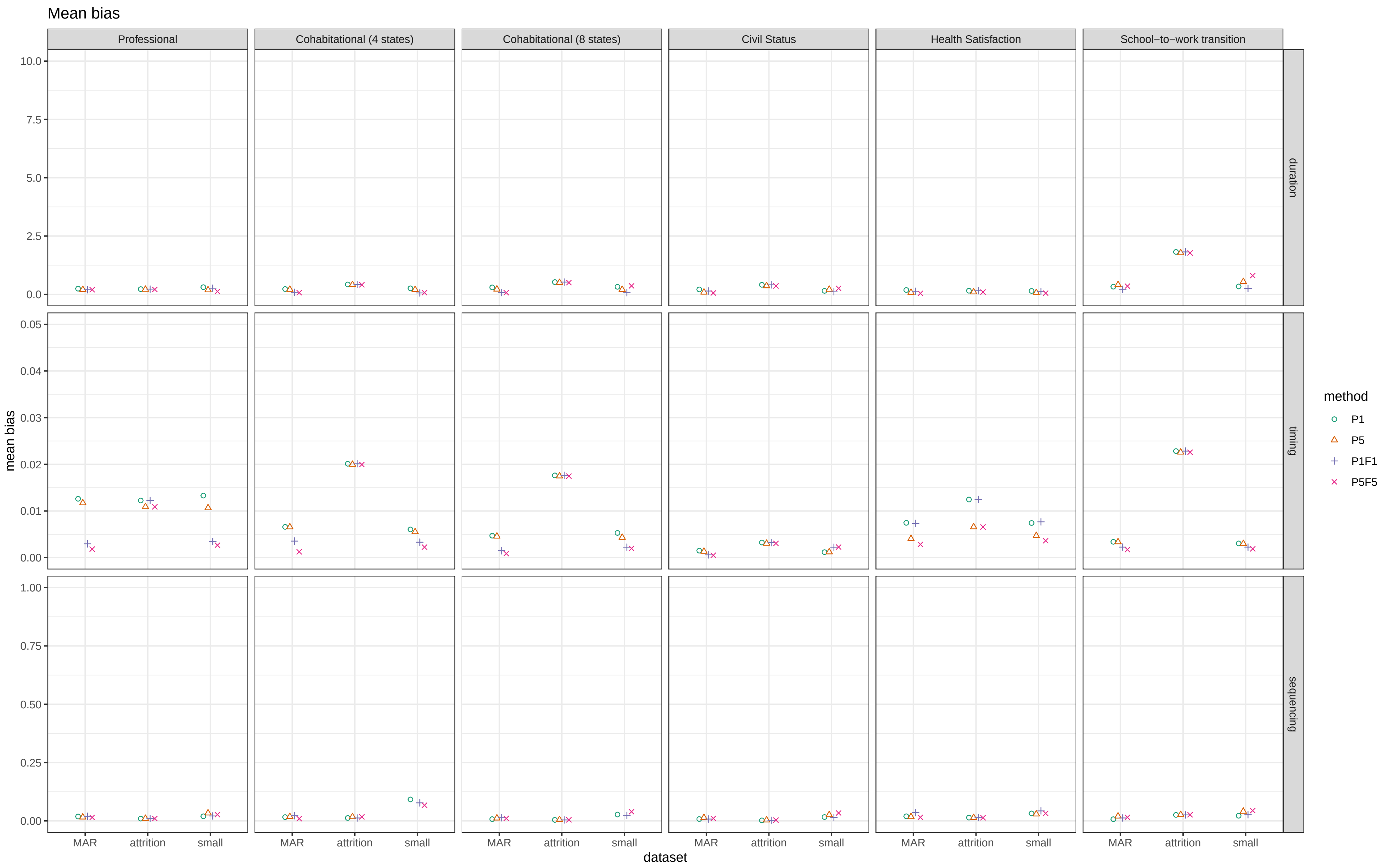

Figure A10: Comparison of the mean absolute bias between the different configurations of the *MICT* random forest algorithm. Each panel displays the results of a dataset and a criterion. Within each panel, the results are separated according to the three missing data generation processes.

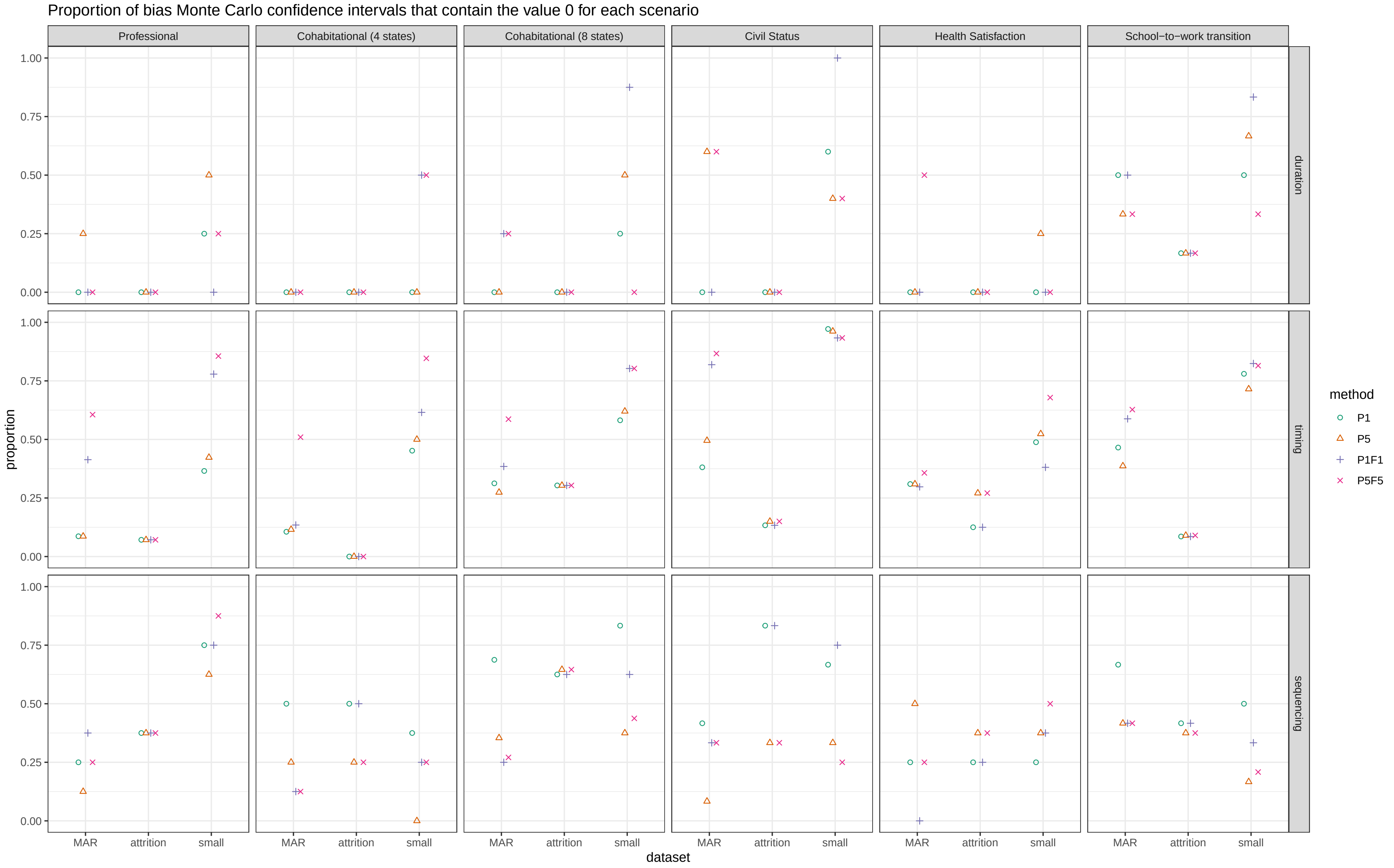

Figure A11: Comparison of the proportion of Monte Carlo confidence intervals that contains the 0 bias between the different configurations of the *MICT* random forest algorithm. Each panel displays the results of a dataset and a criterion. Within each panel, the results are separated according to the three missing data generation processes.

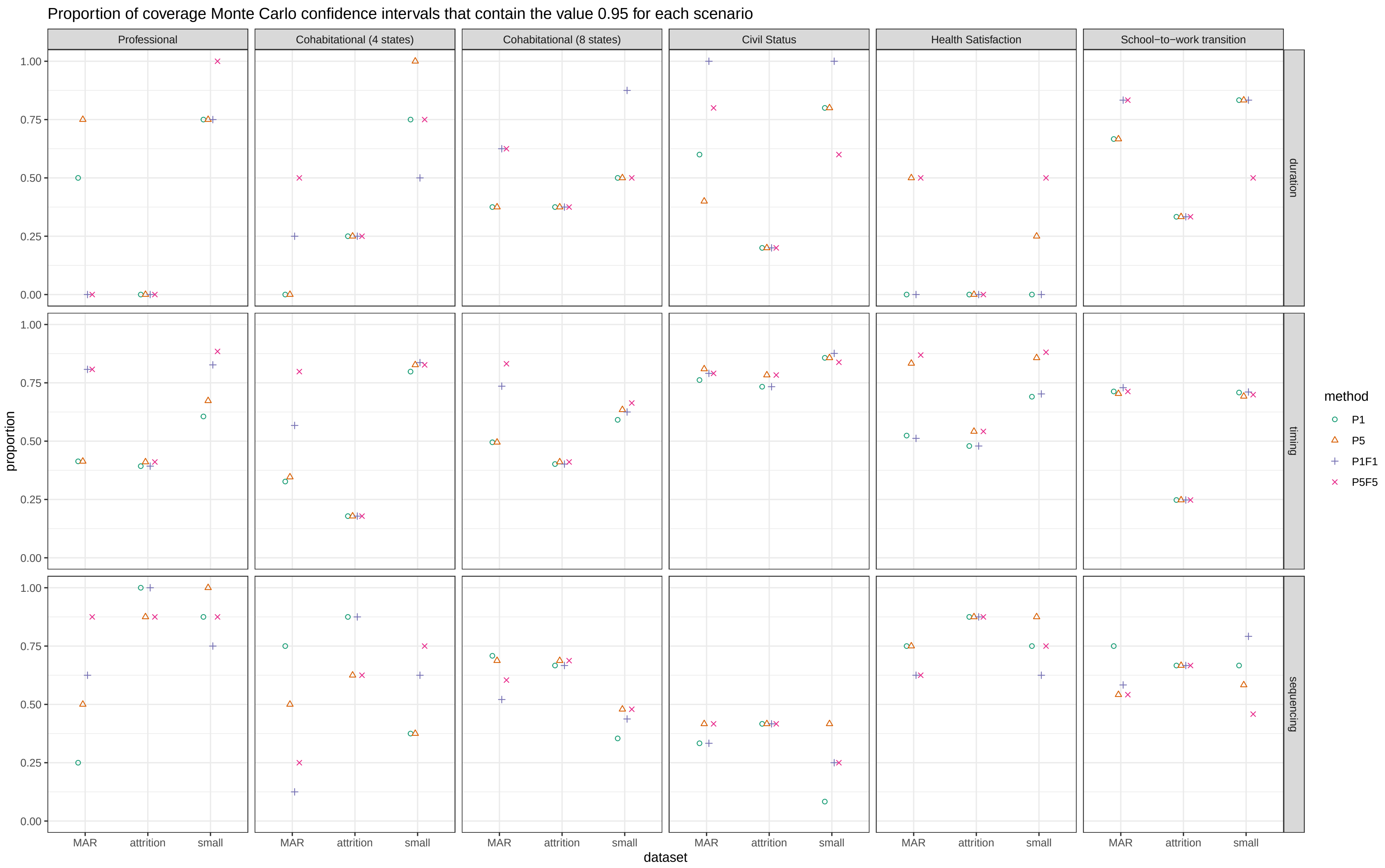

Figure A12: Comparison of the proportion of Monte Carlo confidence intervals that contains the 0.95 coverage between the different configurations of the *MICT* random forest algorithm. Each panel displays the results of a dataset and a criterion. Within each panel, the results are separated according to the three missing data generation processes.

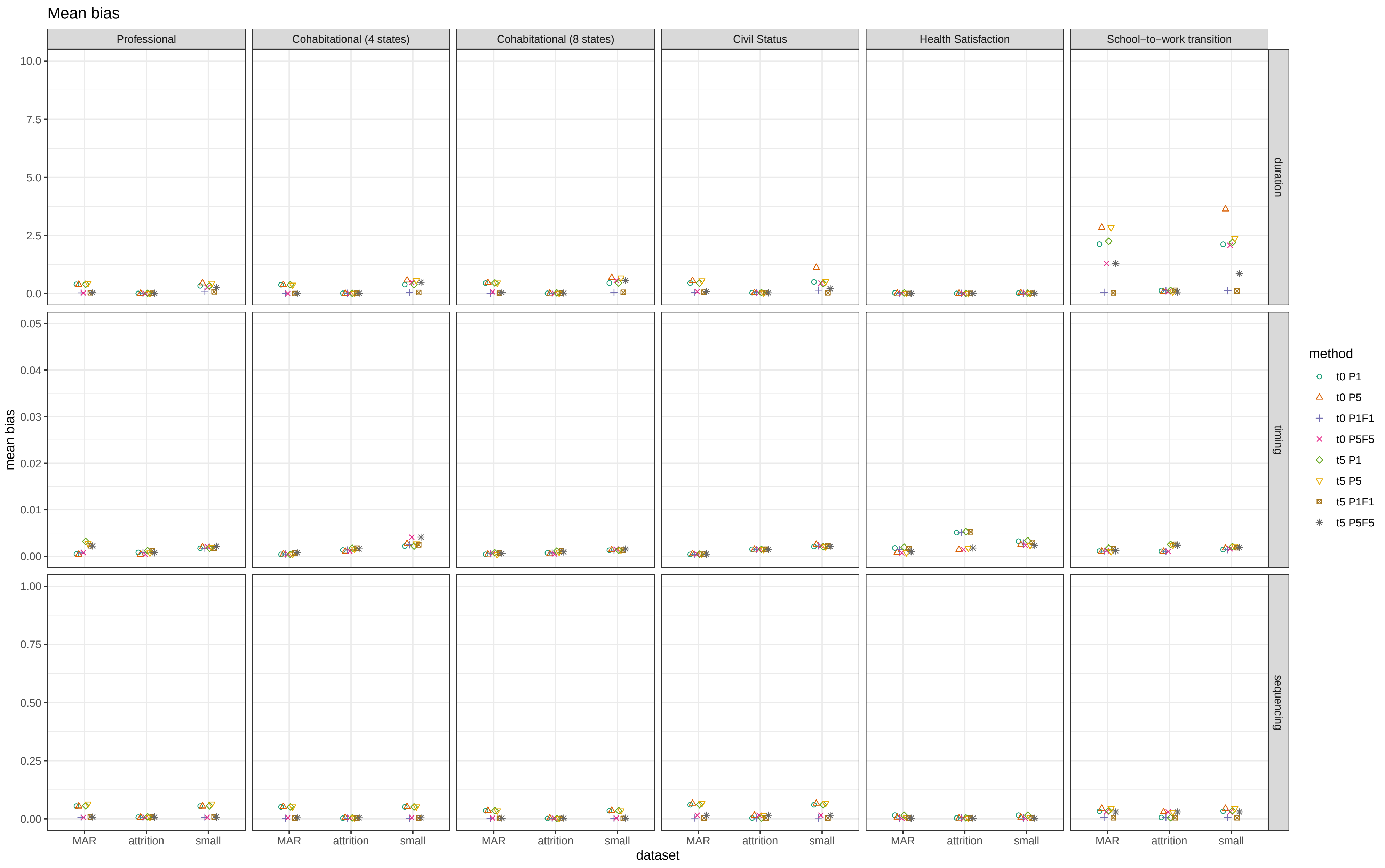

Figure A13: Comparison of the mean absolute bias between the different configurations of the *MICT-timing* multinomial algorithm. Each panel displays the results of a dataset and a criterion. Within each panel, the results are separated according to the three missing data generation processes.

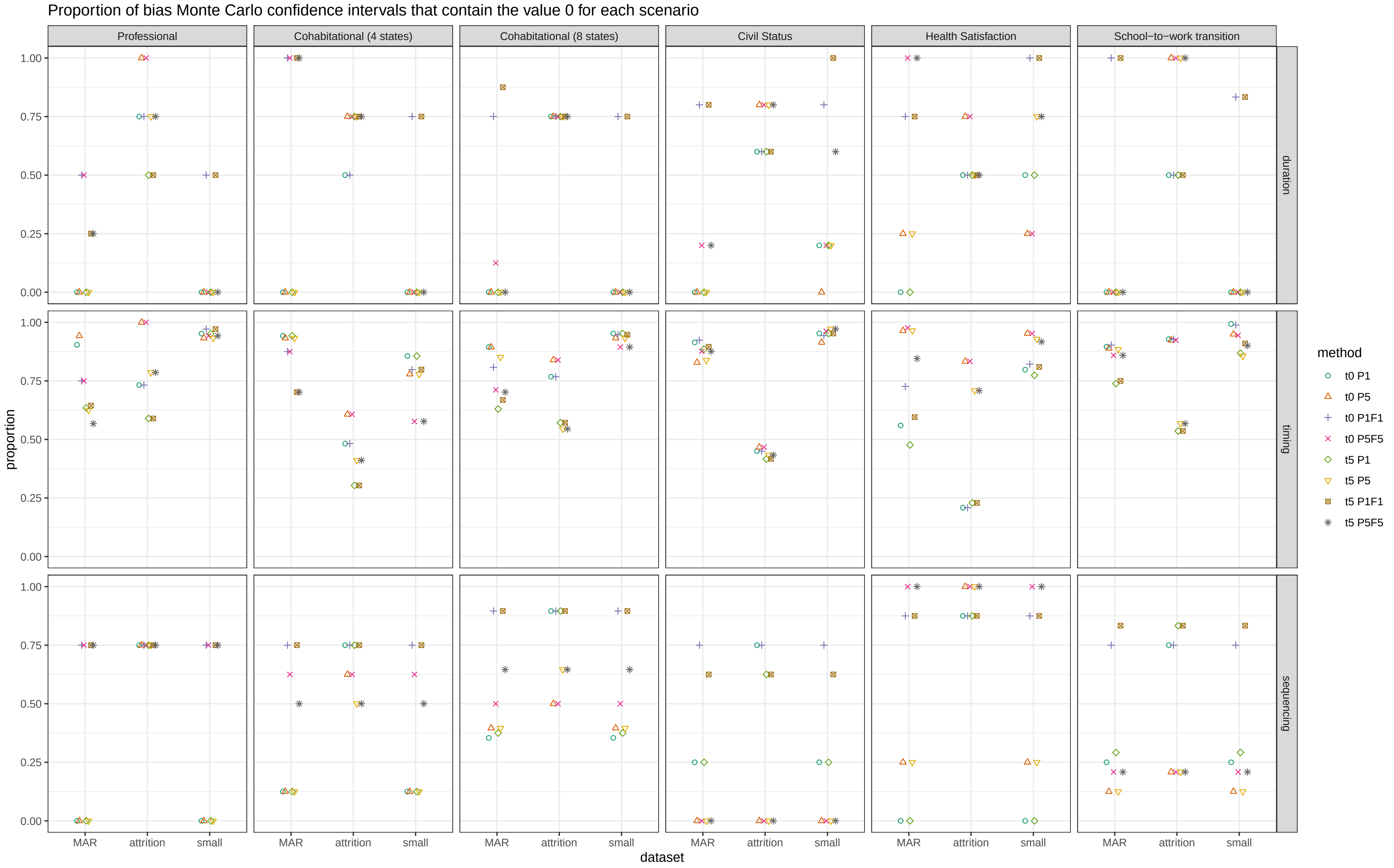

Figure A14: Comparison of the proportion of Monte Carlo confidence intervals that contains the 0 bias between the different configurations of the *MICT-timing* multinomial algorithm. Each panel displays the results of a dataset and a criterion. Within each panel, the results are separated according to the three missing data generation processes.

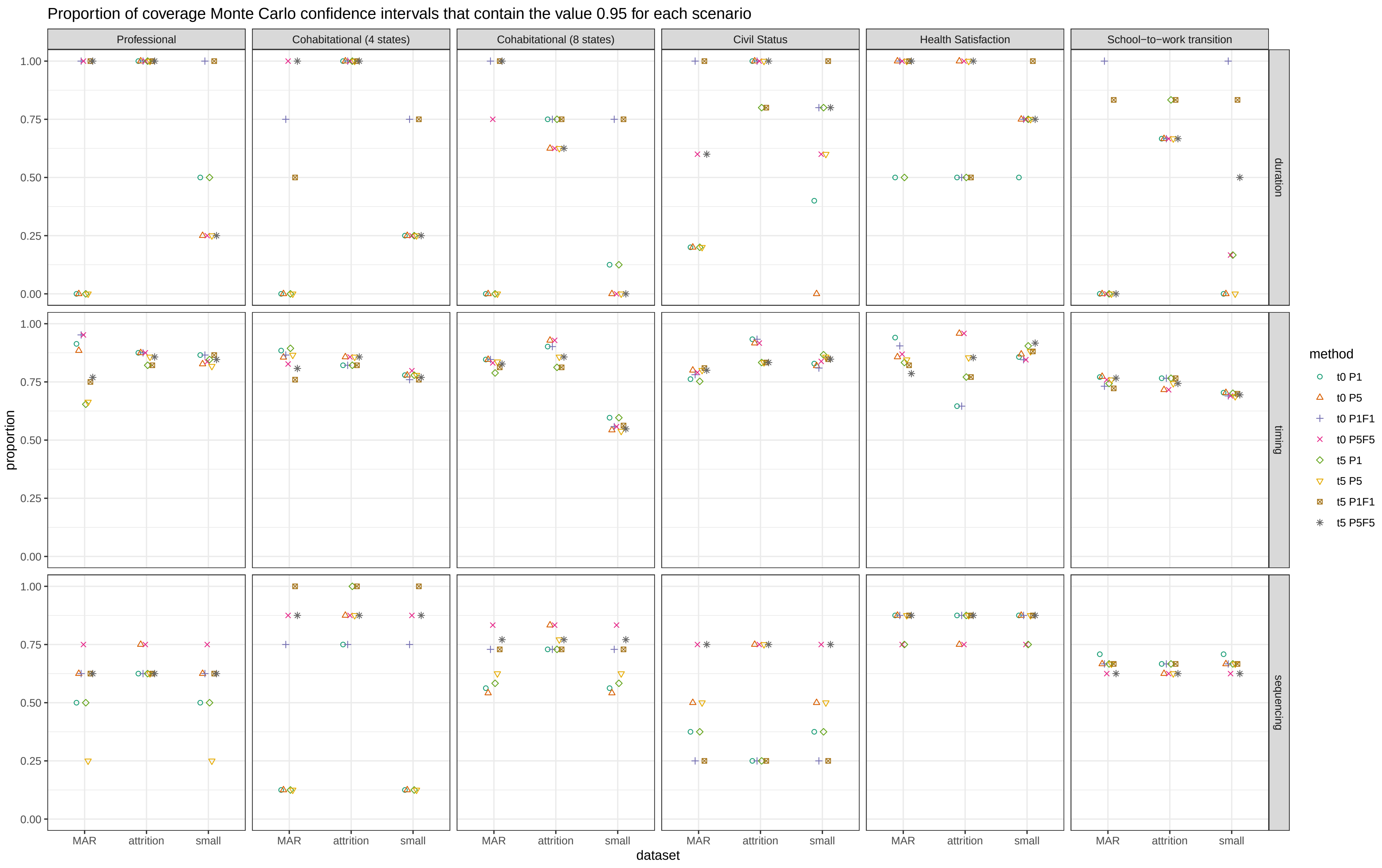

Figure A15: Comparison of the proportion of Monte Carlo confidence intervals that contains the 0.95 coverage between the different configurations of the *MICT-timing* multinomial algorithm. Each panel displays the results of a dataset and a criterion. Within each panel, the results are separated according to the three missing data generation processes.

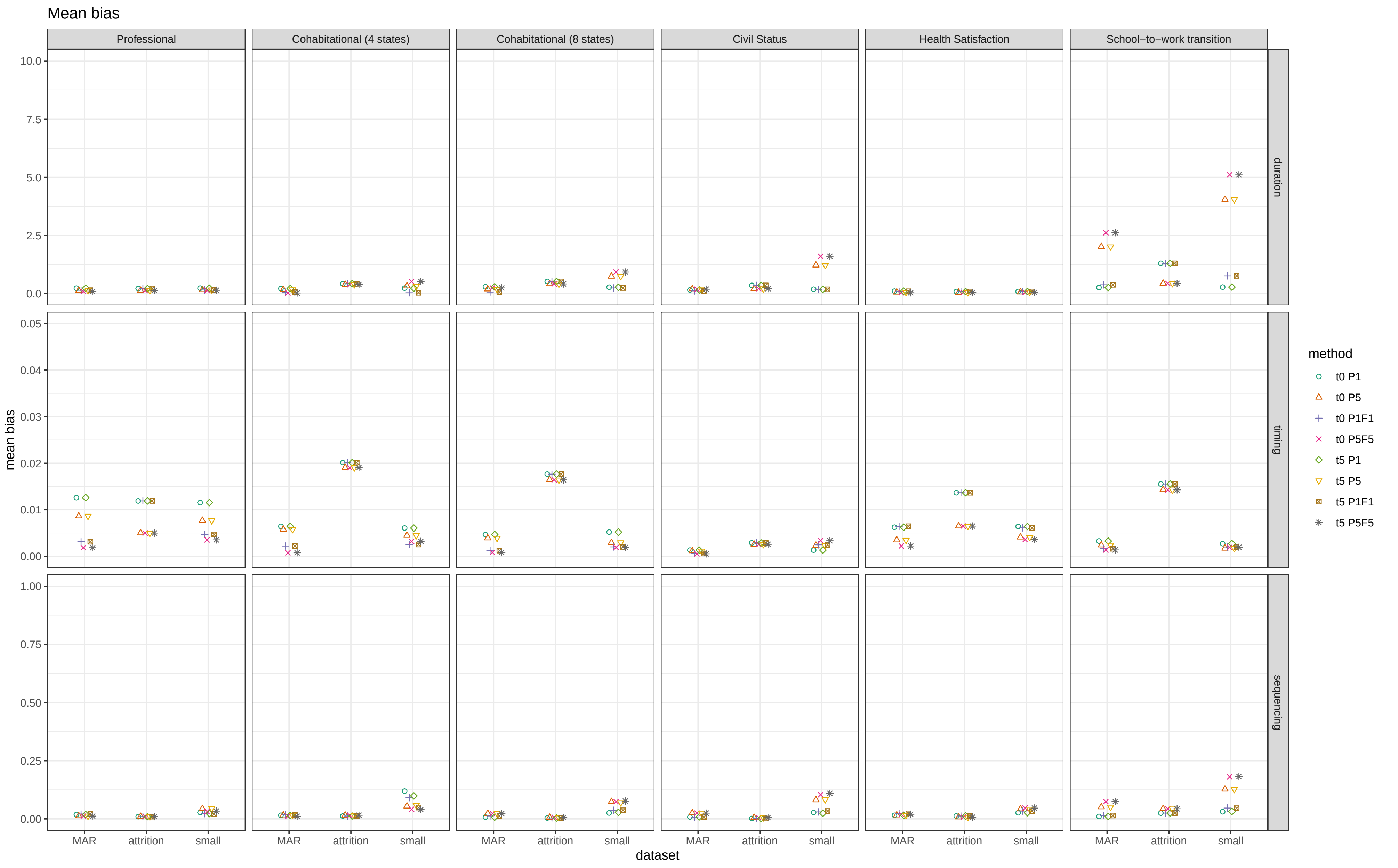

Figure A16: Comparison of the mean absolute bias between the different configurations of the *MICT-timing* random forest algorithm. Each panel displays the results of a dataset and a criterion. Within each panel, the results are separated according to the three missing data generation processes.

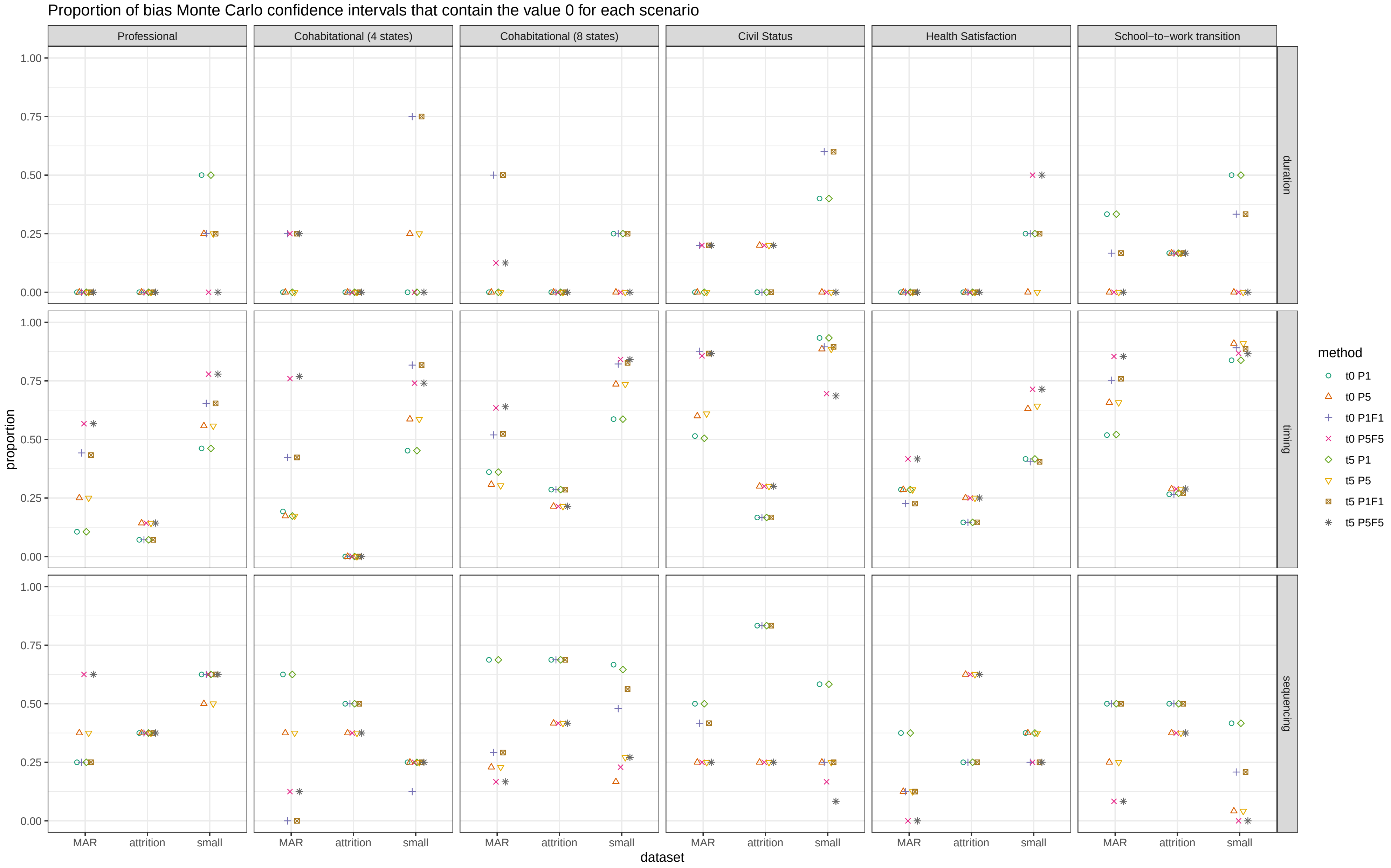

Figure A17: Comparison of the proportion of Monte Carlo confidence intervals that contains the 0 bias between the different configurations of the *MICT-timing* random forest algorithm. Each panel displays the results of a dataset and a criterion. Within each panel, the results are separated according to the three missing data generation processes.

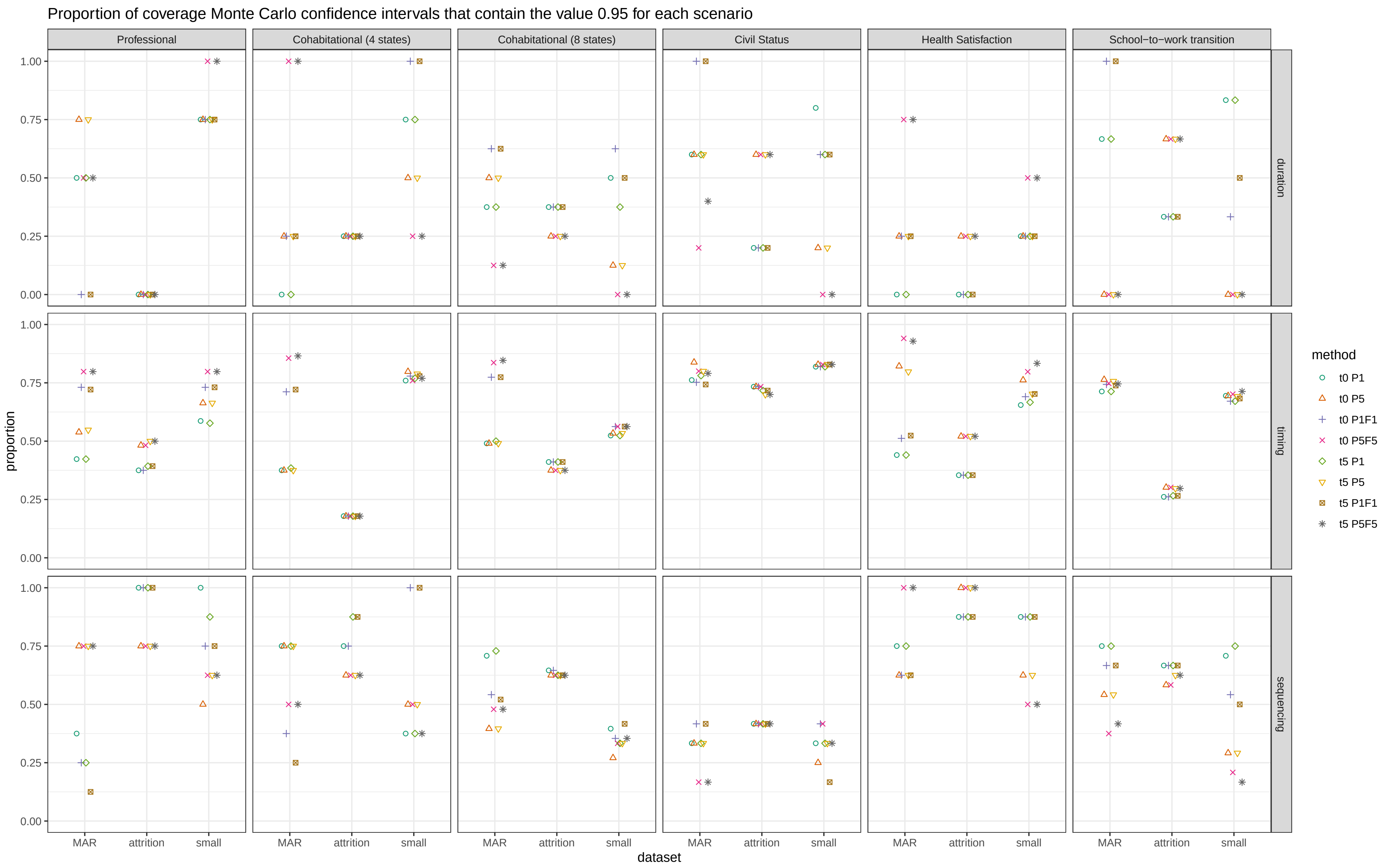

Figure A18: Comparison of the proportion of Monte Carlo confidence intervals that contains the 0.95 coverage between the different configurations of the *MICT-timing* random forest algorithm. Each panel displays the results of a dataset and a criterion. Within each panel, the results are separated according to the three missing data generation processes.

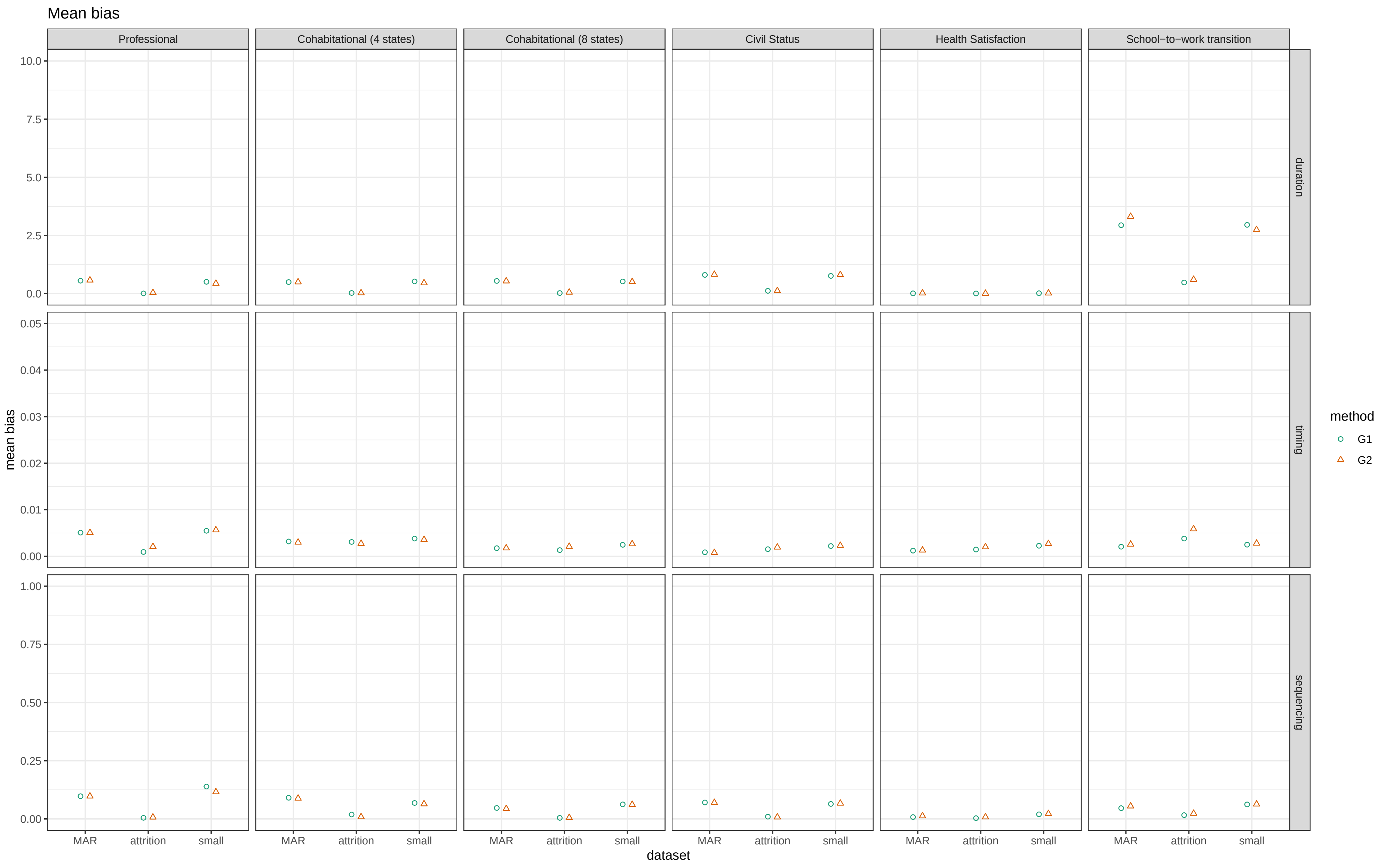

Figure A19: Comparison of the mean absolute bias between the different configurations of the vlmc algorithm. Each panel displays the results of a dataset and a criterion. Within each panel, the results are separated according to the three missing data generation processes.

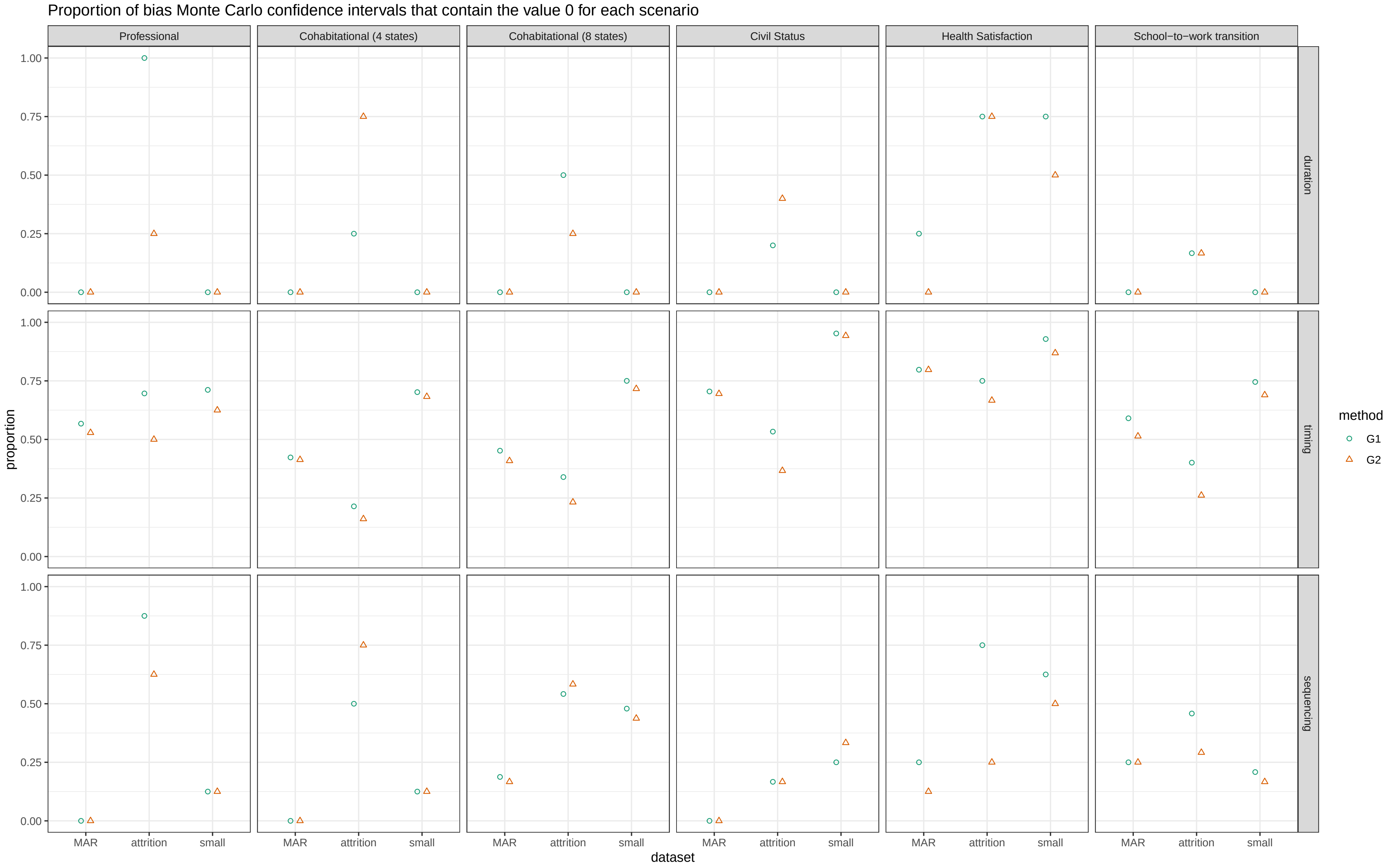

Figure A20: Comparison of the proportion of Monte Carlo confidence intervals that contains the 0 bias between the different configurations of the vlmc algorithm. Each panel displays the results of a dataset and a criterion. Within each panel, the results are separated according to the three missing data generation processes.

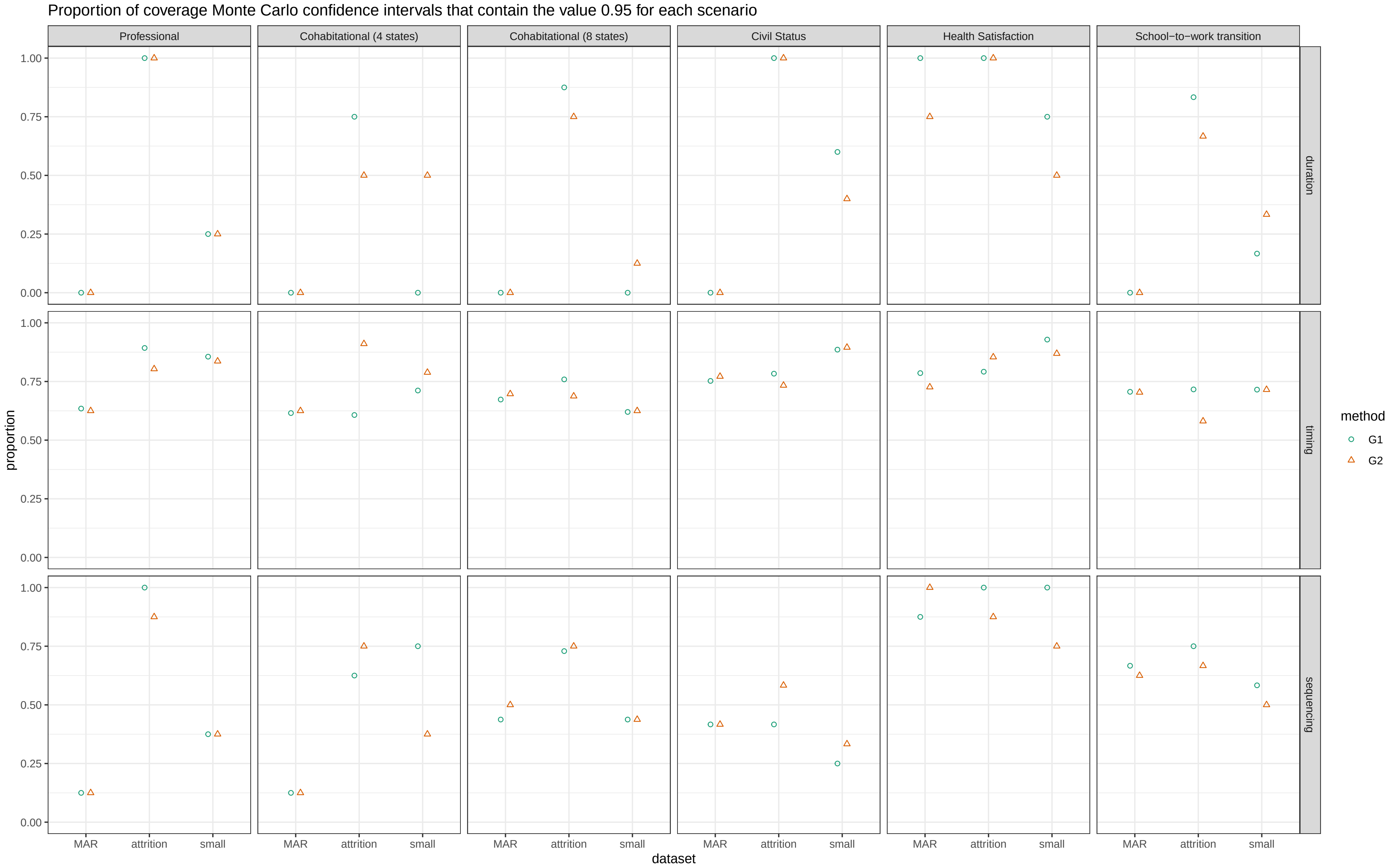

Figure A21: Comparison of the proportion of Monte Carlo confidence intervals that contains the 0.95 coverage between the different configurations of the vlmc algorithm. Each panel displays the results of a dataset and a criterion. Within each panel, the results are separated according to the three missing data generation processes.

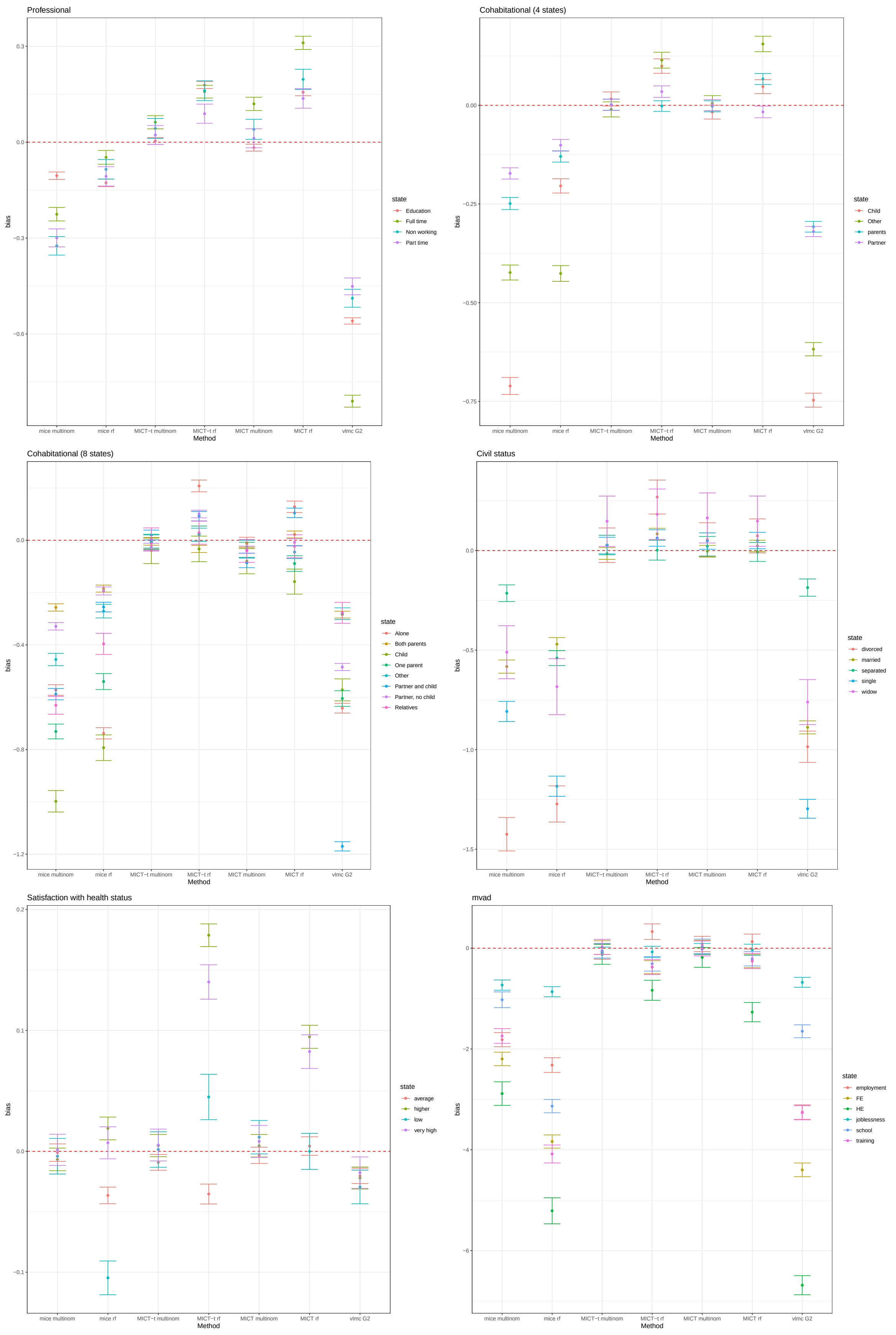

Figure A22: Results regarding the bias of the duration parameters in the case of an MAR missing data. Each panel displays the results of a dataset. The x-axis depicts the different imputation methods. The estimated bias and its 95% Monte Carlo confidence intervals are provided for the mean time spent in each state.

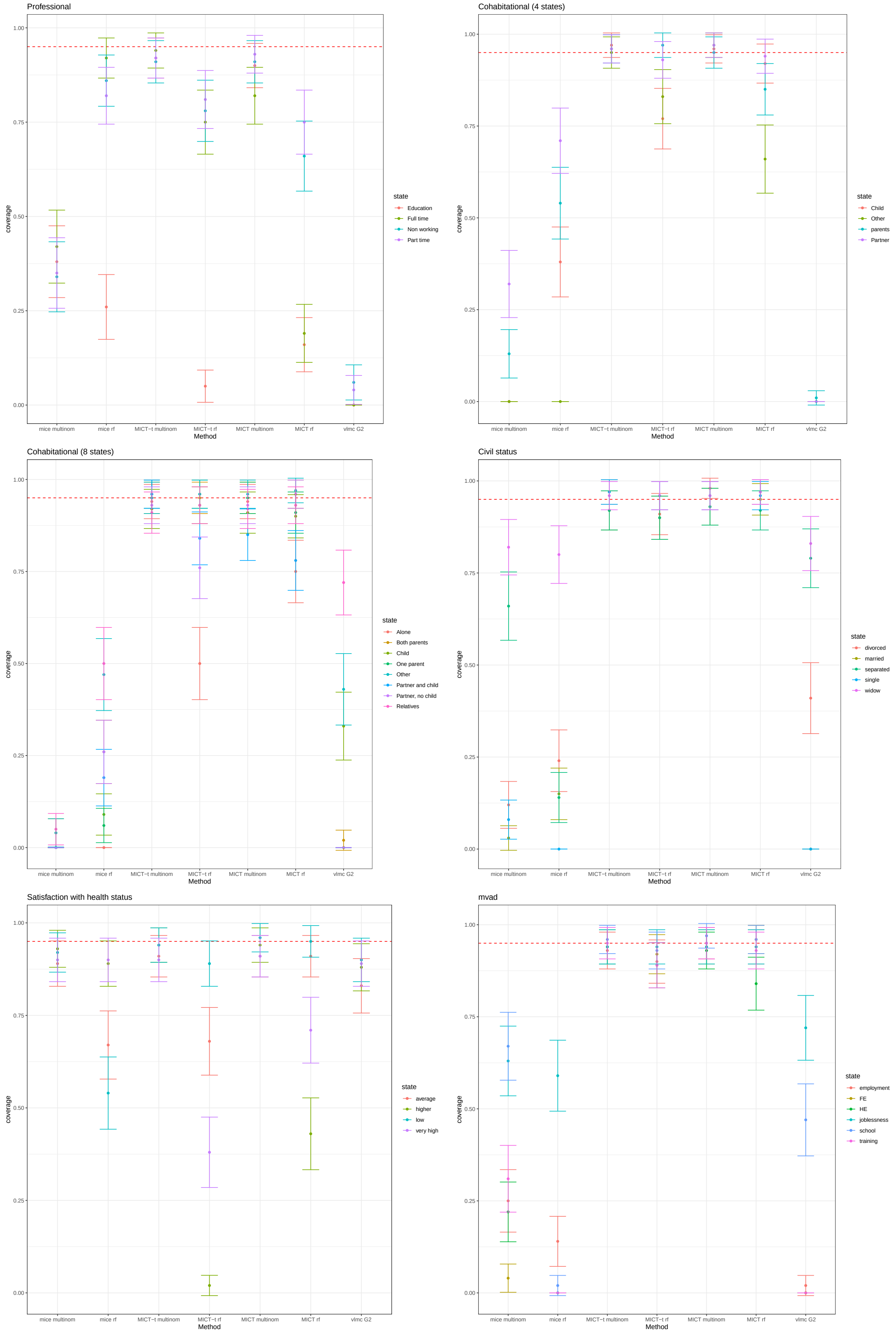

Figure A23: Results regarding the coverage of the duration parameters in the case of an MAR missing data. Each panel displays the results of a dataset. The x-axis depicts the different imputation methods. The estimated bias and its 95% Monte Carlo confidence intervals are provided for the mean time spent in each state.

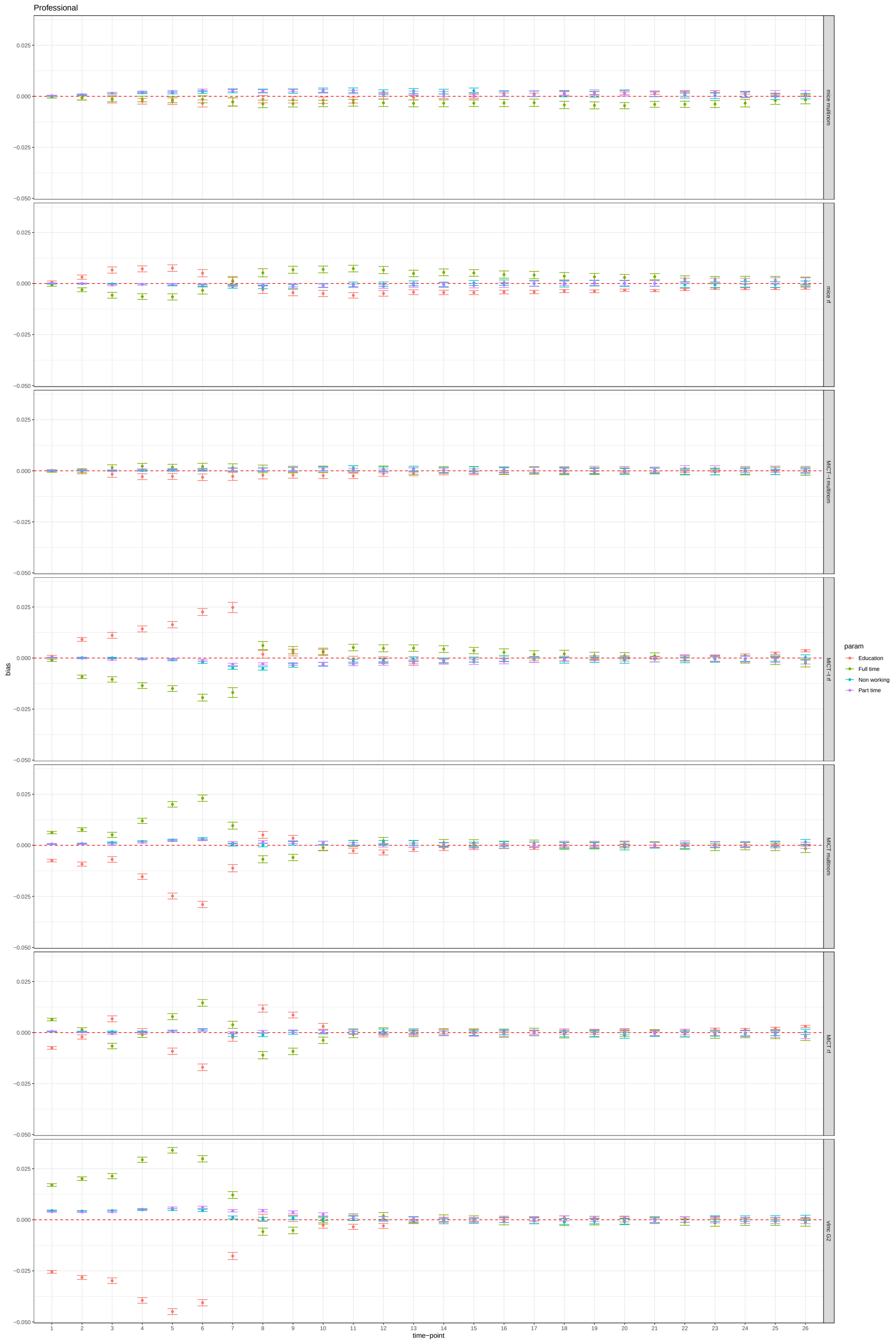

Figure A24: Results regarding the bias of the timing parameters in the case of an MAR process simulated on professional trajectories. Each panel displays the results of an imputation method. The estimated bias and its 95% Monte Carlo confidence intervals are shown for the probability of belonging to each state at each time point.

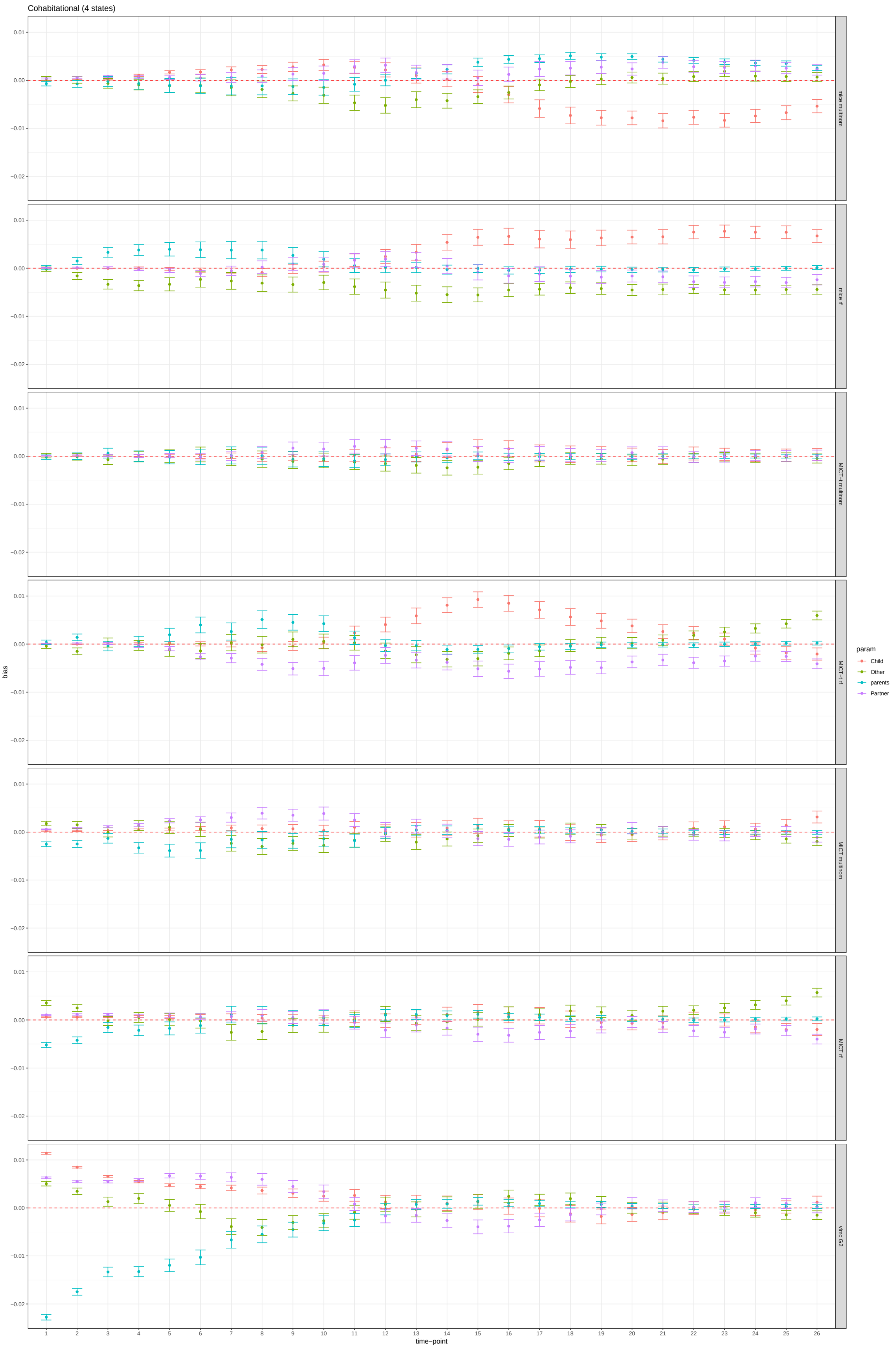

Figure A25: Results regarding the bias of the timing parameters in the case of an MAR process simulated on cohabitational status coded as four states. Each panel displays the results of an imputation method. The estimated bias and its 95% Monte Carlo confidence intervals are shown for the probability of belonging to each state at each time point.

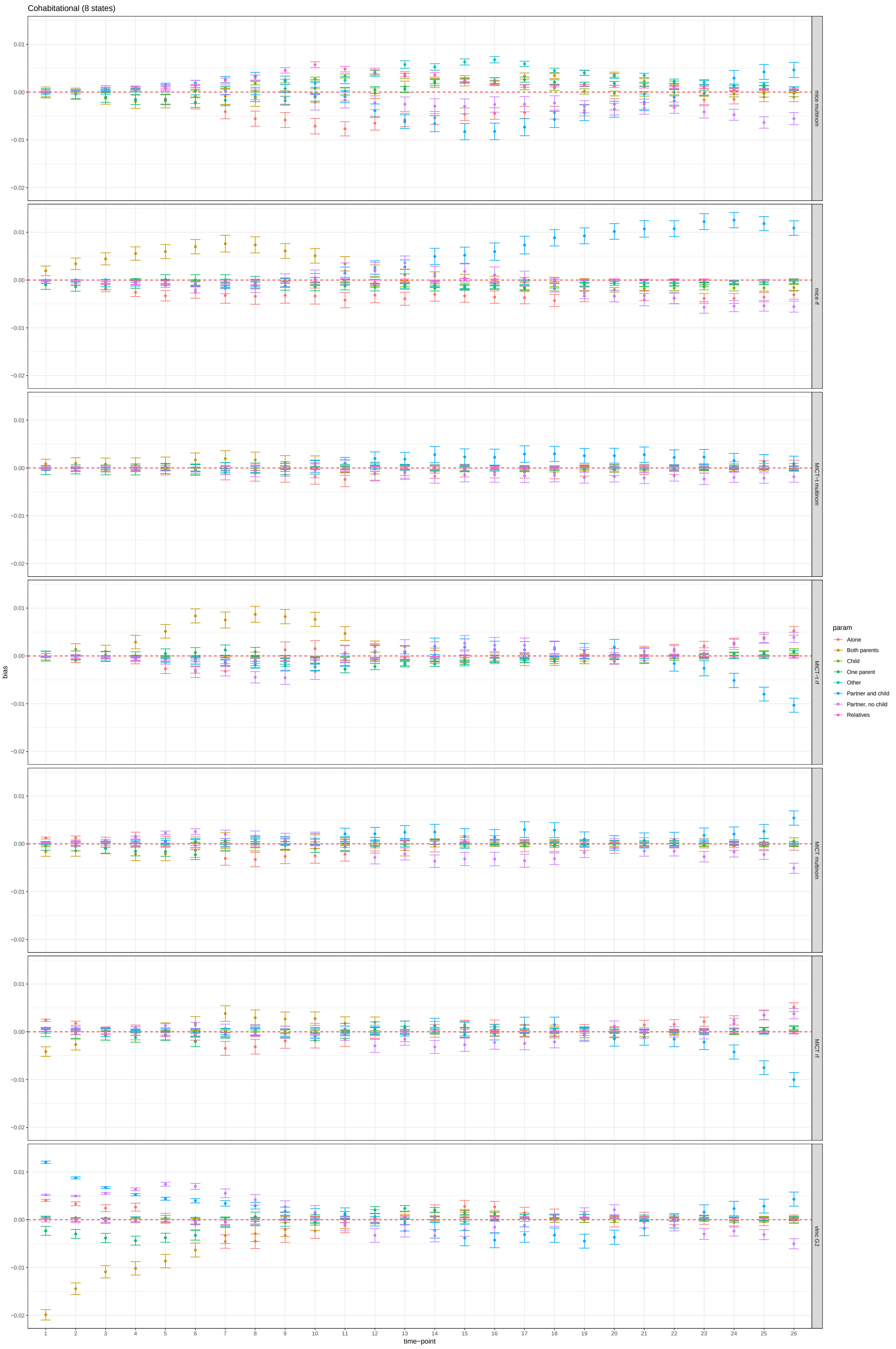

Figure A26: Results regarding the bias of the timing parameters in the case of an MAR process simulated on cohabitational status coded as eight states. Each panel displays the results of an imputation method. The estimated bias and its 95% Monte Carlo confidence intervals are shown for the probability of belonging to each state at each time point.

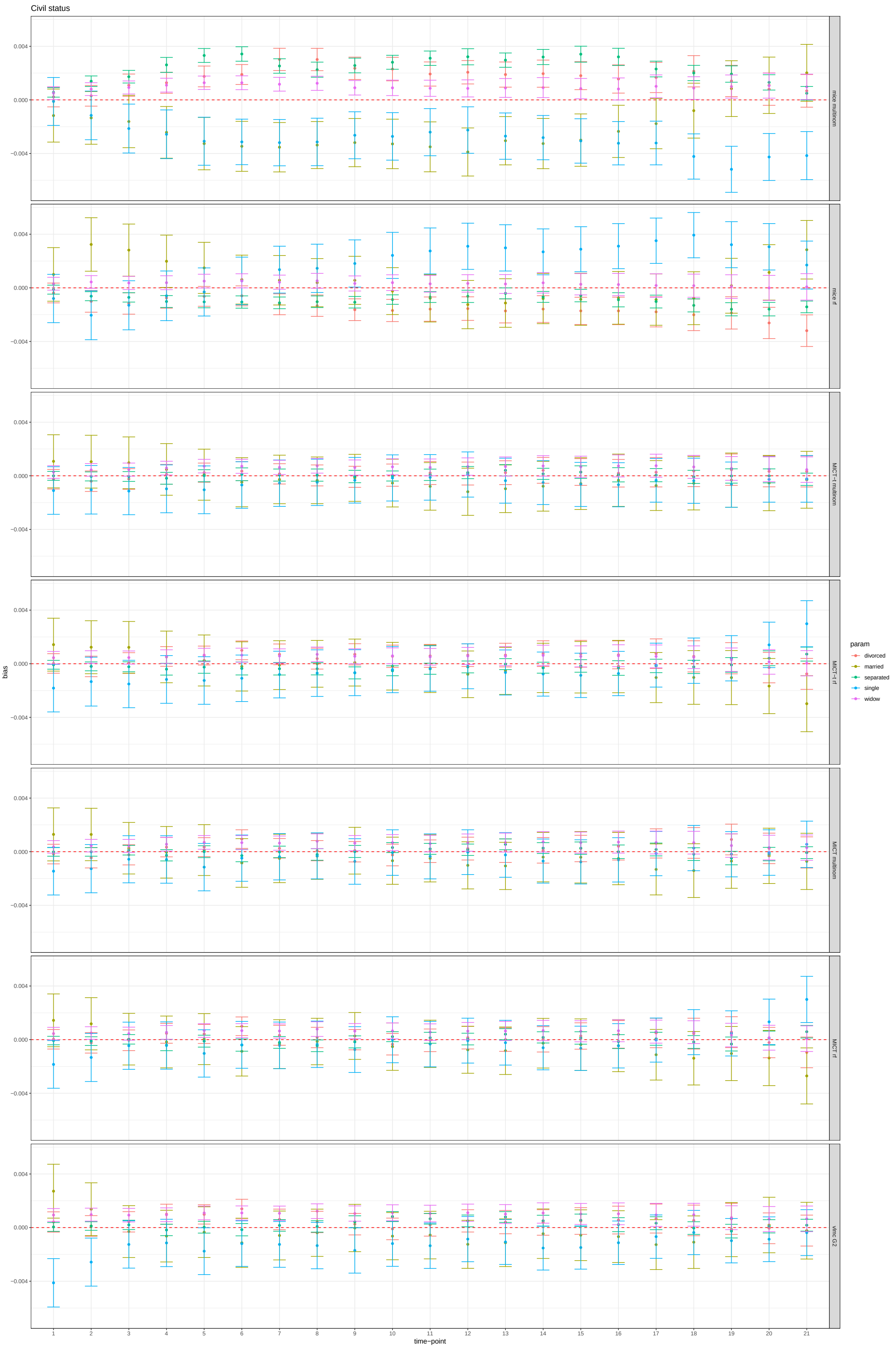

Figure A27: Results regarding the bias of the timing parameters in the case of an MAR process simulated on civil status. Each panel displays the results of an imputation method. The estimated bias and its 95% Monte Carlo confidence intervals are shown for the probability of belonging to each state at each time point.

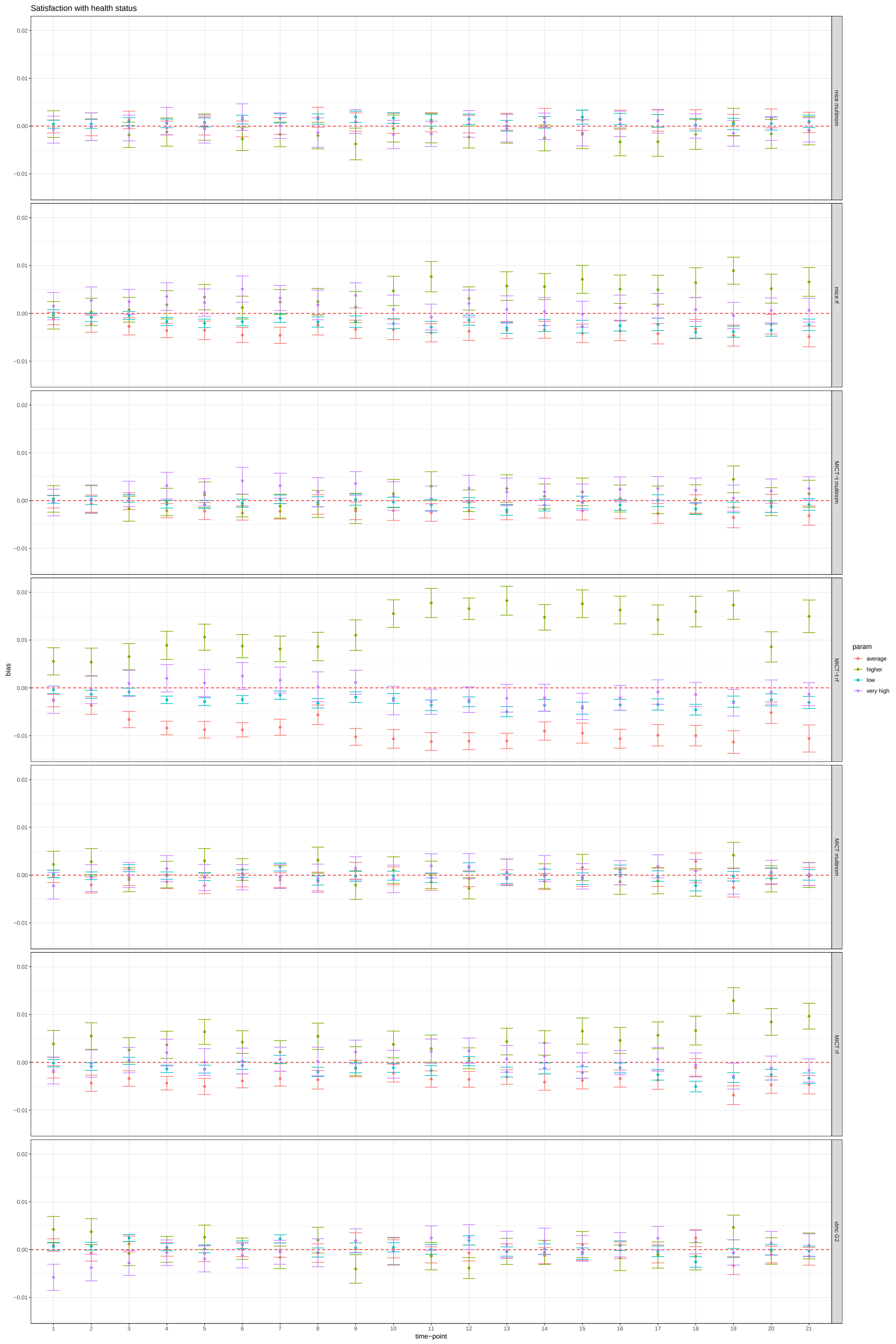

Figure A28: Results regarding the bias of the timing parameters in the case of an MAR process simulated on satisfaction with health status. Each panel displays the results of an imputation method. The estimated bias and its 95% Monte Carlo confidence intervals are shown for the probability of belonging to each state at each time point.

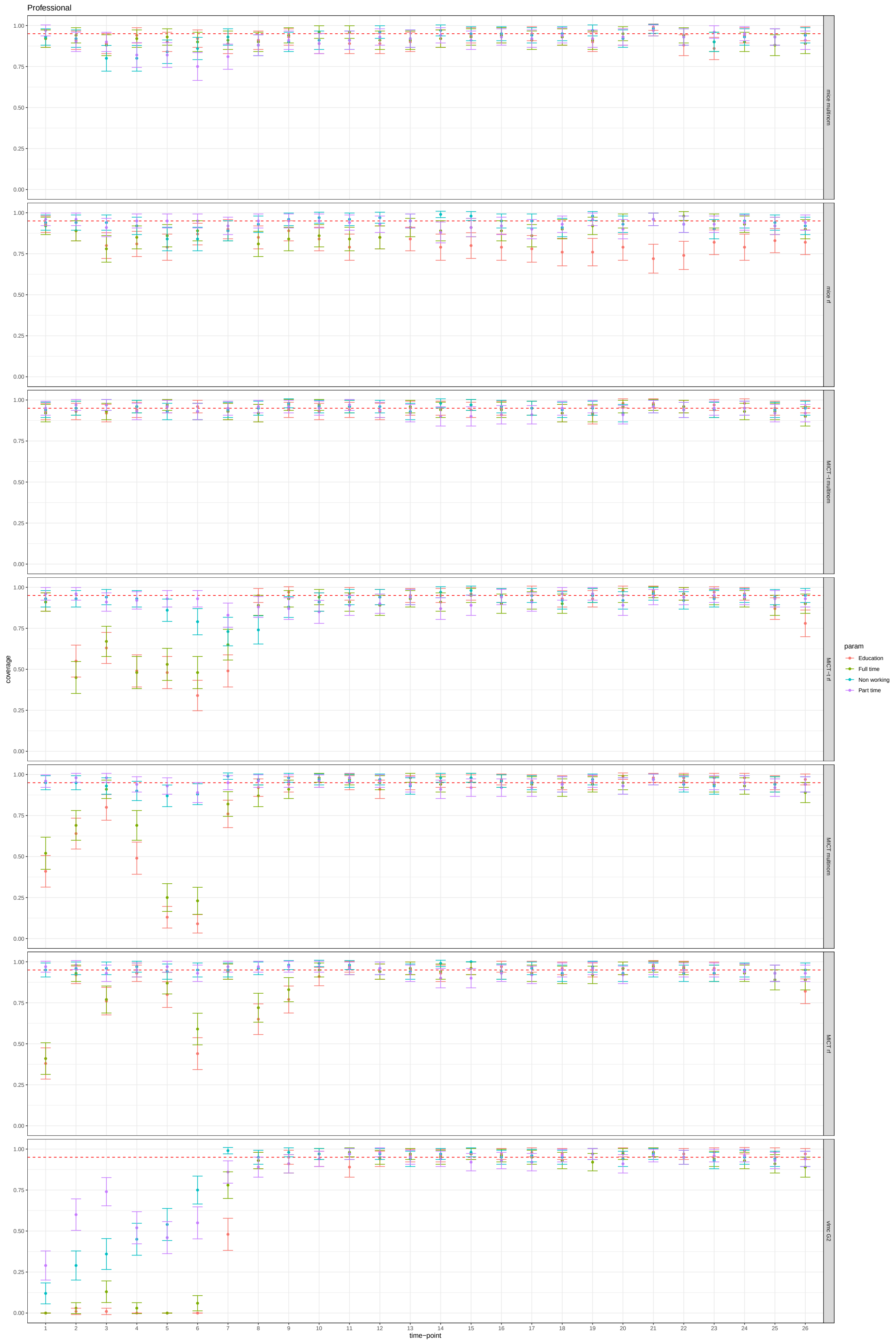

Figure A29: Results regarding the coverage of the timing parameters in the case of an MAR process simulated on professional trajectories. Each panel displays the results of an imputation method. The estimated coverage and its 95% Monte Carlo confidence intervals are shown for the probability of belonging to each state at each time point.

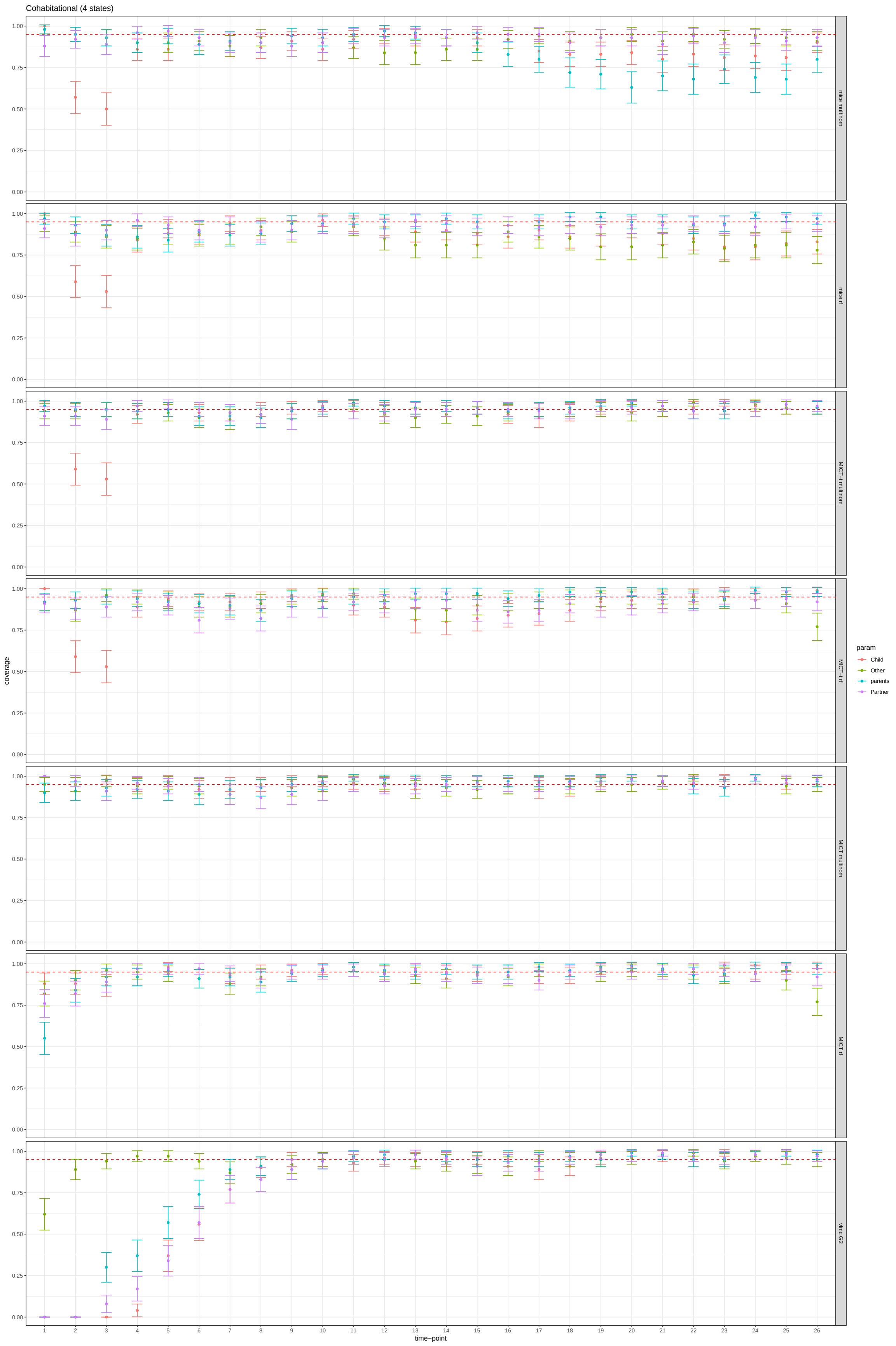

Figure A30: Results regarding the coverage of the timing parameters in the case of an MAR process simulated on cohabitational status coded as four states. Each panel displays the results of an imputation method. The estimated coverage and its 95% Monte Carlo confidence intervals are shown for the probability of belonging to each state at each time point.

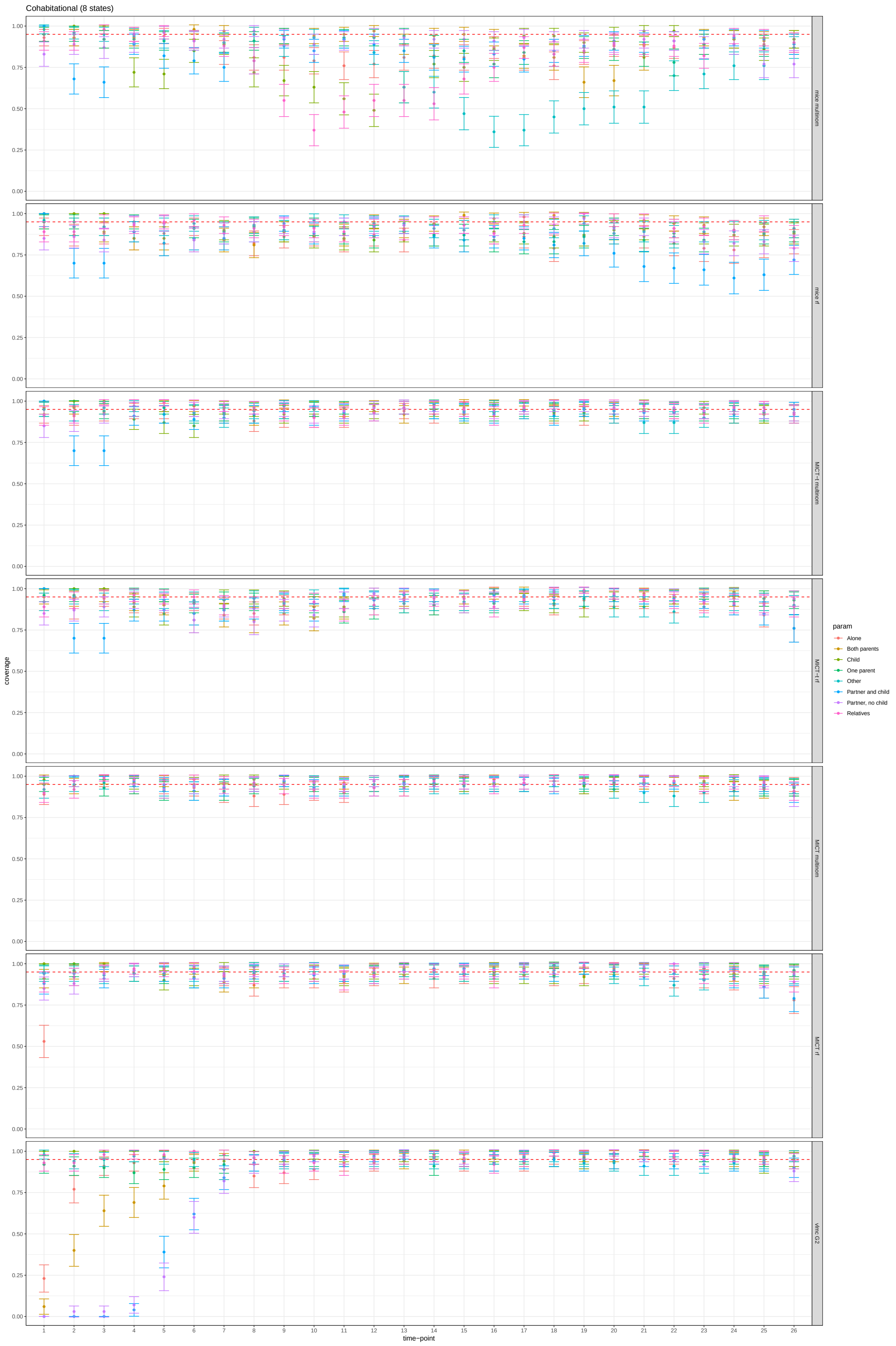

Figure A31: Results regarding the coverage of the timing parameters in the case of an MAR process simulated on cohabitational status coded as eight states. Each panel displays the results of an imputation method. The estimated coverage and its 95% Monte Carlo confidence intervals are shown for the probability of belonging to each state at each time point.

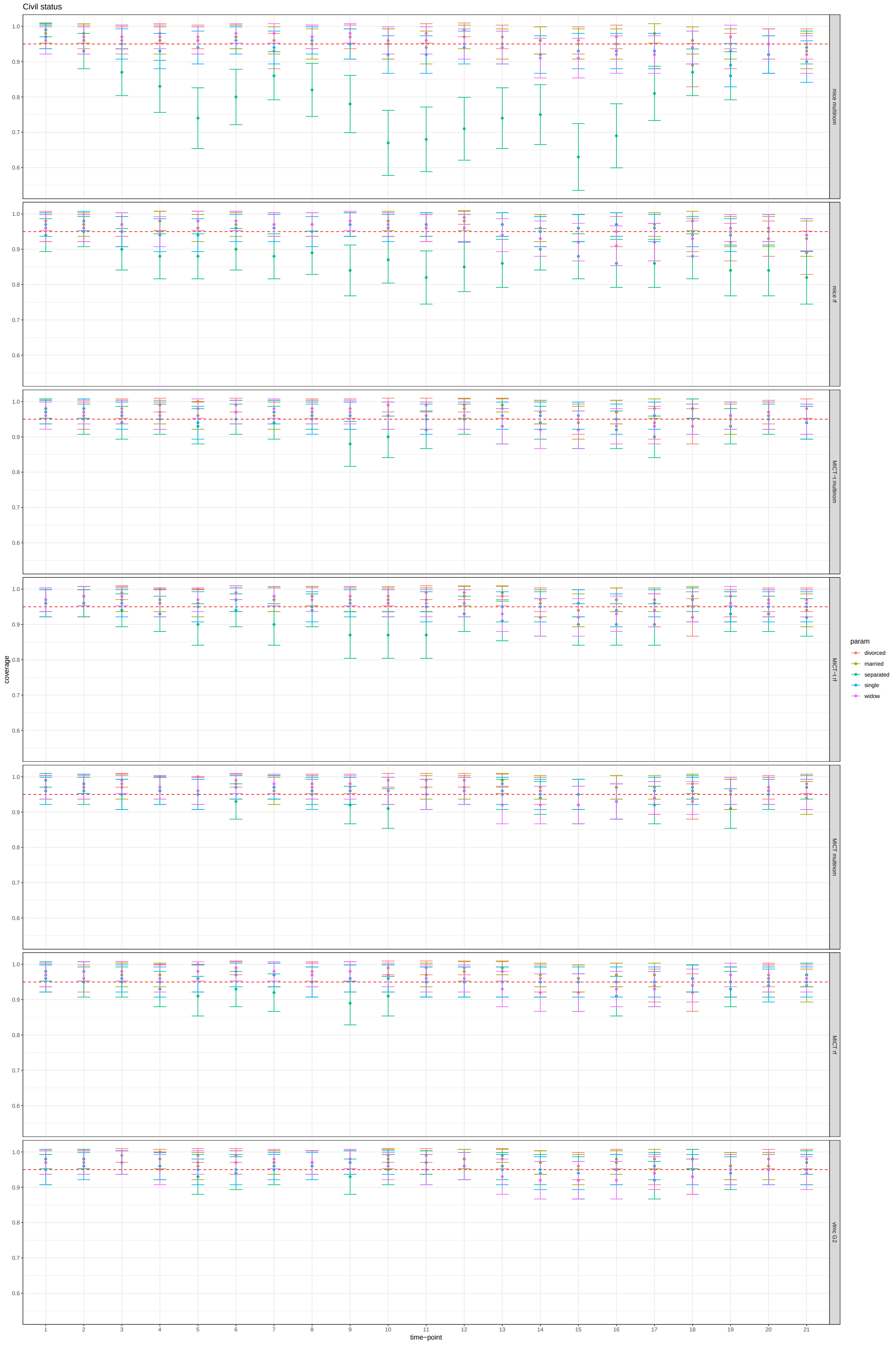

Figure A32: Results regarding the coverage of the timing parameters in the case of an MAR process simulated on civil status. Each panel displays the results of an imputation method. The estimated coverage and its 95% Monte Carlo confidence intervals are shown for the probability of belonging to each state at each time point.

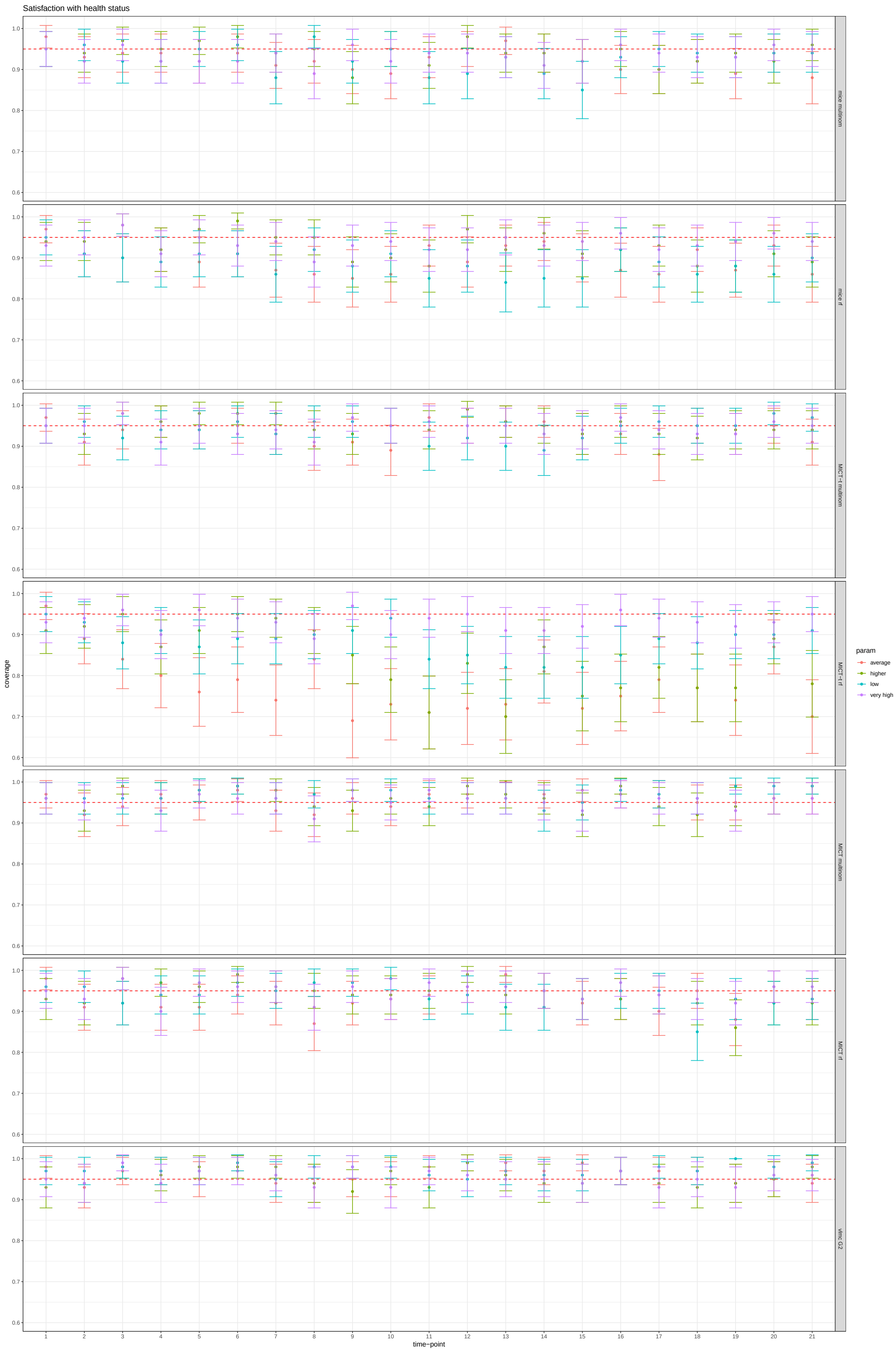

Figure A33: Results regarding the coverage of the timing parameters in the case of an MAR process simulated on satisfaction with health status. Each panel displays the results of an imputation method. The estimated coverage and its 95% Monte Carlo confidence intervals are shown for the probability of belonging to each state at each time point.

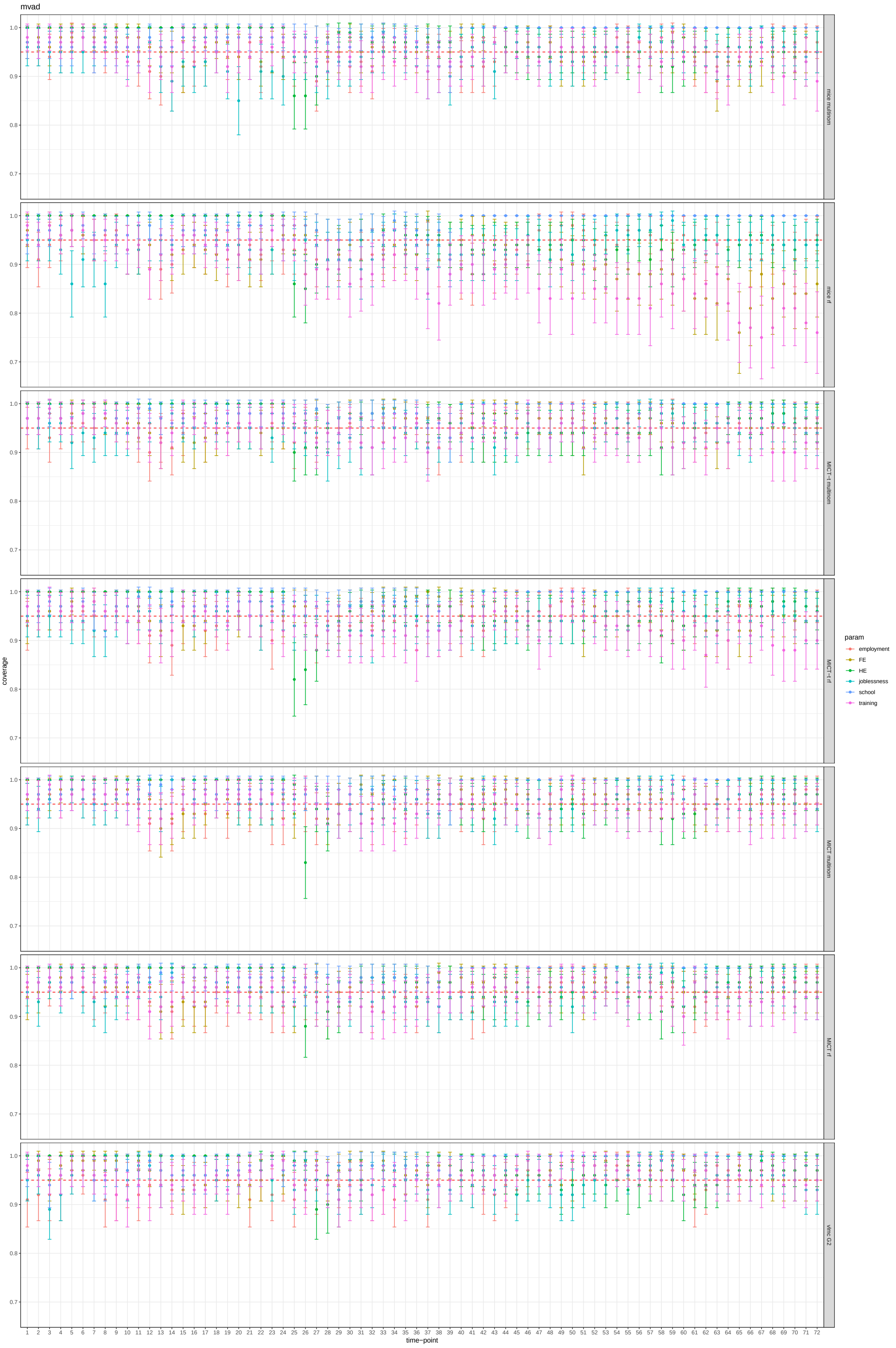

Figure A34: Results regarding the coverage of the timing parameters in the case of an MAR process simulated on school-to-work transitions. Each panel displays the results of an imputation method. The estimated coverage and its 95% Monte Carlo confidence intervals are shown for the probability of belonging to each state at each time point.

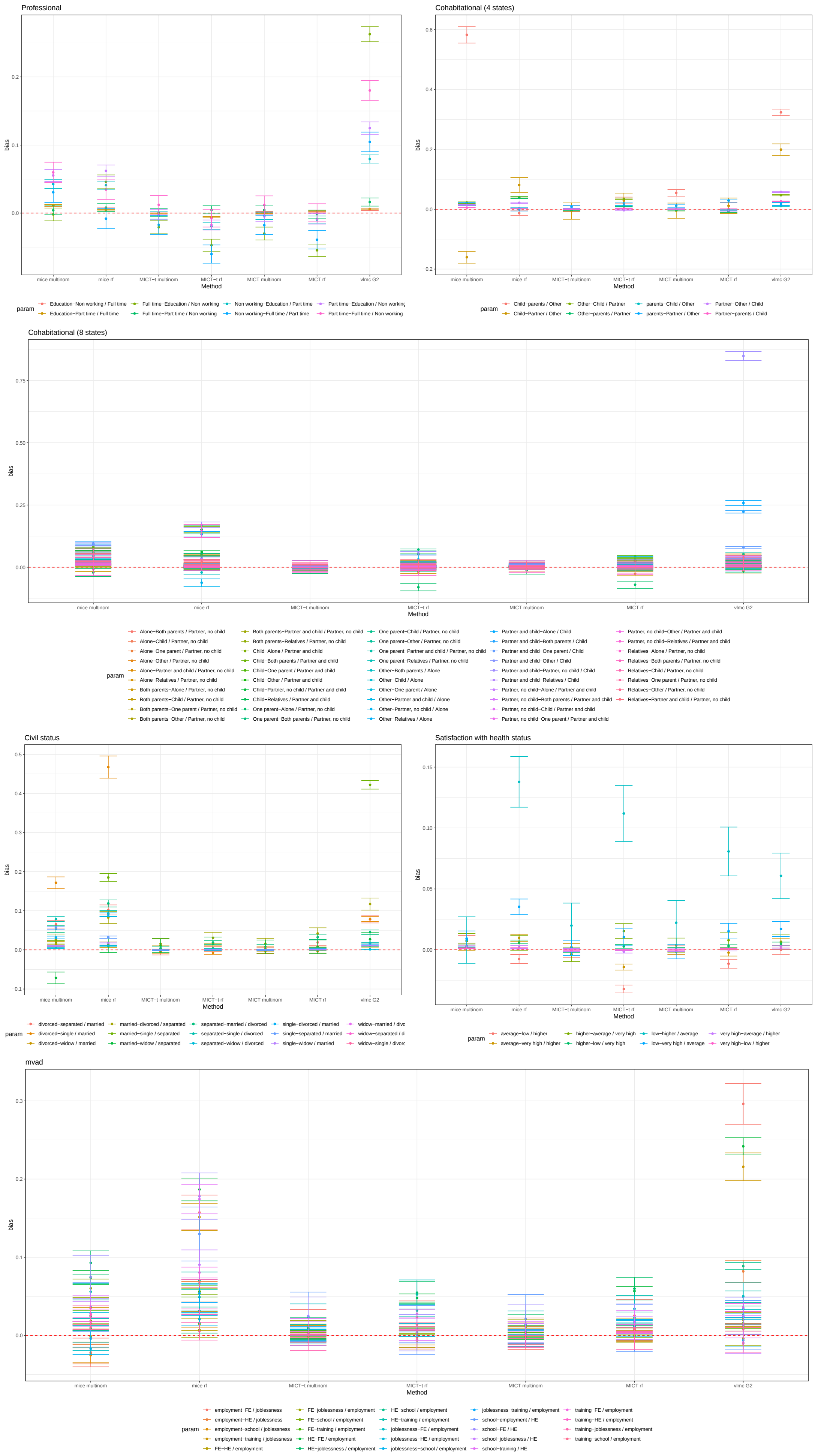

Figure A35: Results regarding the bias of the sequencing parameters in the case of an MAR process. Each panel displays the results of a dataset. The x-axis depicts the different imputation methods. The x-axis depicts the different imputation methods. The estimated bias and its 95% Monte Carlo confidence intervals are shown for each relative risk. They are labeled in the format "state A - state B / state C," representing the relative risk of transitioning to state B versus transitioning to state C when in state A.

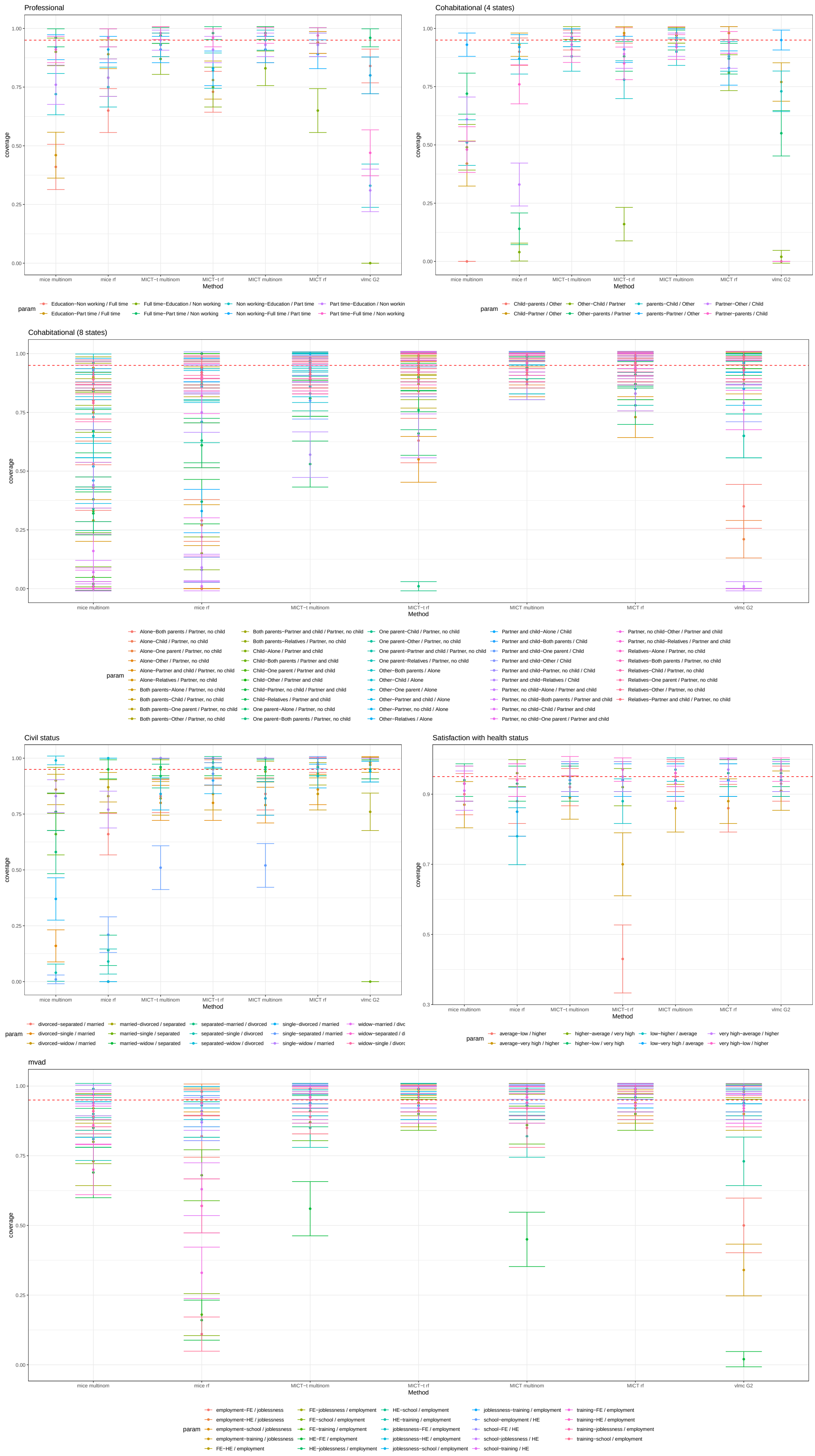

Figure A36: Results regarding the coverage of the sequencing parameters in the case of an MAR process. Each panel displays the results of a dataset. The x-axis depicts the different imputation methods. The x-axis depicts the different imputation methods. The estimated coverage and its 95% Monte Carlo confidence intervals are shown for each relative risk. They are labeled in the format "state A - state B / state C," representing the relative risk of transitioning to state B versus transitioning to state C when in state A.

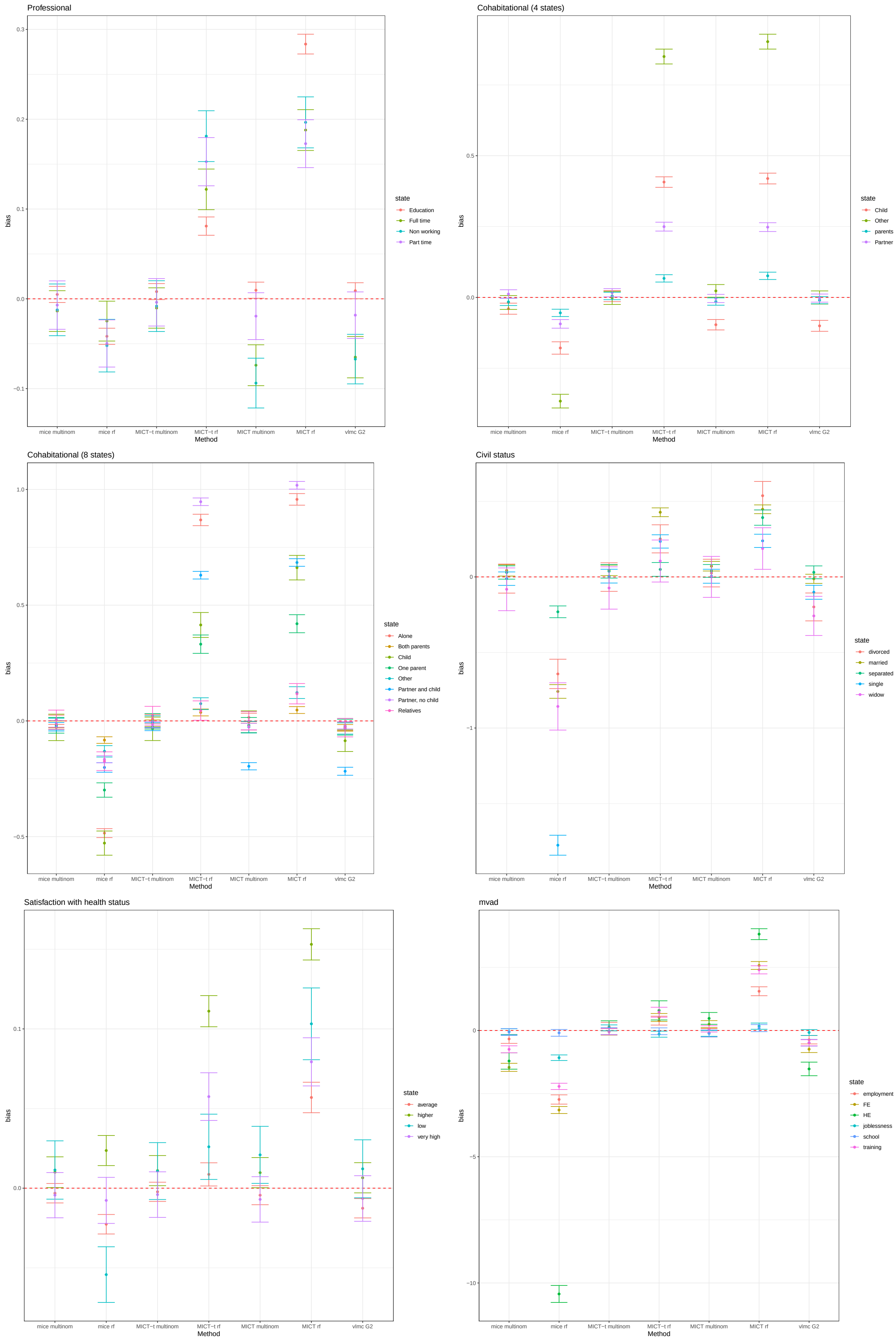

Figure A37: Results regarding the bias of the duration parameters in the case of an attrition process. Each panel displays the results of a dataset. The x-axis depicts the different imputation methods. The estimated bias and its 95% Monte Carlo confidence intervals are provided for the mean time spent in each state.

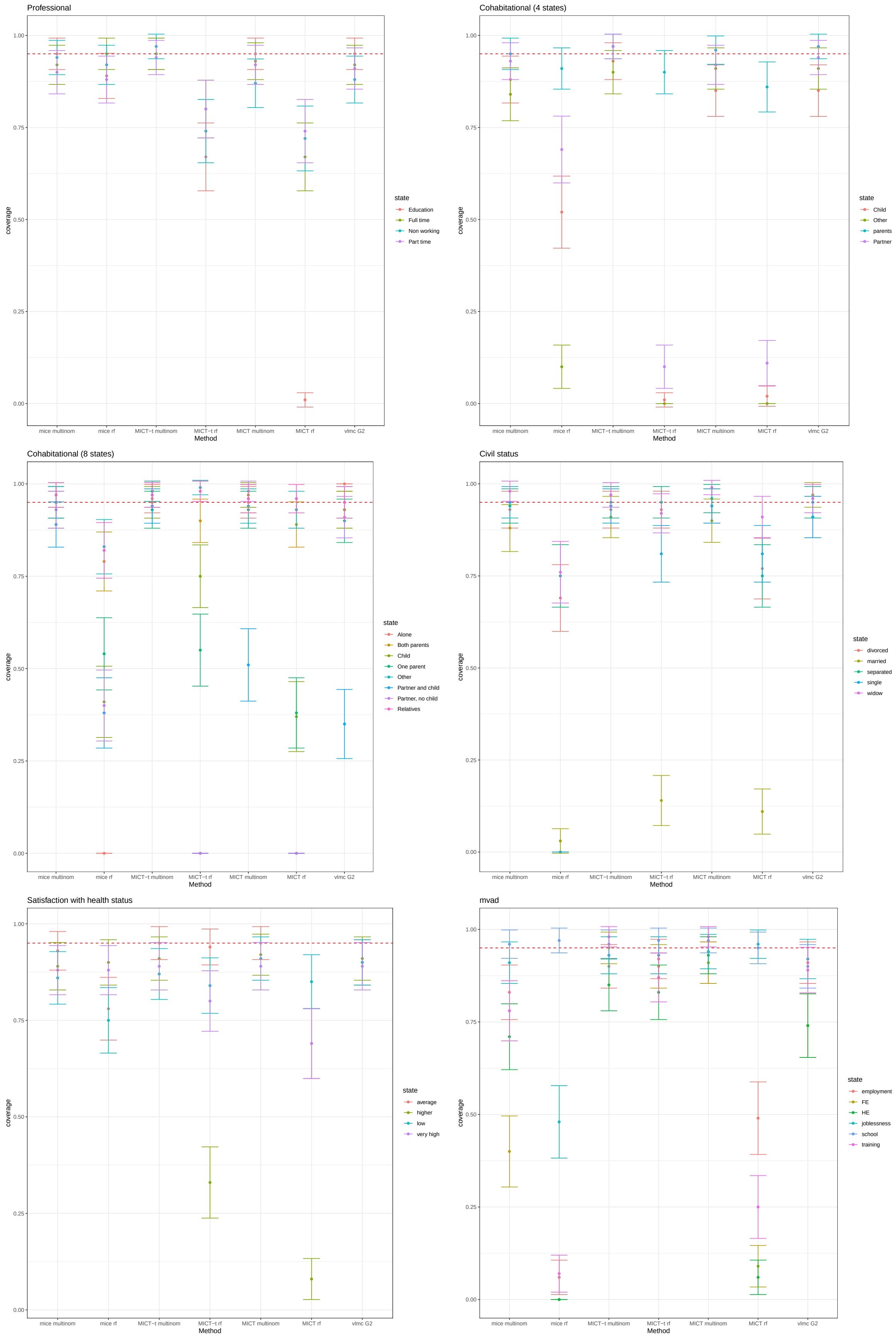

Figure A38: Results regarding the coverage of the duration parameters in the case of an attrition process. Each panel displays the results of a dataset. The x-axis depicts the different imputation methods. The estimated bias and its 95% Monte Carlo confidence intervals are provided for the mean time spent in each state.

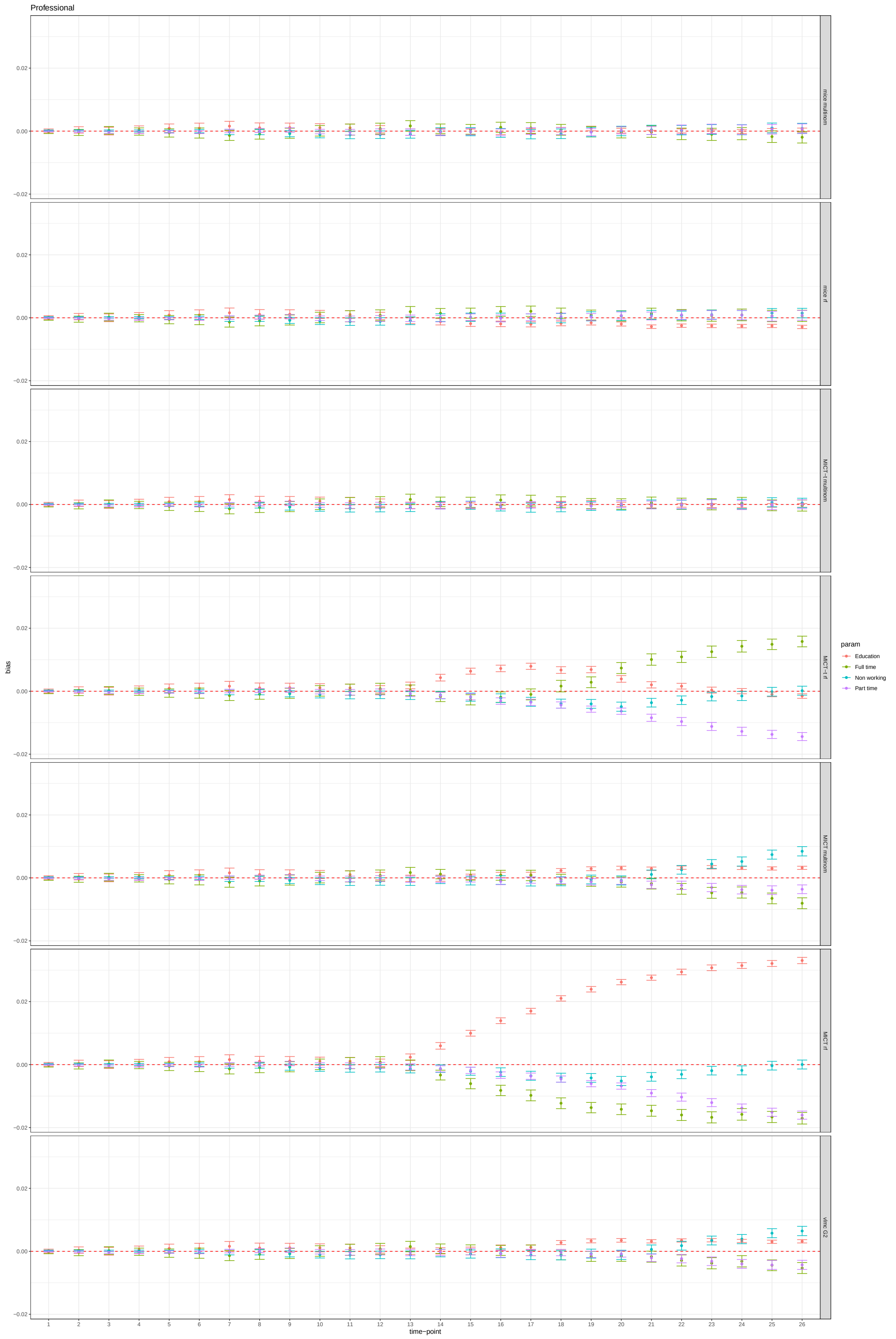

Figure A39: Results regarding the bias of the timing parameters in the case of an attrition process simulated on professional trajectories. Each panel displays the results of an imputation method. The estimated bias and its 95% Monte Carlo confidence intervals are shown for the probability of belonging to each state at each time point.

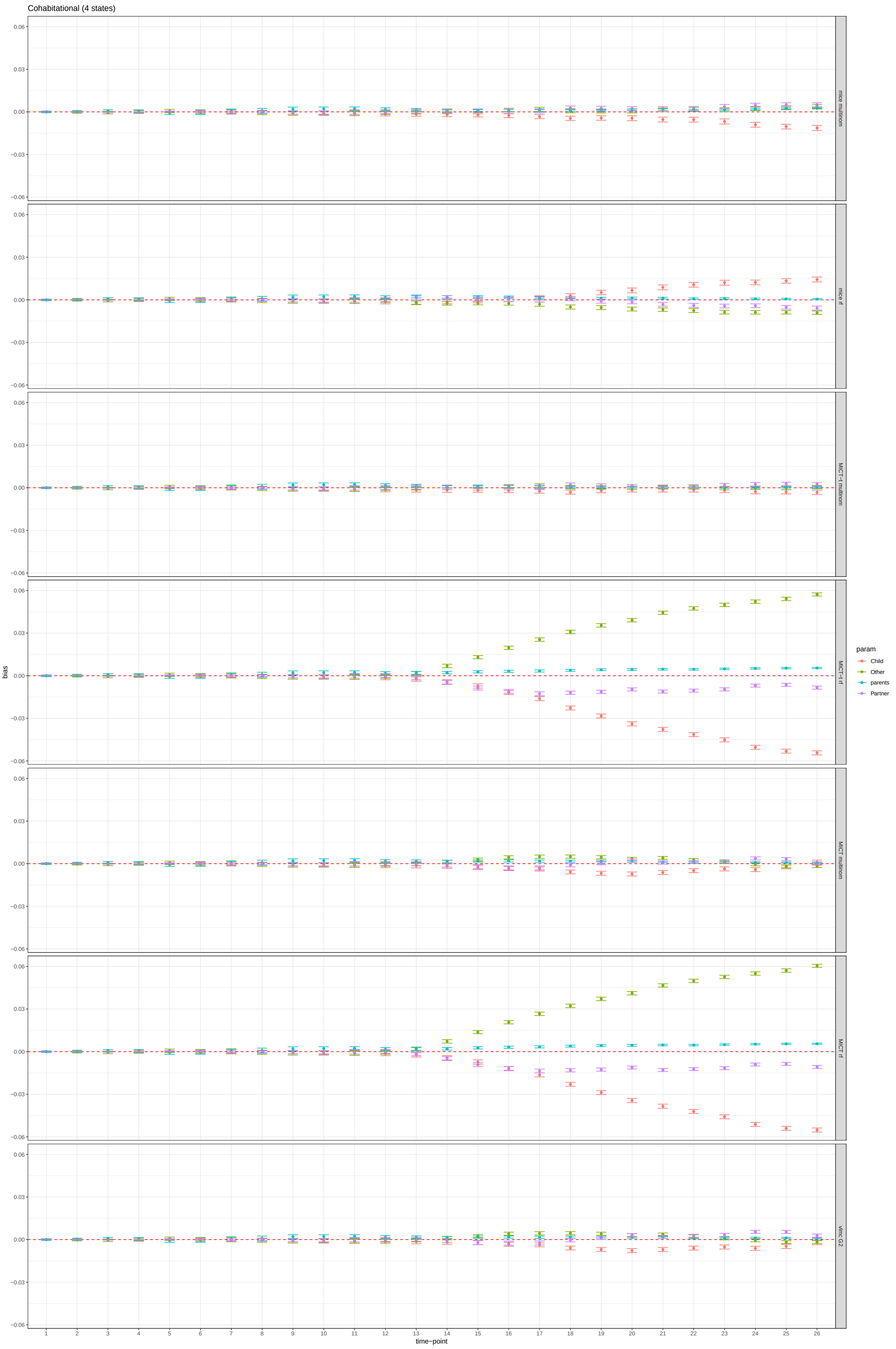

Figure A40: Results regarding the bias of the timing parameters in the case of an attrition process simulated on cohabitational status coded as four states. Each panel displays the results of an imputation method. The estimated bias and its 95% Monte Carlo confidence intervals are shown for the probability of belonging to each state at each time point.

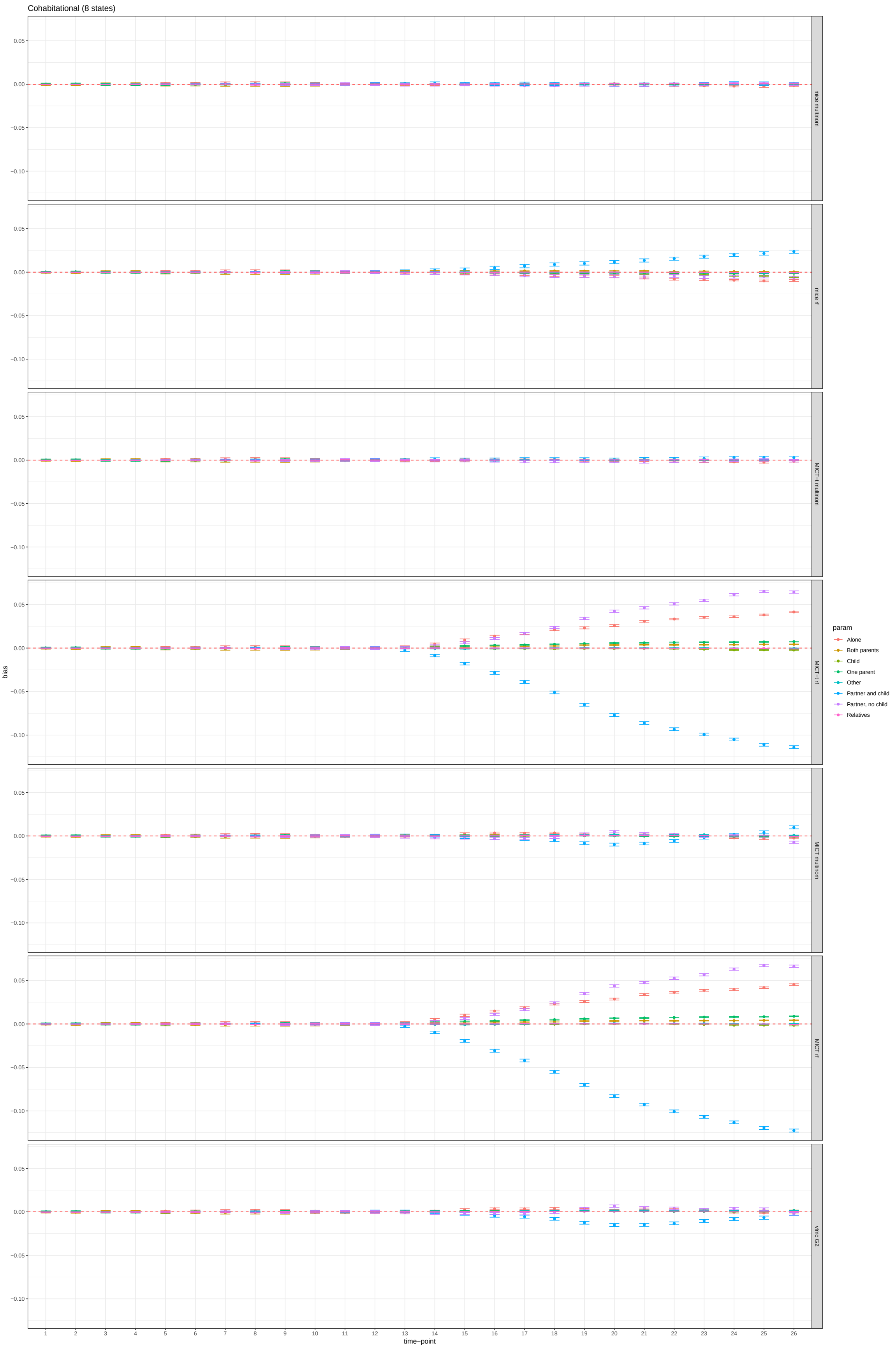

Figure A41: Results regarding the bias of the timing parameters in the case of an attrition process simulated on cohabitational status coded as eight states. Each panel displays the results of an imputation method. The estimated bias and its 95% Monte Carlo confidence intervals are shown for the probability of belonging to each state at each time point.

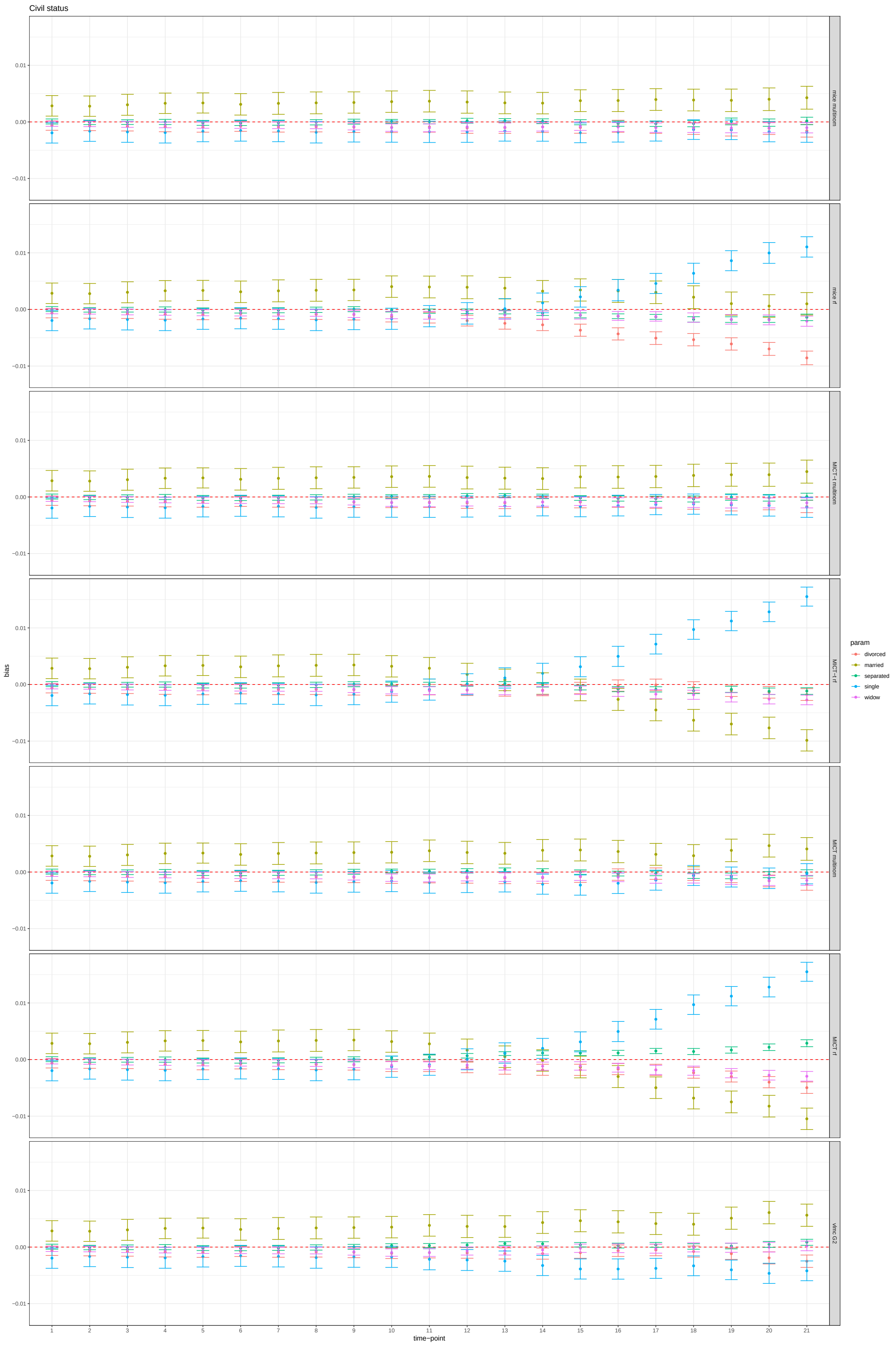

Figure A42: Results regarding the bias of the timing parameters in the case of an attrition process simulated on civil status. Each panel displays the results of an imputation method. The estimated bias and its 95% Monte Carlo confidence intervals are shown for the probability of belonging to each state at each time point.

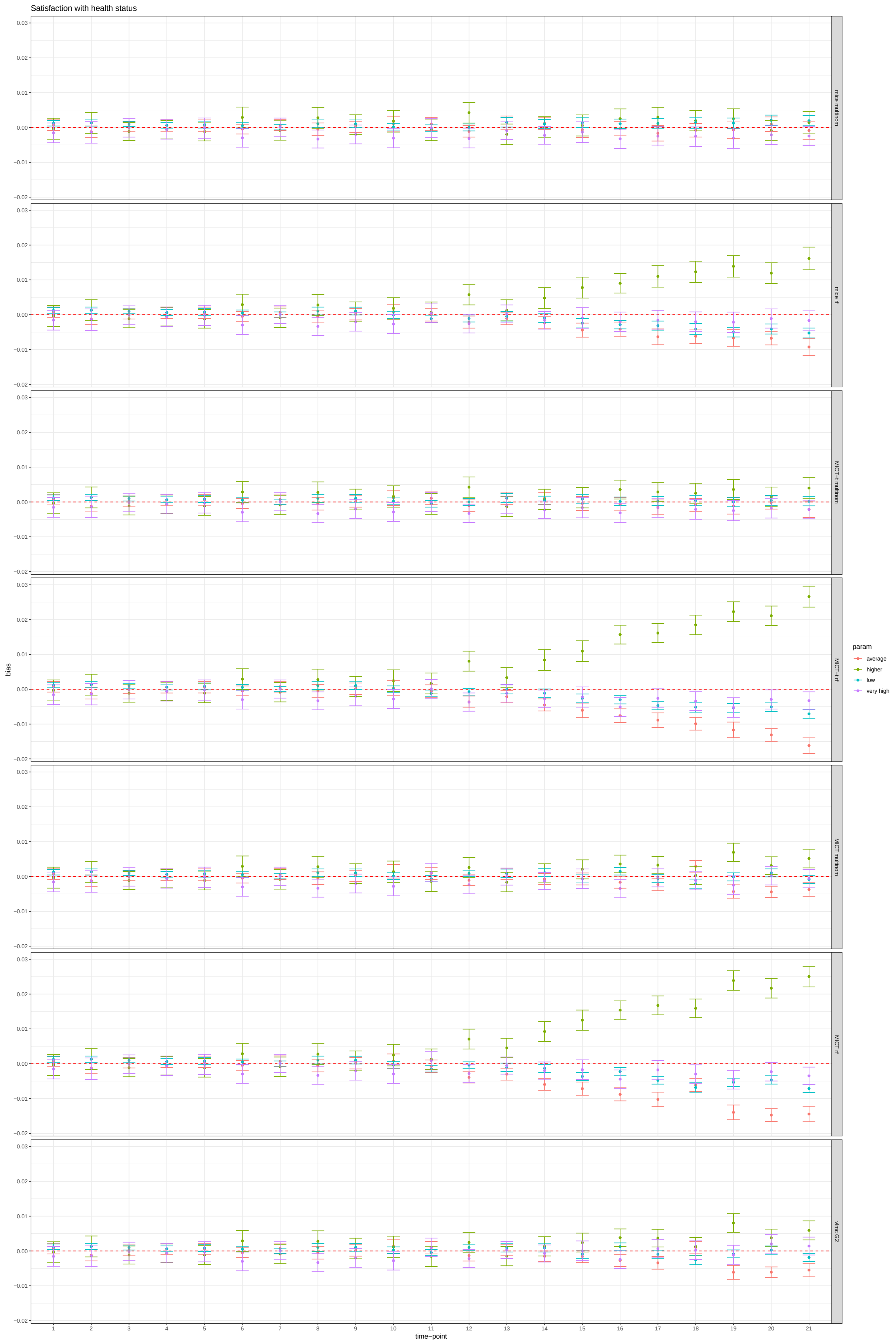

Figure A43: Results regarding the bias of the timing parameters in the case of an attrition process simulated on satisfaction with health status. Each panel displays the results of an imputation method. The estimated bias and its 95% Monte Carlo confidence intervals are shown for the probability of belonging to each state at each time point.

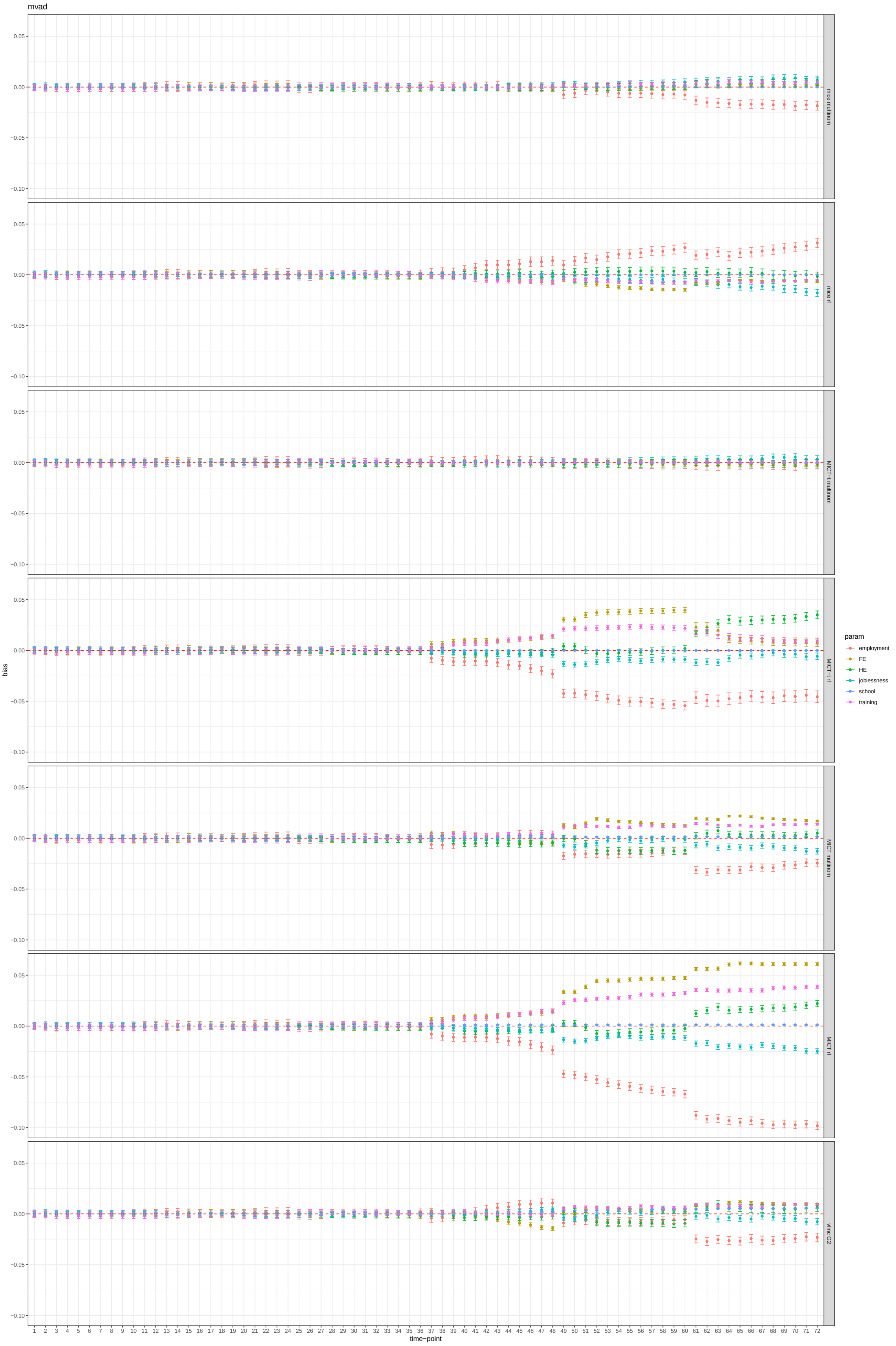

Figure A44: Results regarding the bias of the timing parameters in the case of an attrition process simulated on school-to-work transitions. Each panel displays the results of an imputation method. The estimated bias and its 95% Monte Carlo confidence intervals are shown for the probability of belonging to each state at each time point.

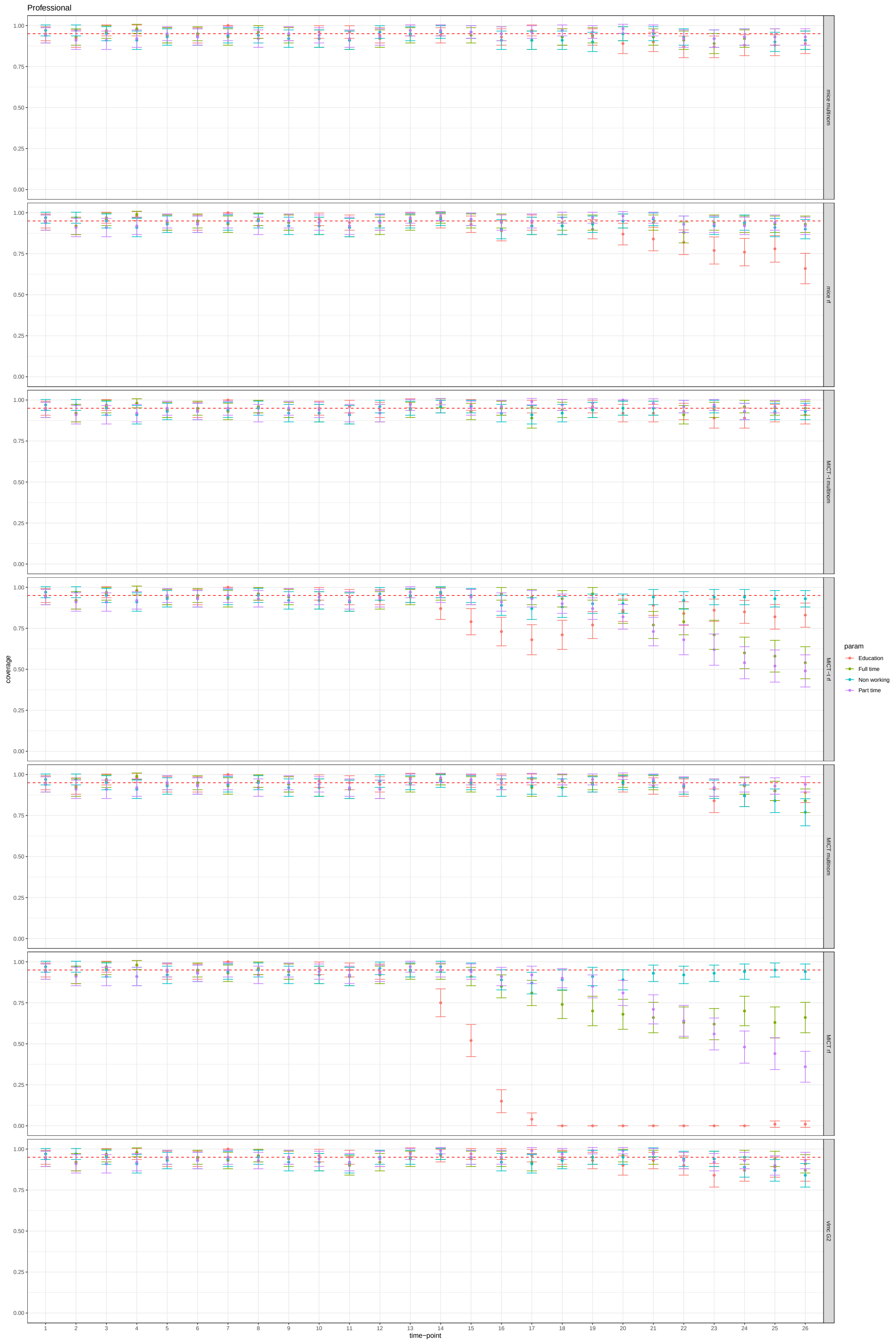

Figure A45: Results regarding the coverage of the timing parameters in the case of an attrition process simulated on professional trajectories. Each panel displays the results of an imputation method. The estimated coverage and its 95% Monte Carlo confidence intervals are shown for the probability of belonging to each state at each time point.

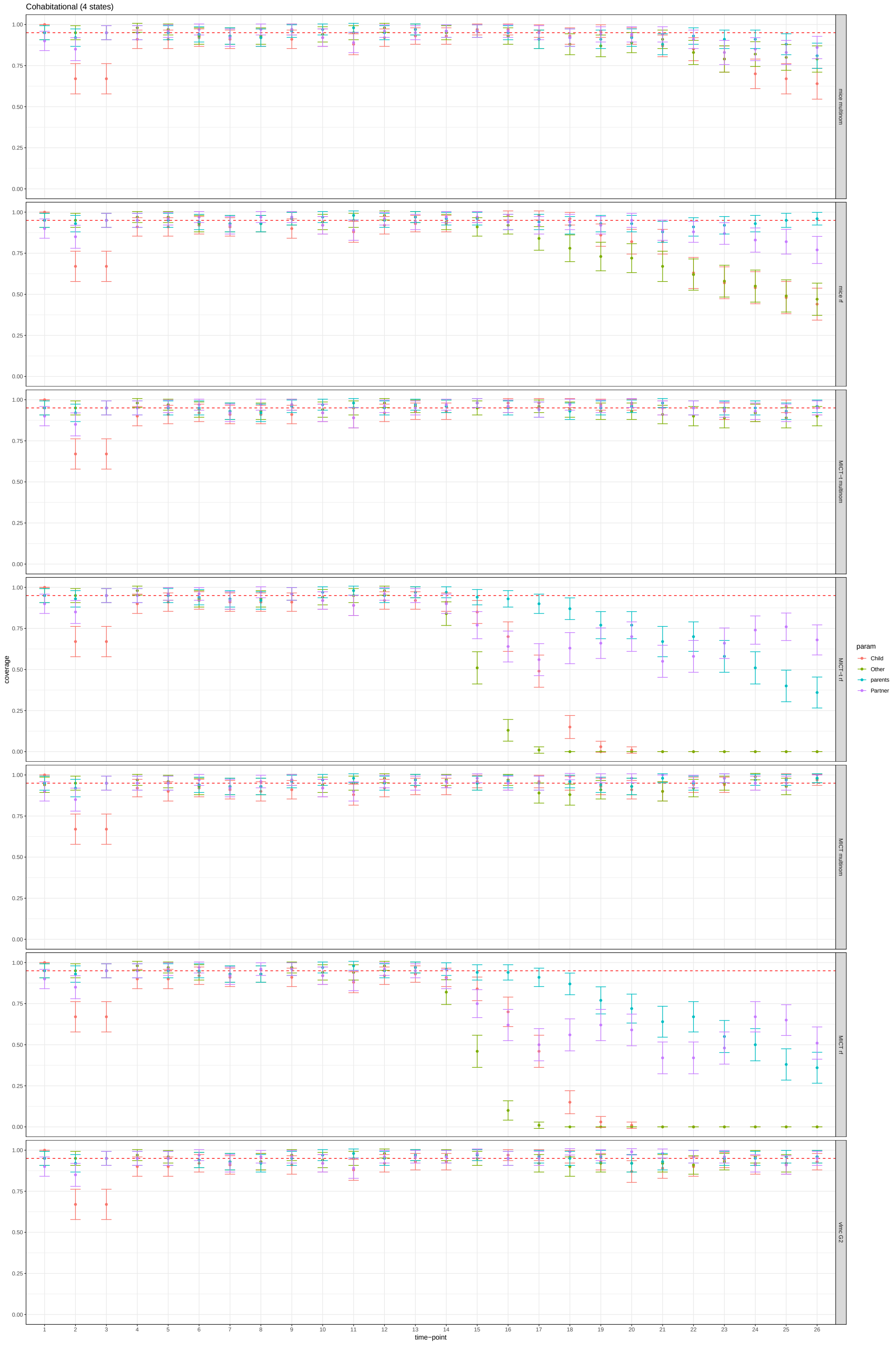

Figure A46: Results regarding the coverage of the timing parameters in the case of an attrition process simulated on cohabitational status coded as four states. Each panel displays the results of an imputation method. The estimated coverage and its 95% Monte Carlo confidence intervals are shown for the probability of belonging to each state at each time point.

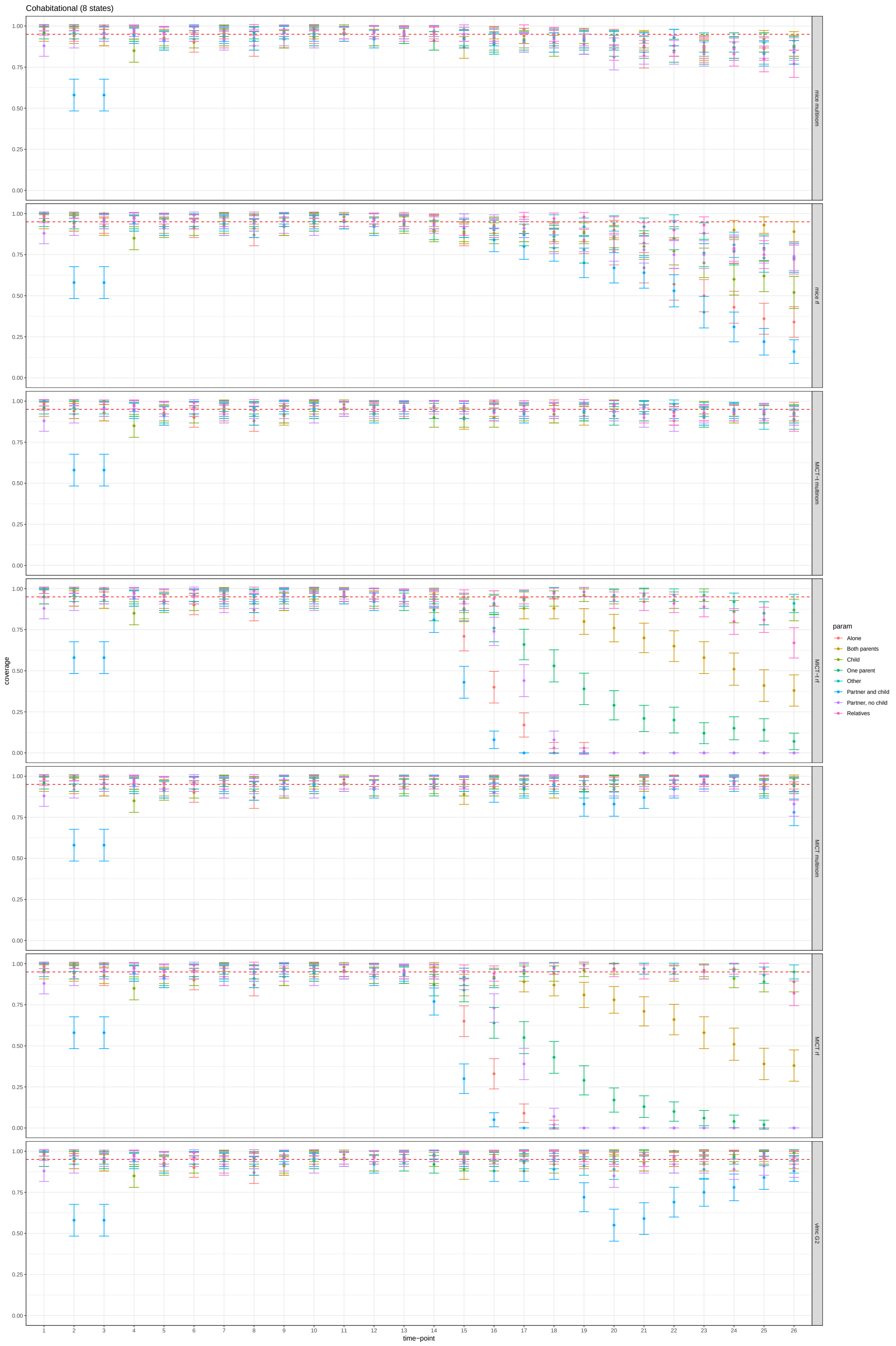

Figure A47: Results regarding the coverage of the timing parameters in the case of an attrition process simulated on cohabitational status coded as eight states. Each panel displays the results of an imputation method. The estimated coverage and its 95% Monte Carlo confidence intervals are shown for the probability of belonging to each state at each time point.

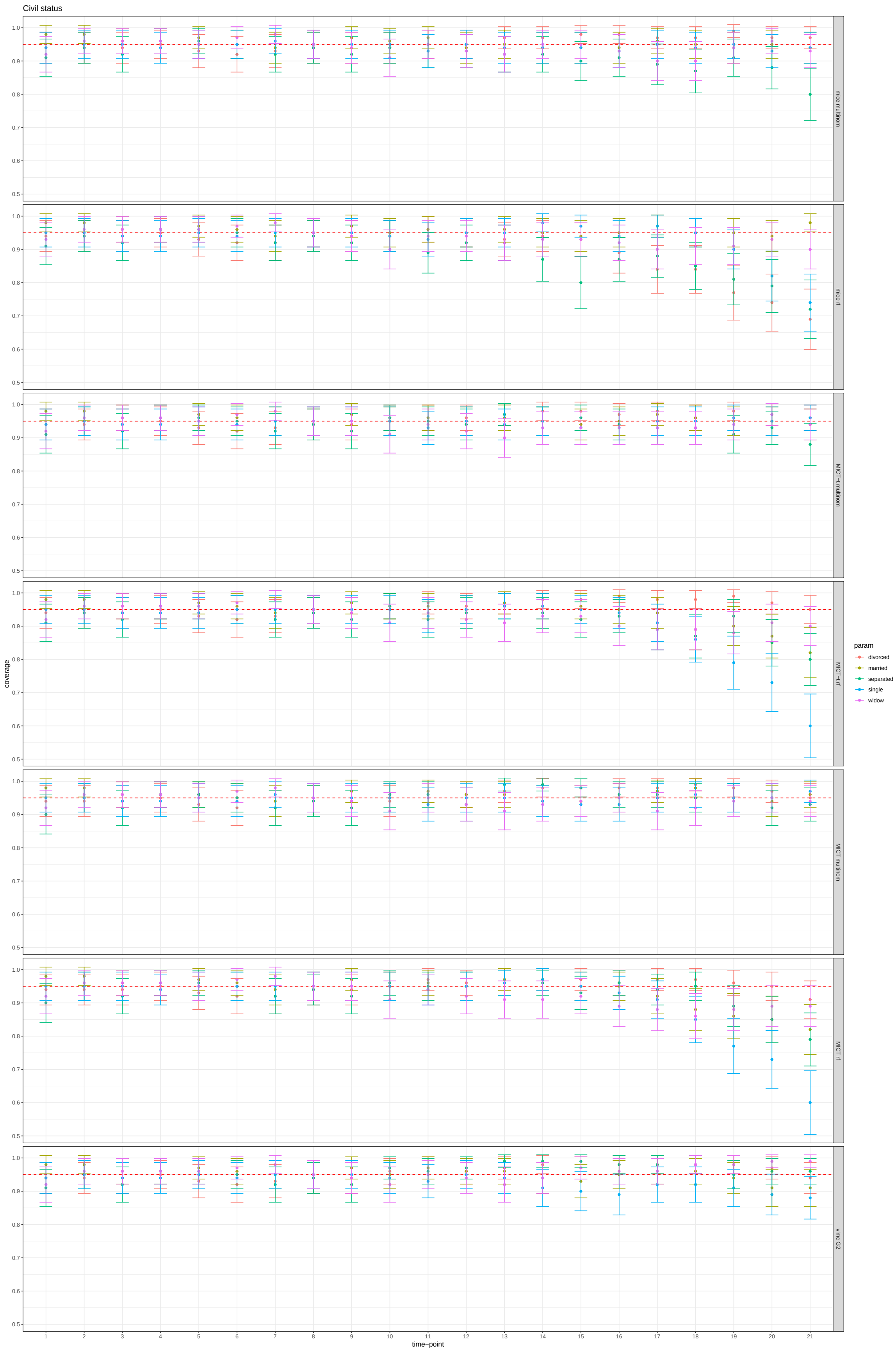

Figure A48: Results regarding the coverage of the timing parameters in the case of an attrition process simulated on civil status. Each panel displays the results of an imputation method. The estimated coverage and its 95% Monte Carlo confidence intervals are shown for the probability of belonging to each state at each time point.

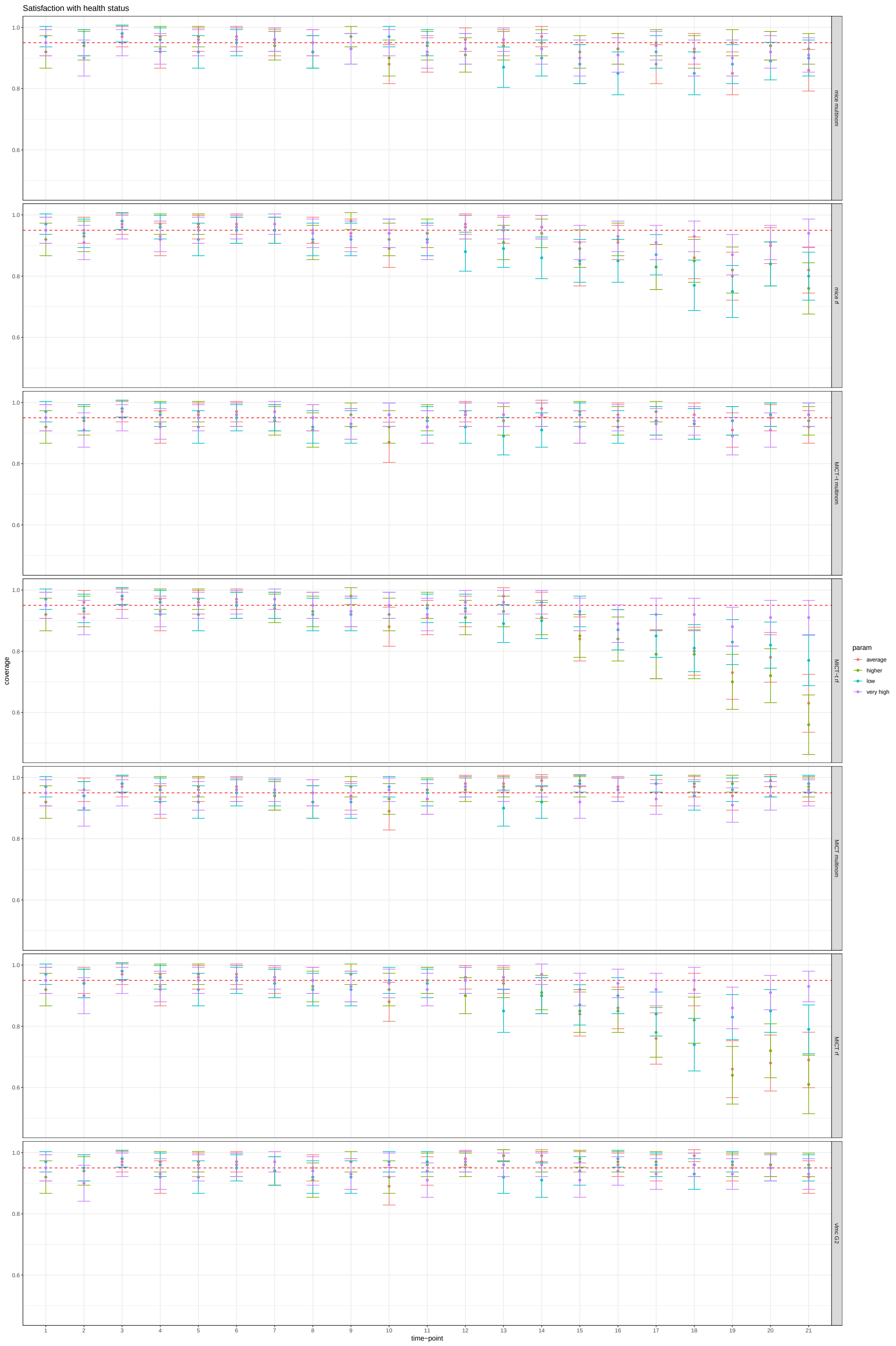

Figure A49: Results regarding the coverage of the timing parameters in the case of an attrition process simulated on satisfaction with health status. Each panel displays the results of an imputation method. The estimated coverage and its 95% Monte Carlo confidence intervals are shown for the probability of belonging to each state at each time point.

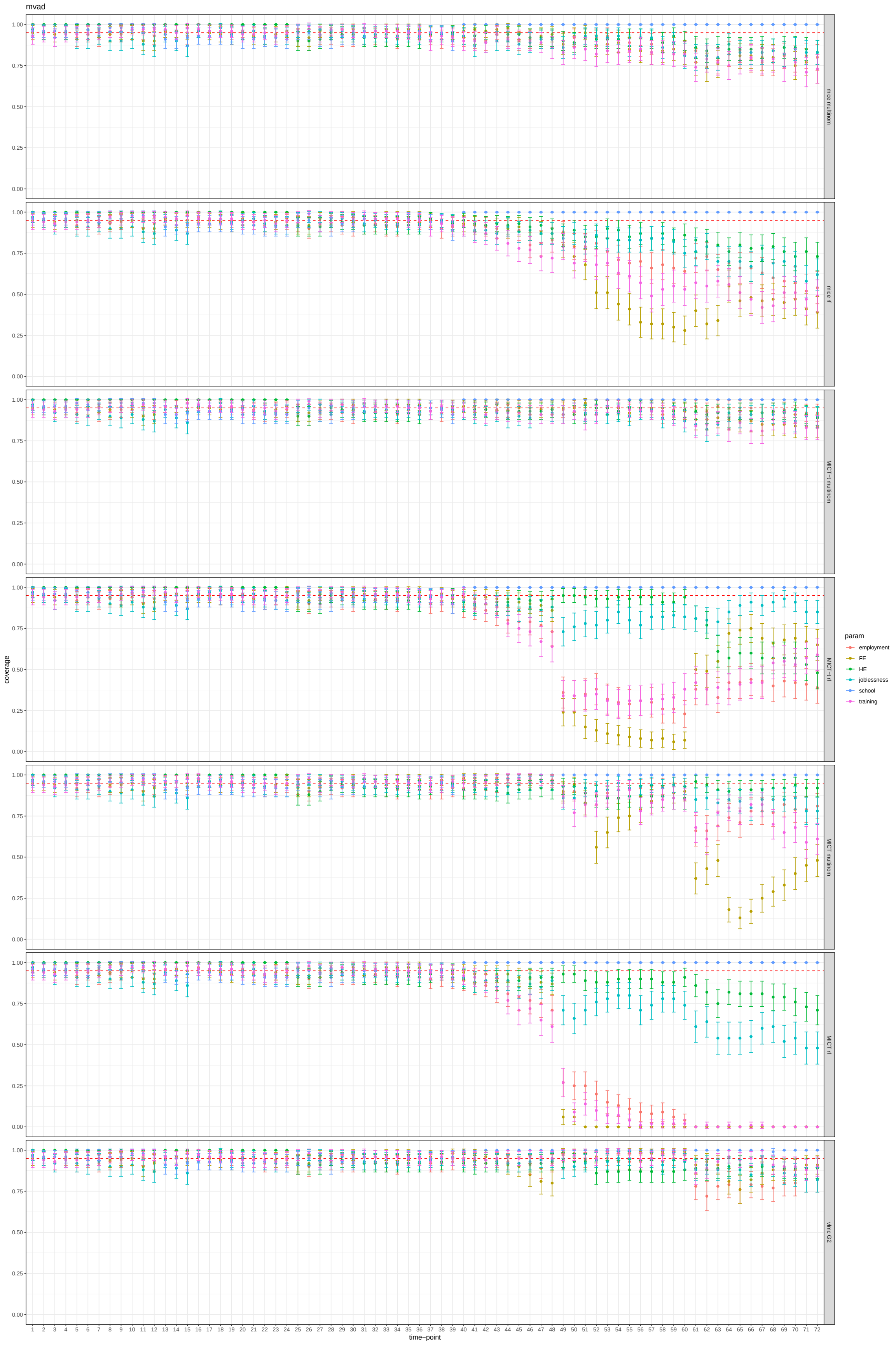

Figure A50: Results regarding the coverage of the timing parameters in the case of an attrition process simulated on school-to-work transitions. Each panel displays the results of an imputation method. The estimated coverage and its 95% Monte Carlo confidence intervals are shown for the probability of belonging to each state at each time point.

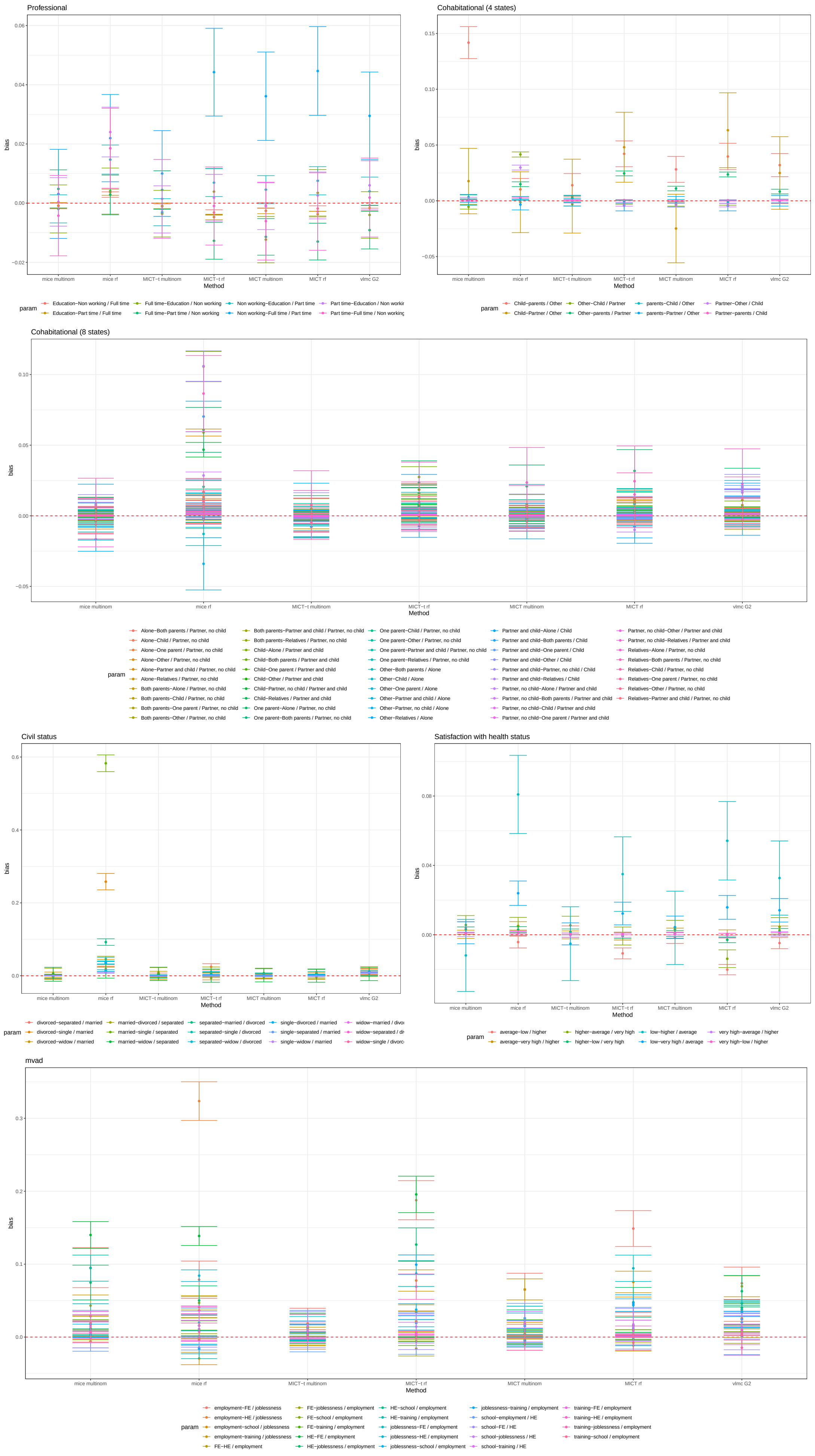

Figure A51: Results regarding the bias of the sequencing parameters in the case of an attrition process. Each panel displays the results of a dataset. The x-axis depicts the different imputation methods. The x-axis depicts the different imputation methods. The estimated bias and its 95% Monte Carlo confidence intervals are shown for each relative risk. They are labeled in the format "state A - state B / state C," representing the relative risk of transitioning to state B versus transitioning to state C when in state A.

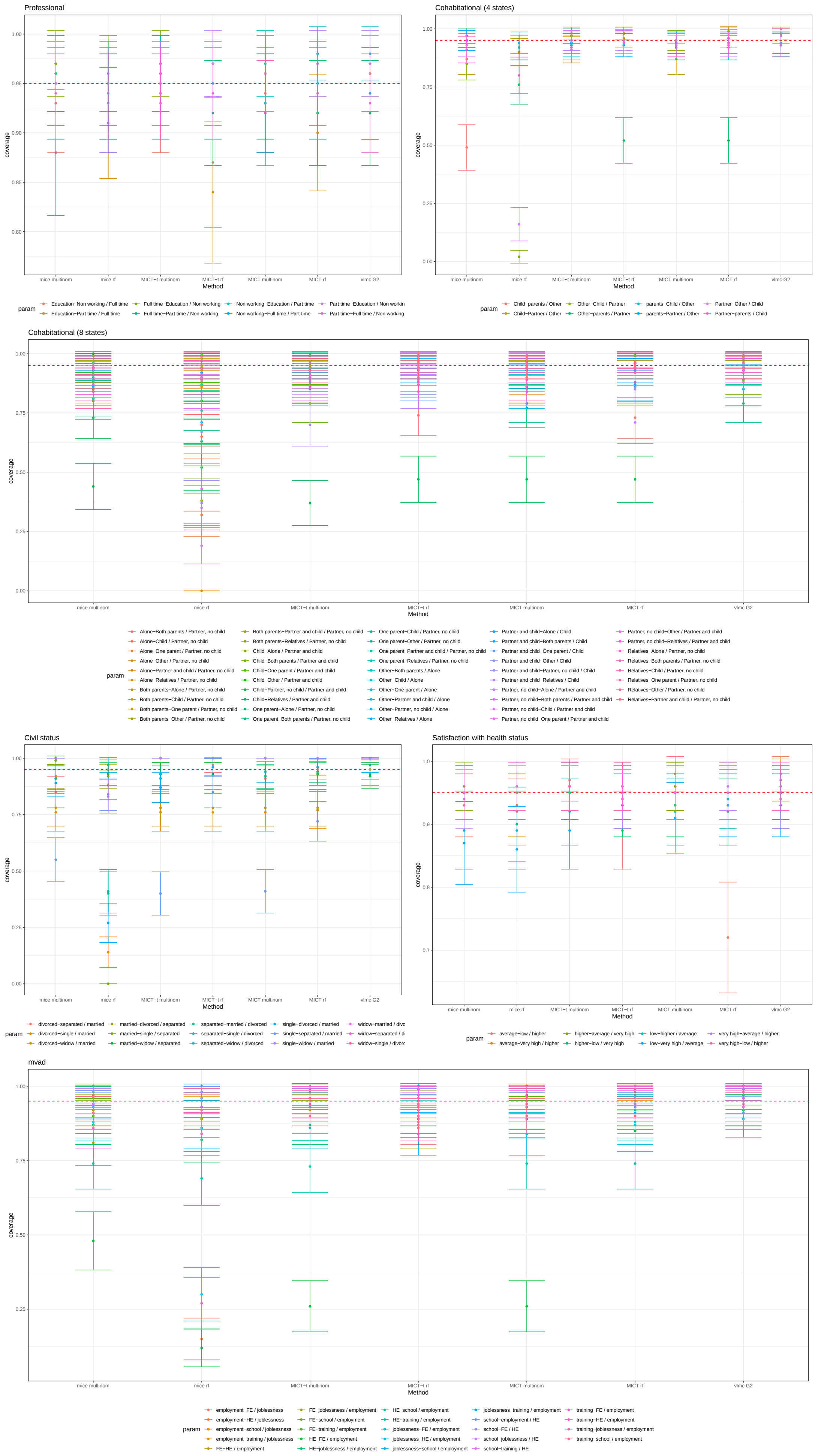

Figure A52: Results regarding the coverage of the sequencing parameters in the case of an attrition process. Each panel displays the results of a dataset. The x-axis depicts the different imputation methods. The x-axis depicts the different imputation methods. The estimated coverage and its 95% Monte Carlo confidence intervals are shown for each relative risk. They are labeled in the format "state A - state B / state C," representing the relative risk of transitioning to state B versus transitioning to state C when in state A.

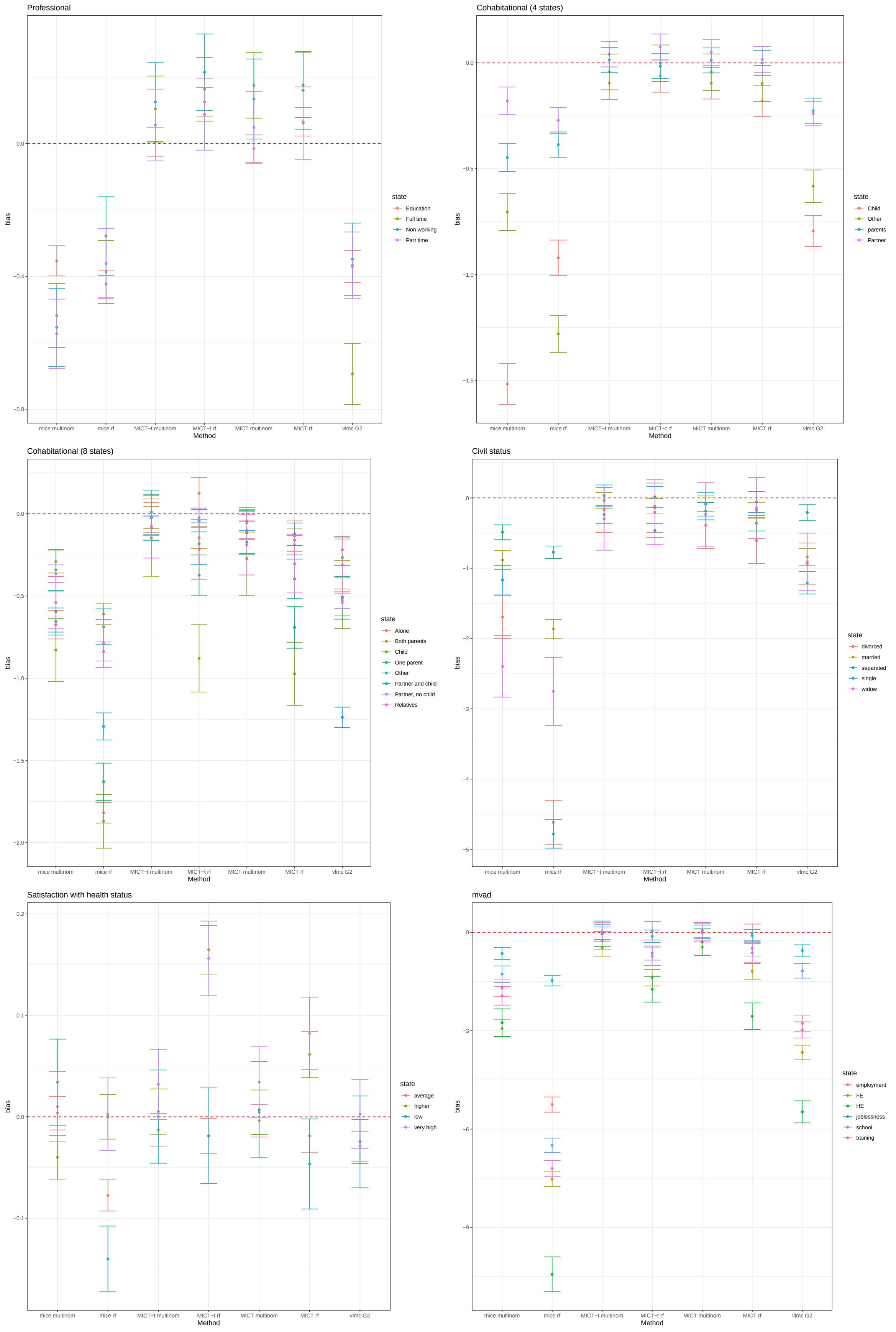

Figure A53: Results regarding the bias of the duration parameters in the case of an small missing data. Each panel displays the results of a dataset. The x-axis depicts the different imputation methods. The estimated bias and its 95% Monte Carlo confidence intervals are provided for the mean time spent in each state.

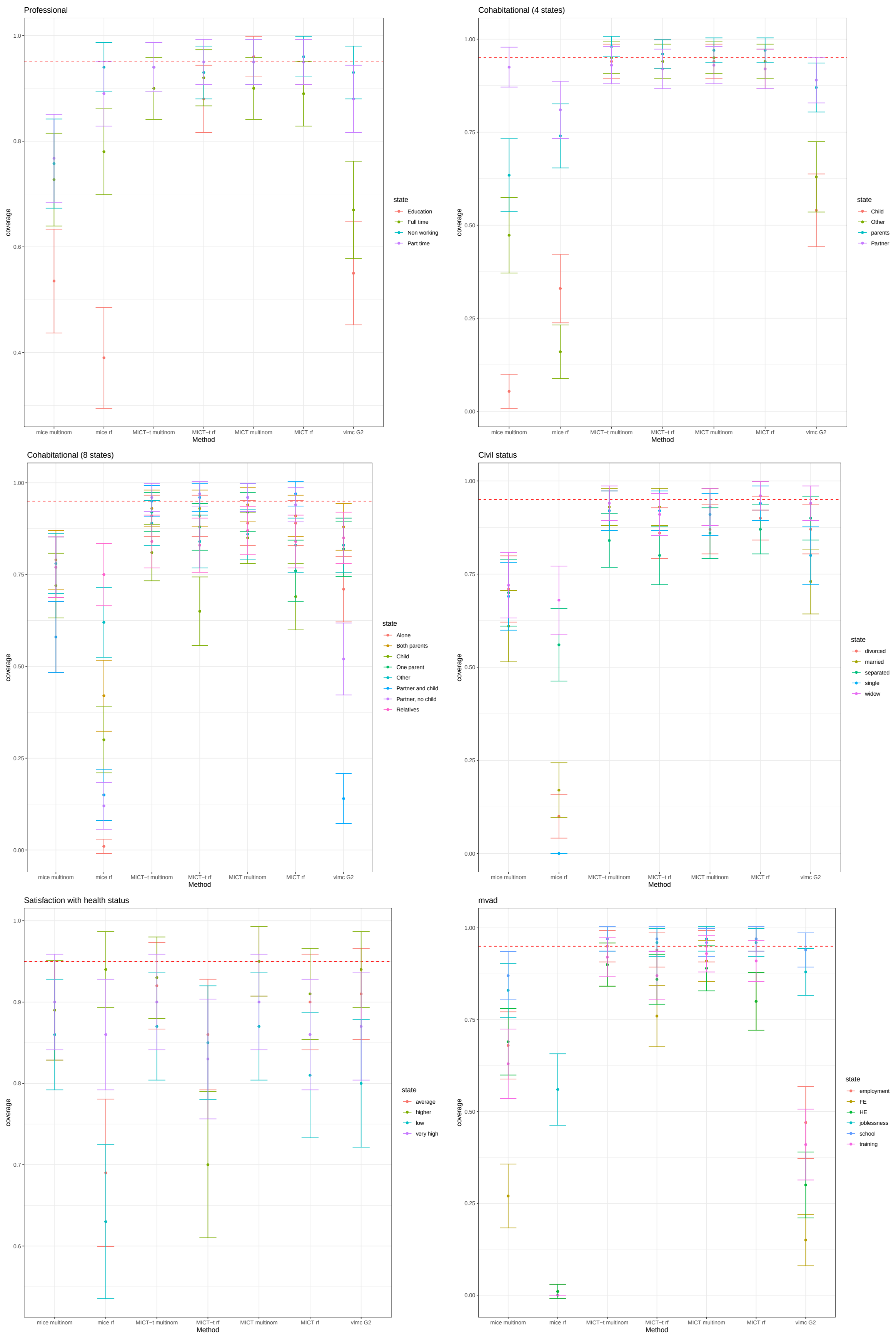

Figure A54: Results regarding the coverage of the duration parameters in the case of an small missing data. Each panel displays the results of a dataset. The x-axis depicts the different imputation methods. The estimated bias and its 95% Monte Carlo confidence intervals are provided for the mean time spent in each state.

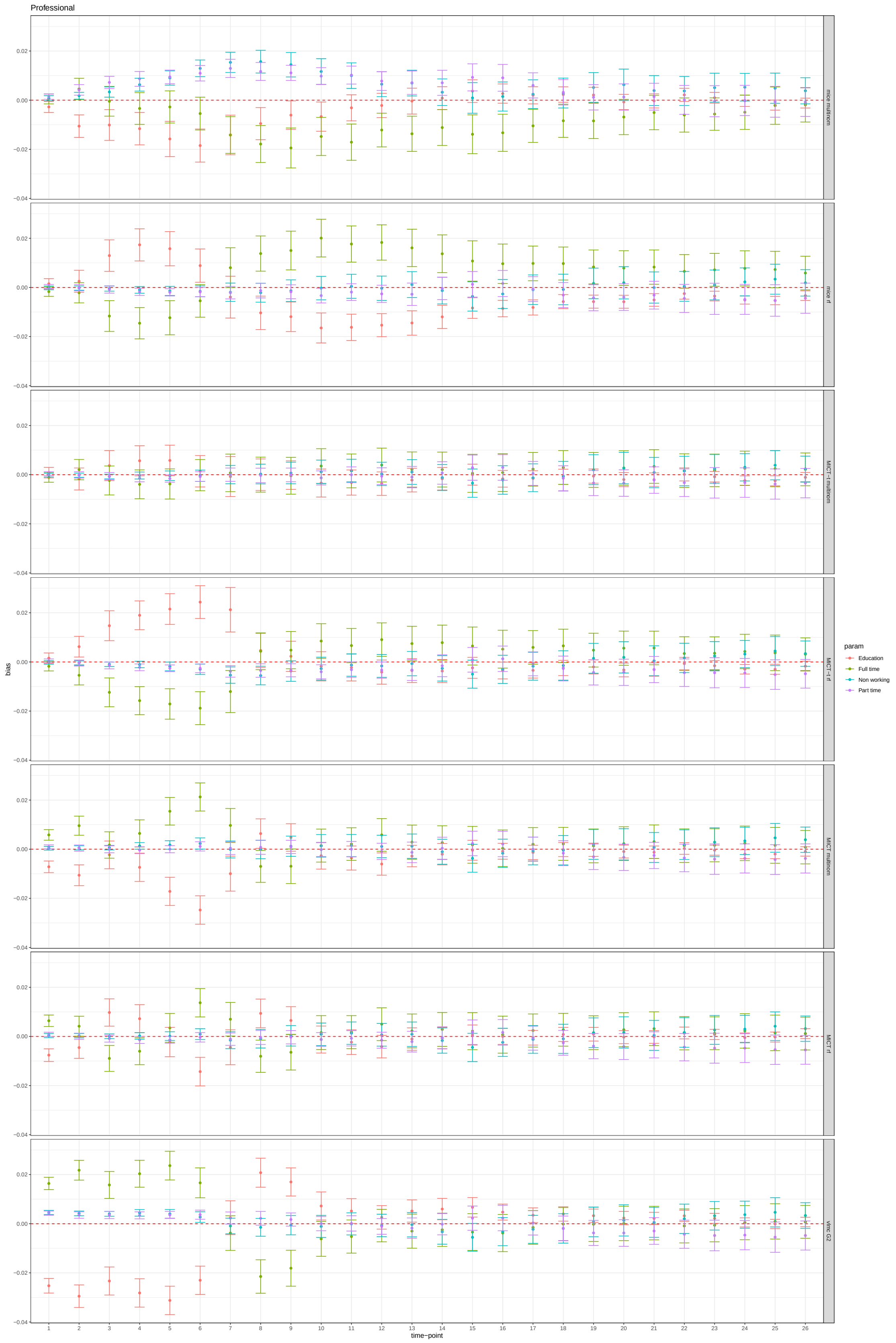

Figure A55: Results regarding the bias of the timing parameters in the case of an small sample missing data process simulated on professional trajectories. Each panel displays the results of an imputation method. The estimated bias and its 95% Monte Carlo confidence intervals are shown for the probability of belonging to each state at each time point.

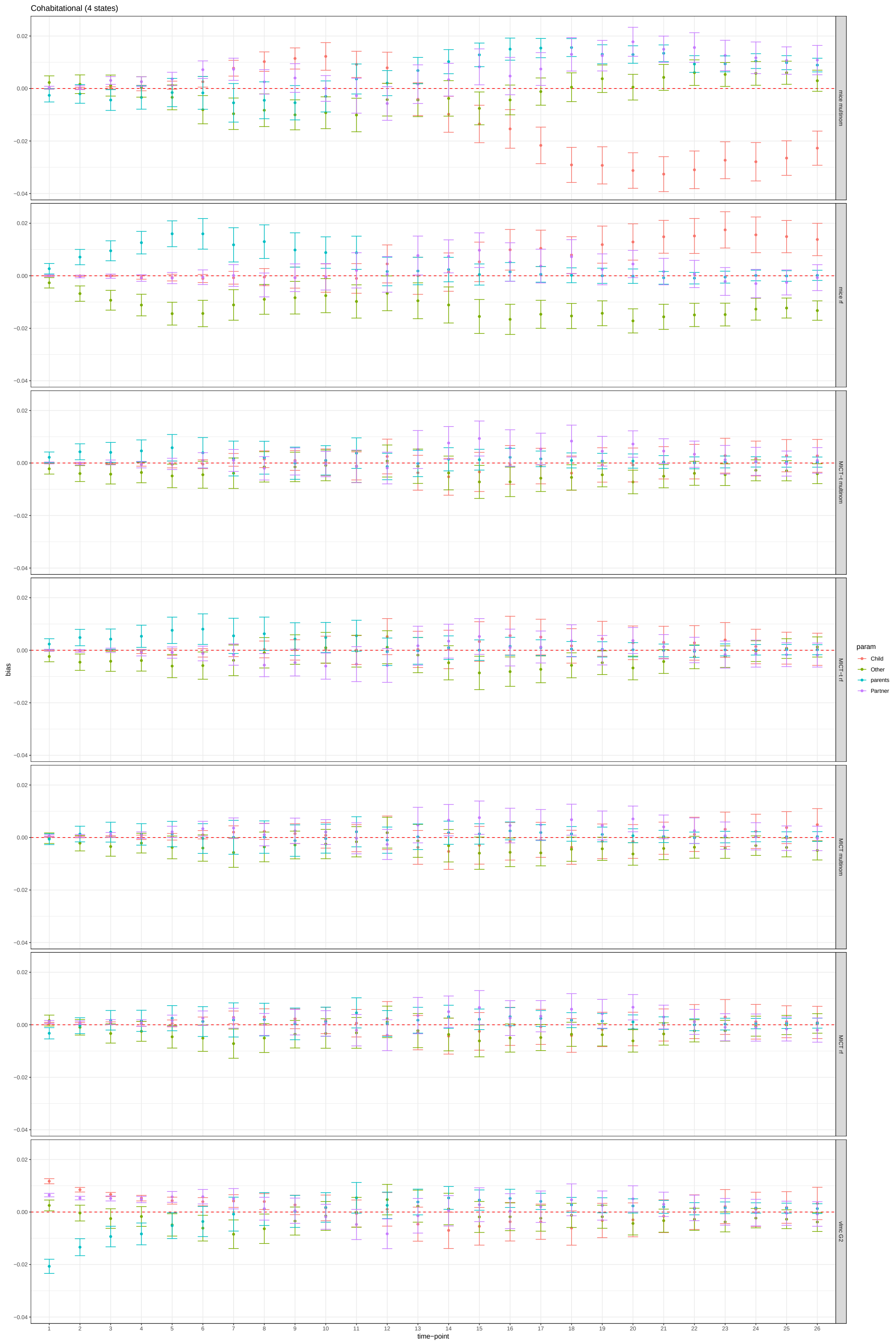

Figure A56: Results regarding the bias of the timing parameters in the case of an small sample missing data process simulated on cohabitational status coded as four states. Each panel displays the results of an imputation method. The estimated bias and its 95% Monte Carlo confidence intervals are shown for the probability of belonging to each state at each time point.

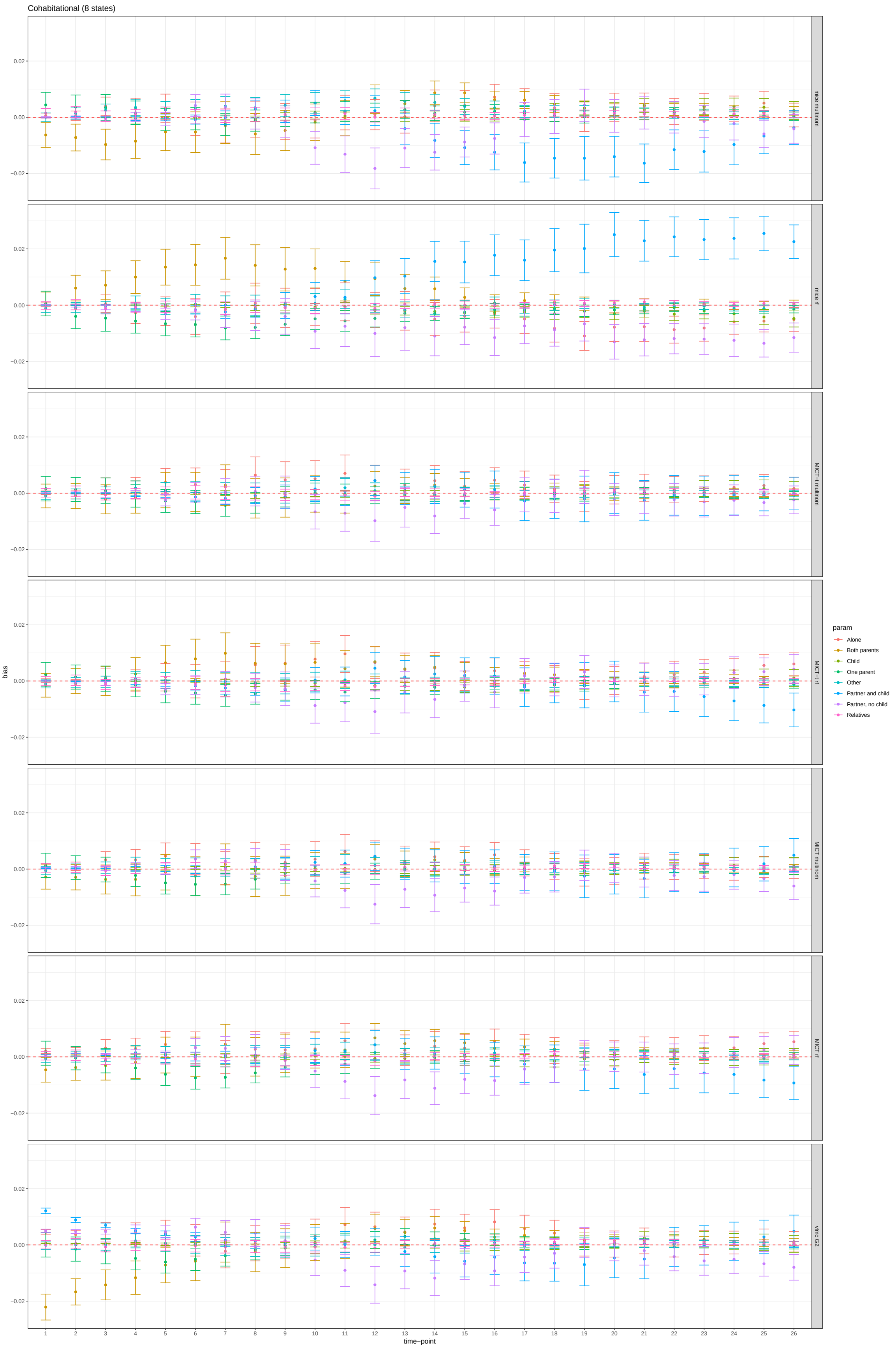

Figure A57: Results regarding the bias of the timing parameters in the case of an small sample missing data process simulated on cohabitational status coded as eight states. Each panel displays the results of an imputation method. The estimated bias and its 95% Monte Carlo confidence intervals are shown for the probability of belonging to each state at each time point.

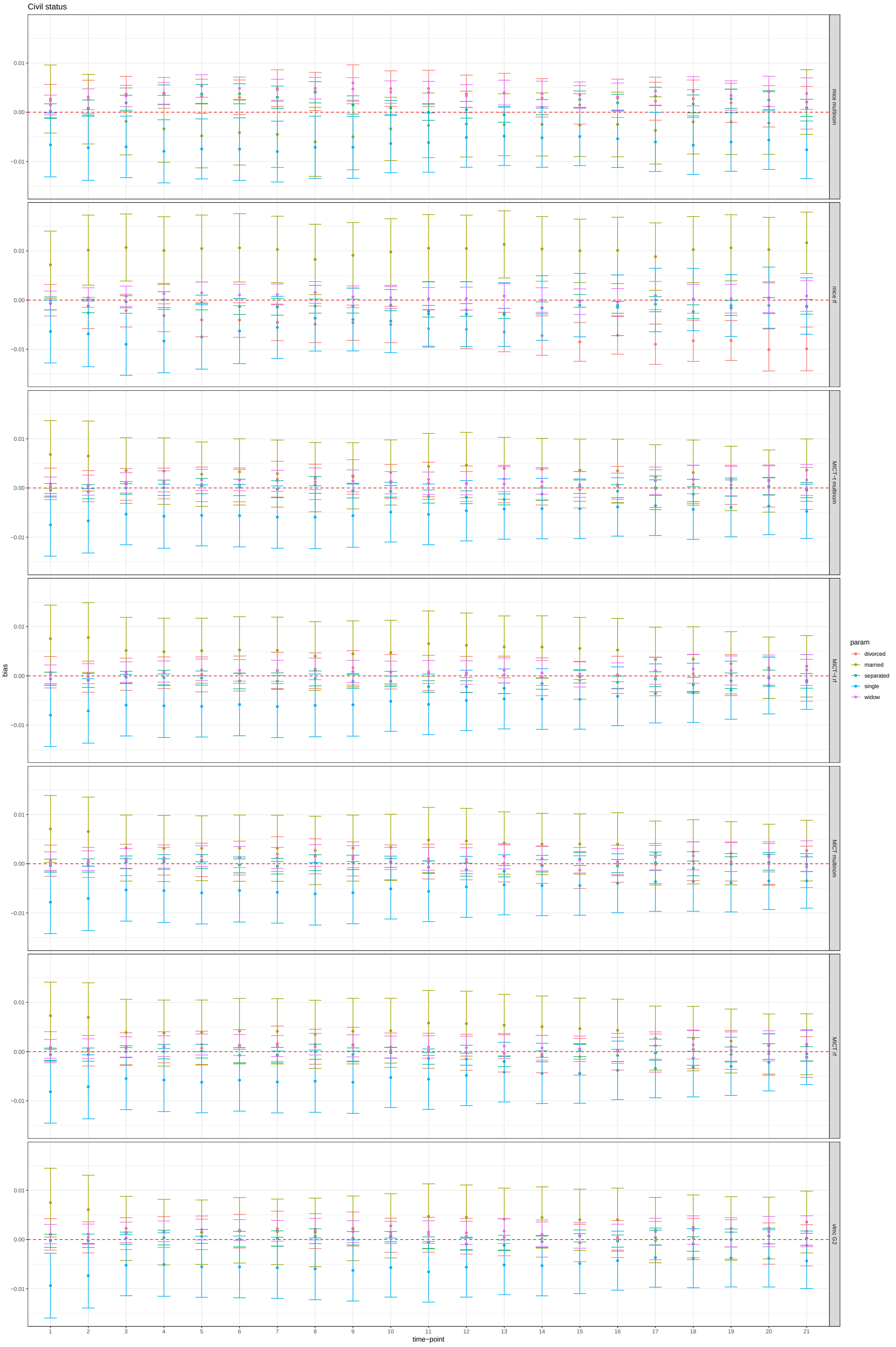

Figure A58: Results regarding the bias of the timing parameters in the case of an small sample missing data process simulated on civil status. Each panel displays the results of an imputation method. The estimated bias and its 95% Monte Carlo confidence intervals are shown for the probability of belonging to each state at each time point.

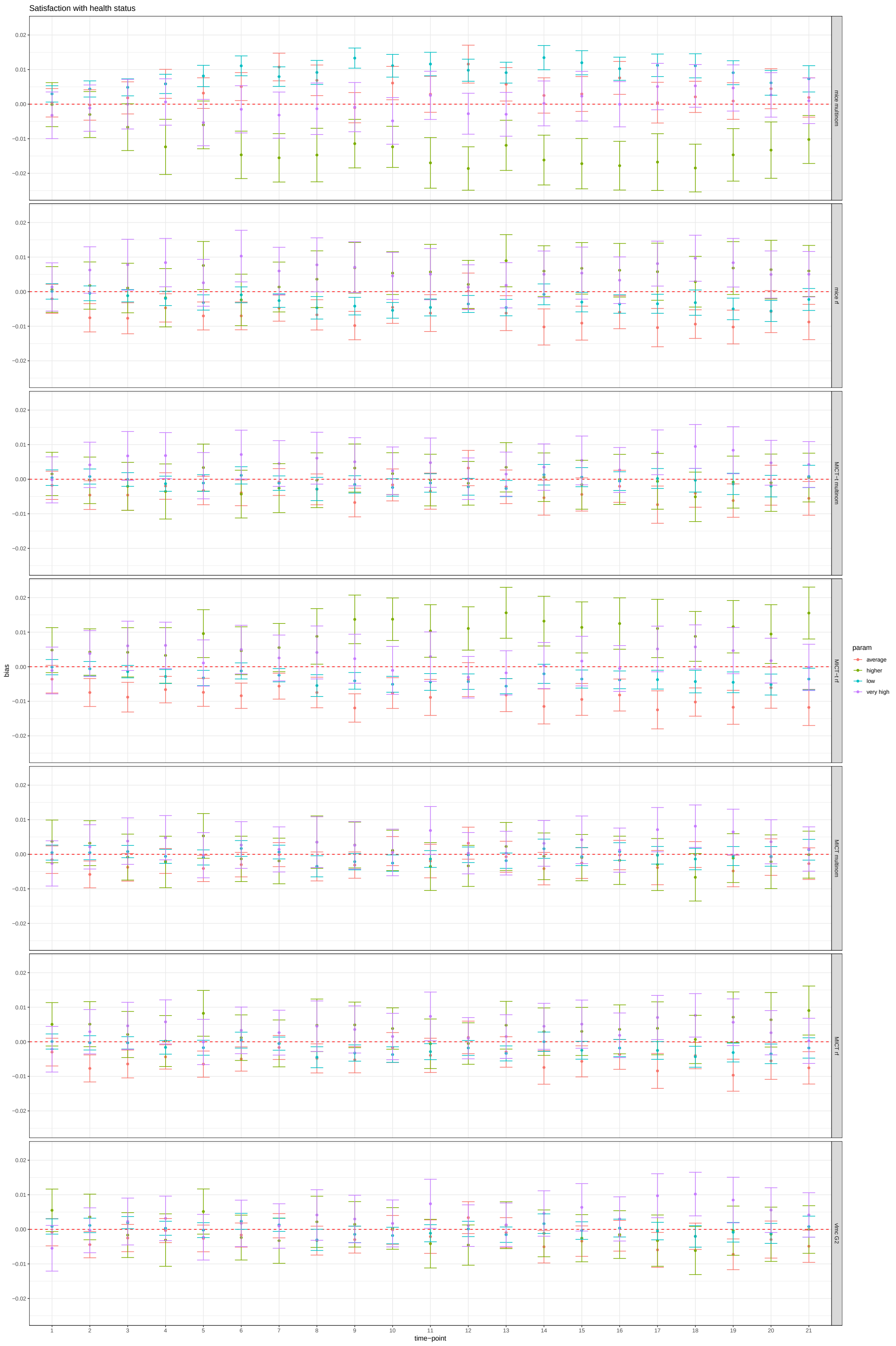

Figure A59: Results regarding the bias of the timing parameters in the case of an small sample missing data process simulated on satisfaction with health status. Each panel displays the results of an imputation method. The estimated bias and its 95% Monte Carlo confidence intervals are shown for the probability of belonging to each state at each time point.

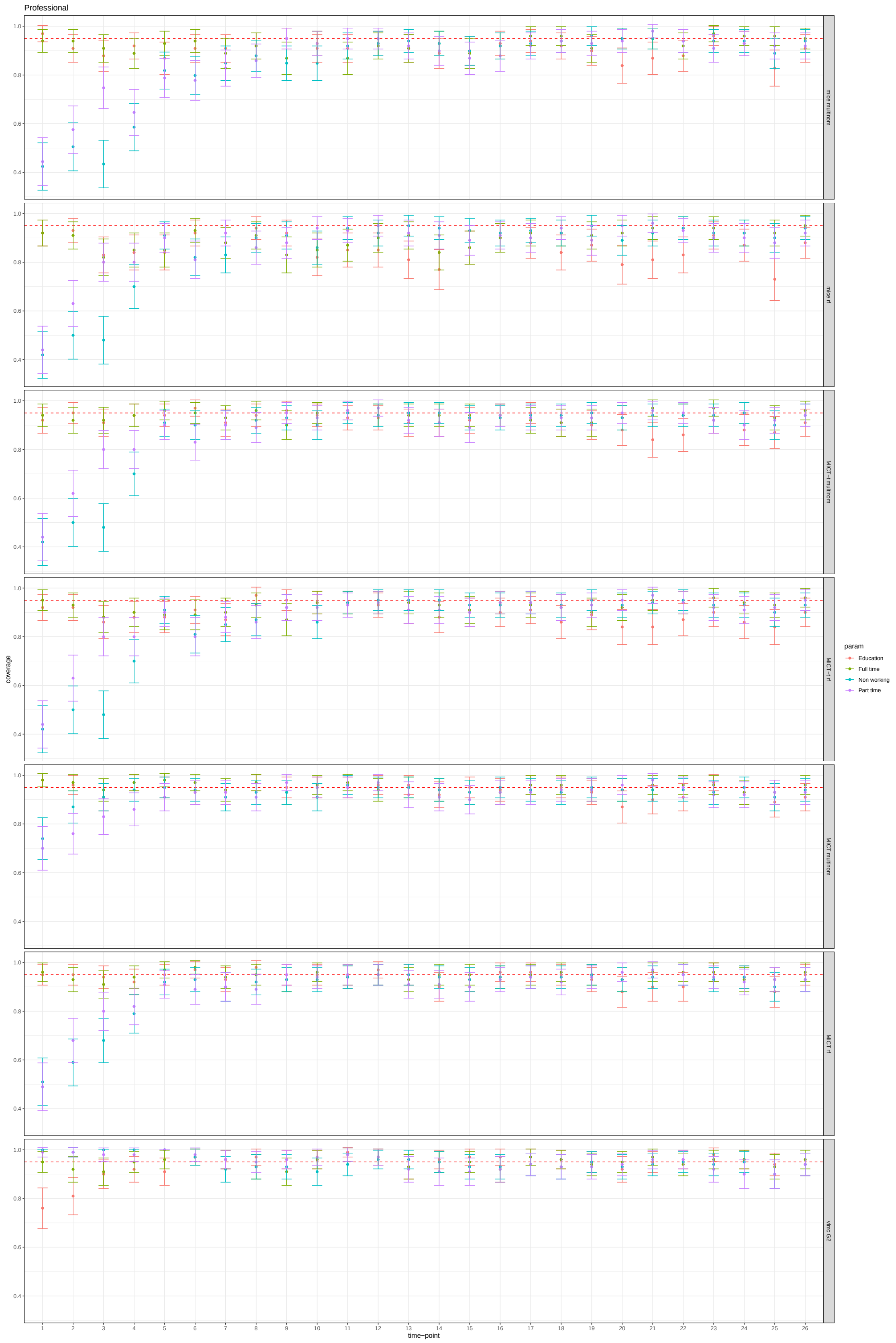

Figure A60: Results regarding the coverage of the timing parameters in the case of an small sample missing data process simulated on professional trajectories. Each panel displays the results of an imputation method. The estimated coverage and its 95% Monte Carlo confidence intervals are shown for the probability of belonging to each state at each time point.

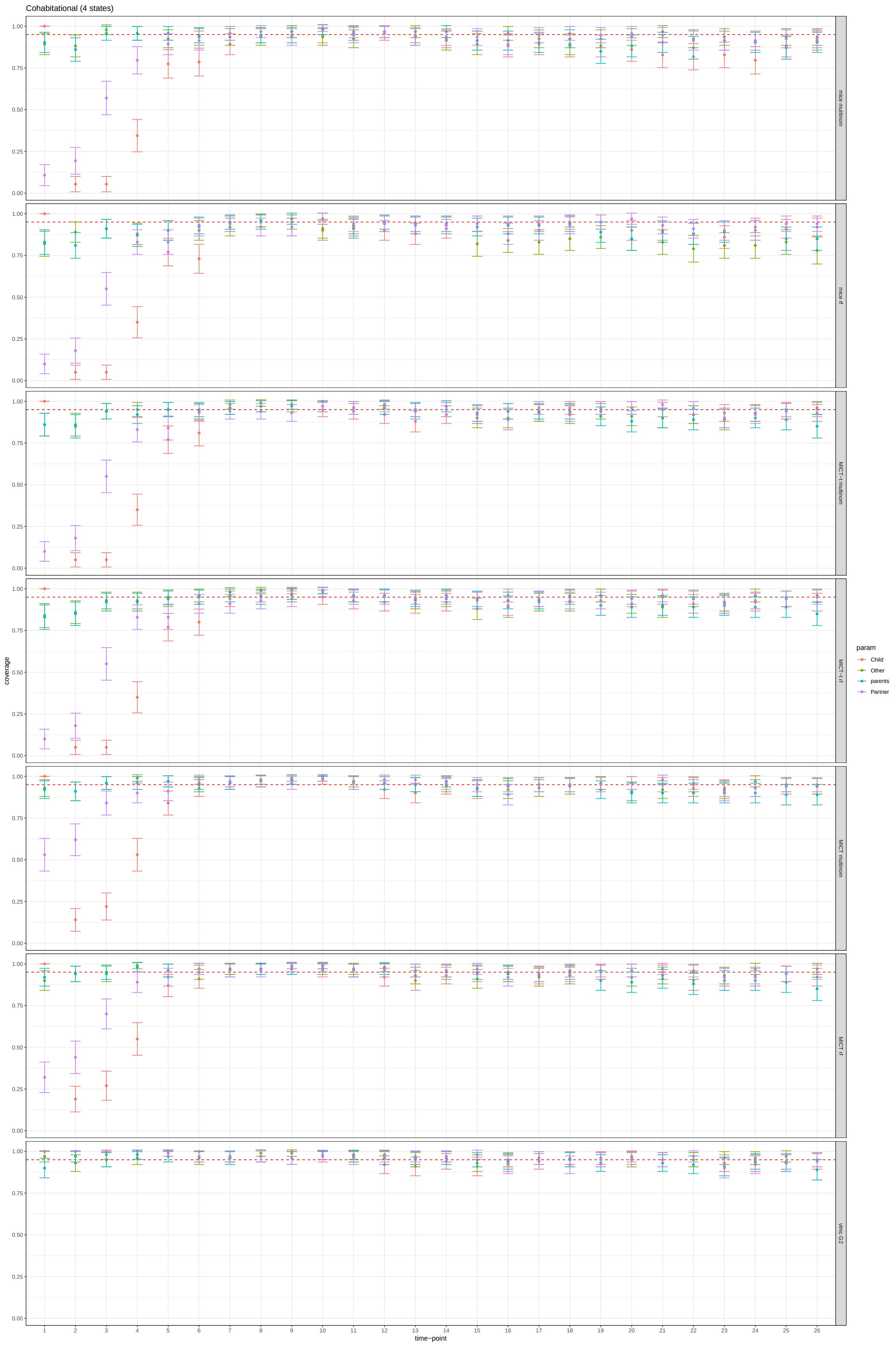

Figure A61: Results regarding the coverage of the timing parameters in the case of an small sample missing data process simulated on cohabitational status coded as four states. Each panel displays the results of an imputation method. The estimated coverage and its 95% Monte Carlo confidence intervals are shown for the probability of belonging to each state at each time point.

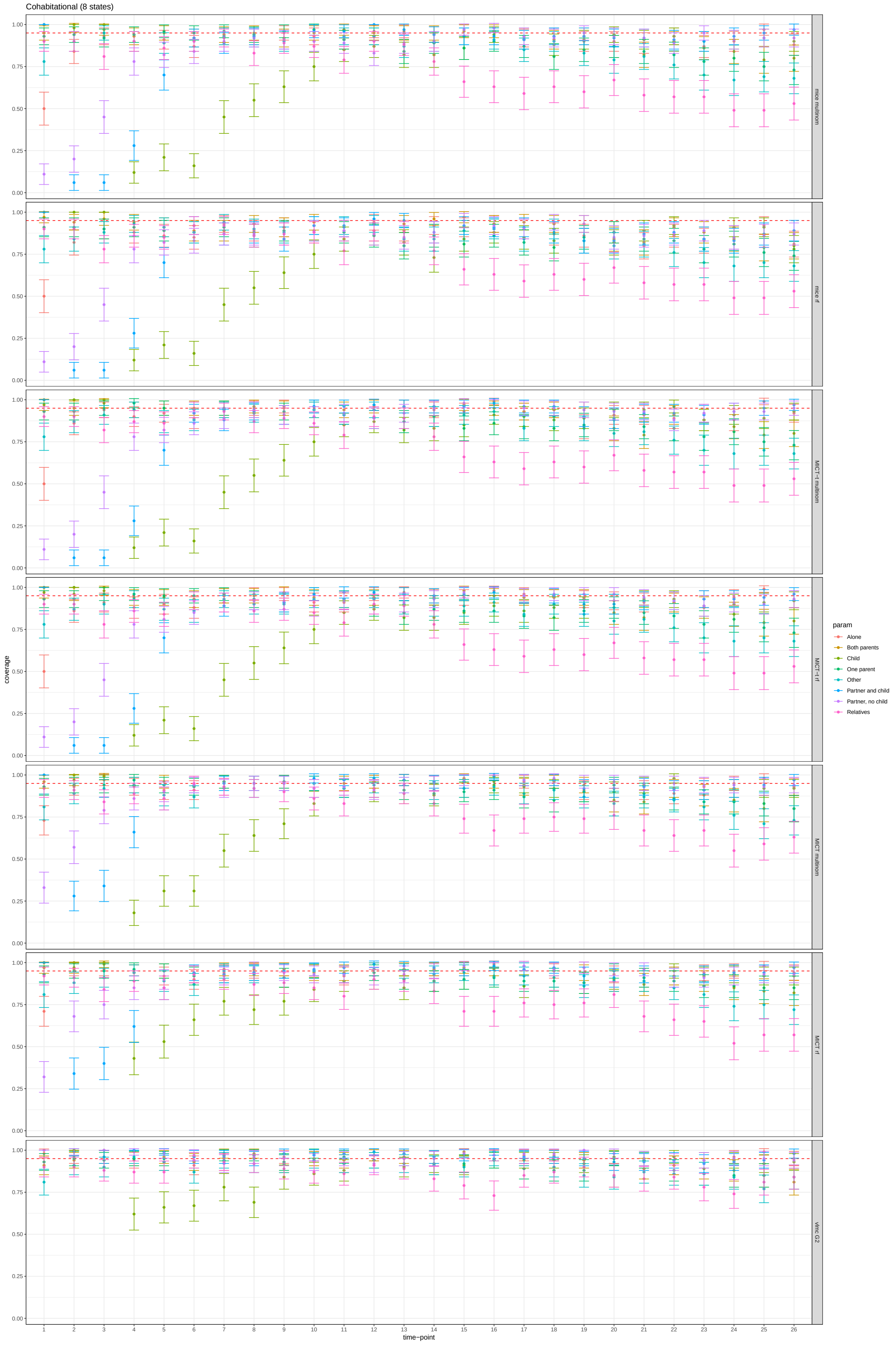

Figure A62: Results regarding the coverage of the timing parameters in the case of an small sample missing data process simulated on cohabitational status coded as eight states. Each panel displays the results of an imputation method. The estimated coverage and its 95% Monte Carlo confidence intervals are shown for the probability of belonging to each state at each time point.

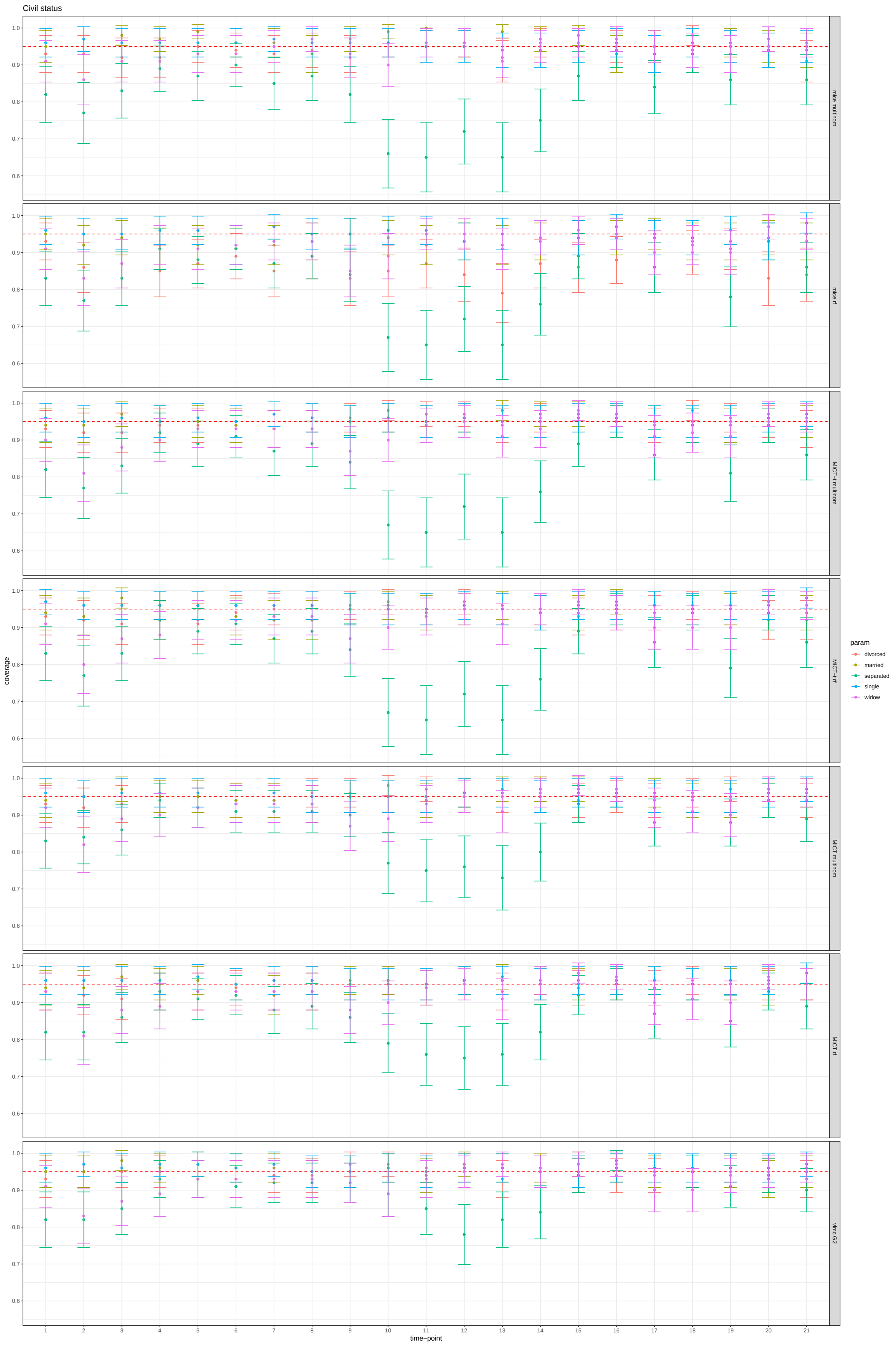

Figure A63: Results regarding the coverage of the timing parameters in the case of an small sample missing data process simulated on civil status. Each panel displays the results of an imputation method. The estimated coverage and its 95% Monte Carlo confidence intervals are shown for the probability of belonging to each state at each time point.

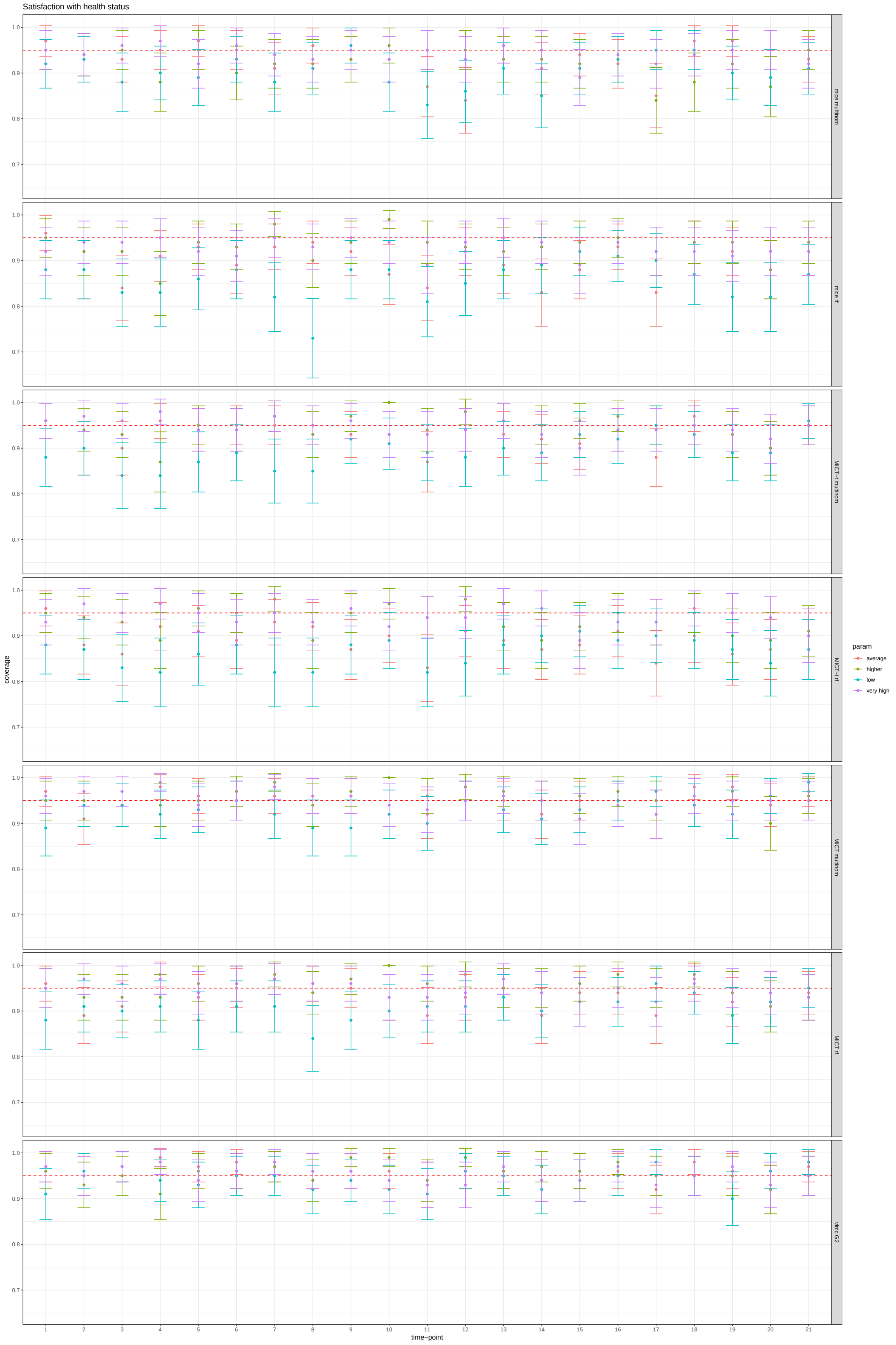

Figure A64: Results regarding the coverage of the timing parameters in the case of an small sample missing data process simulated on satisfaction with health status. Each panel displays the results of an imputation method. The estimated coverage and its 95% Monte Carlo confidence intervals are shown for the probability of belonging to each state at each time point.

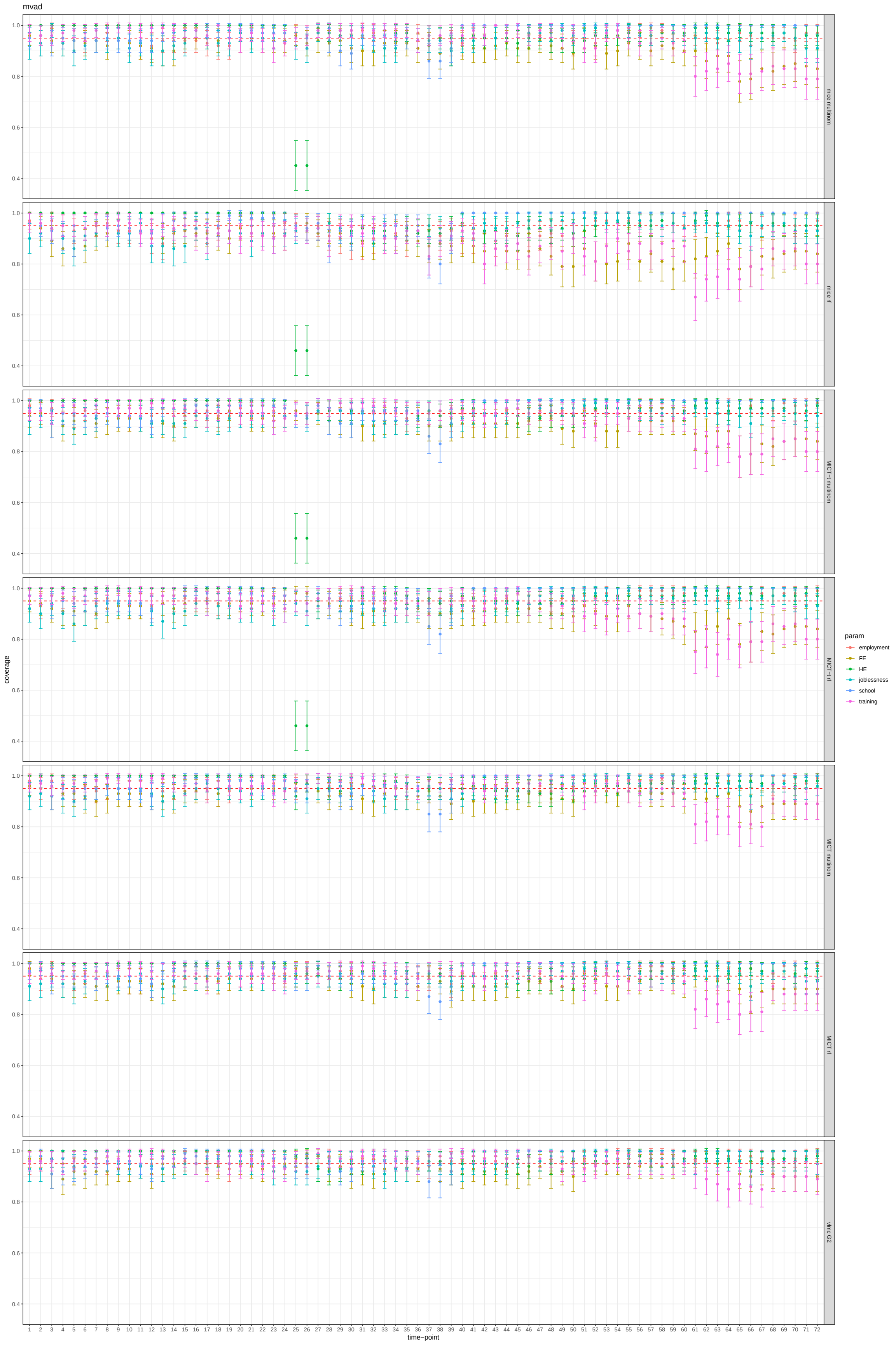

Figure A65: Results regarding the coverage of the timing parameters in the case of an small sample missing data process simulated on school-to-work transitions. Each panel displays the results of an imputation method. The estimated coverage and its 95% Monte Carlo confidence intervals are shown for the probability of belonging to each state at each time point.

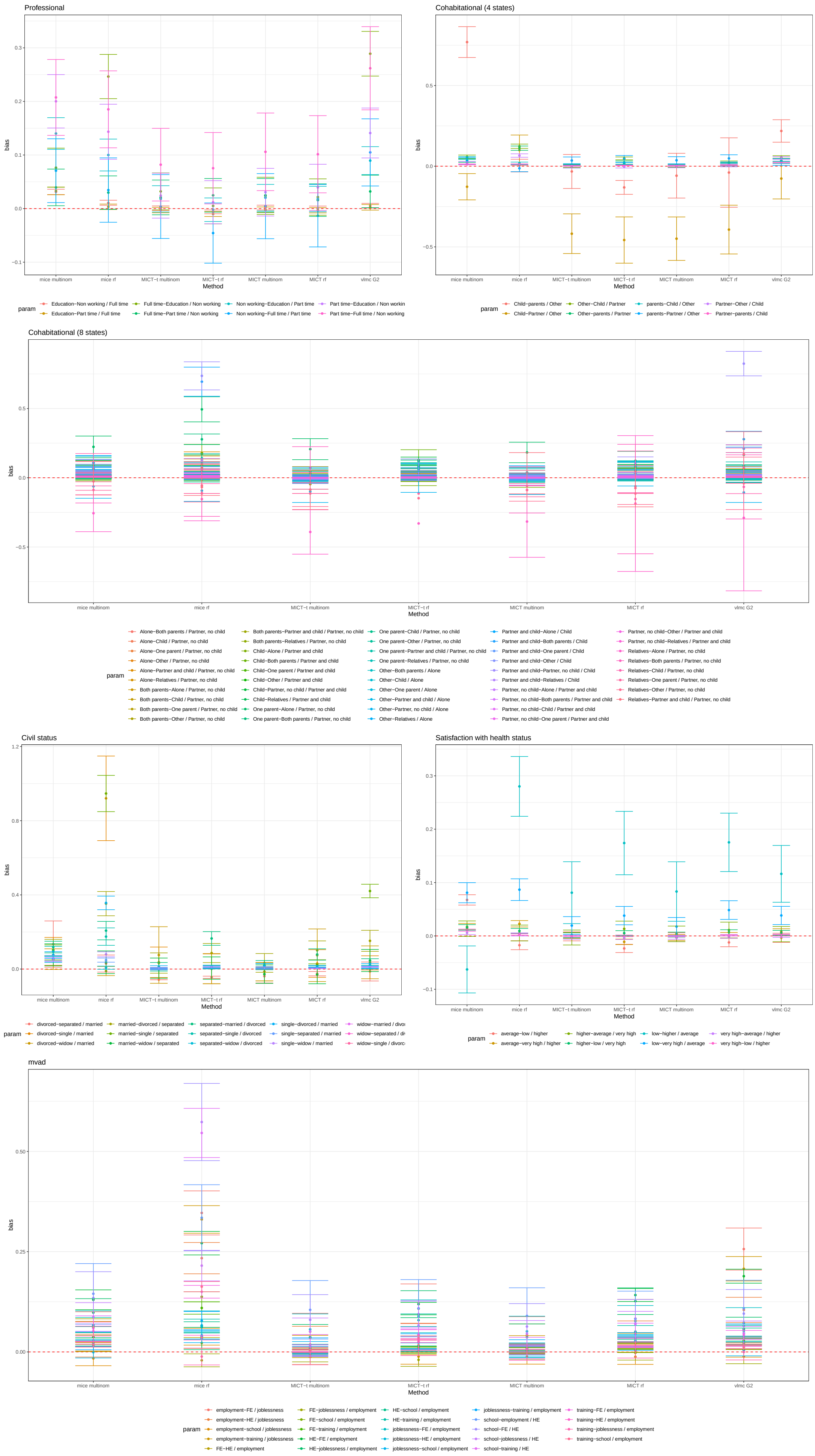

Figure A66: Results regarding the bias of the sequencing parameters in the case of an small sample missing data process. Each panel displays the results of a dataset. The x-axis depicts the different imputation methods. The x-axis depicts the different imputation methods. The estimated bias and its 95% Monte Carlo confidence intervals are shown for each relative risk. They are labeled in the format "state A - state B / state C," representing the relative risk of transitioning to state B versus transitioning to state C when in state A.

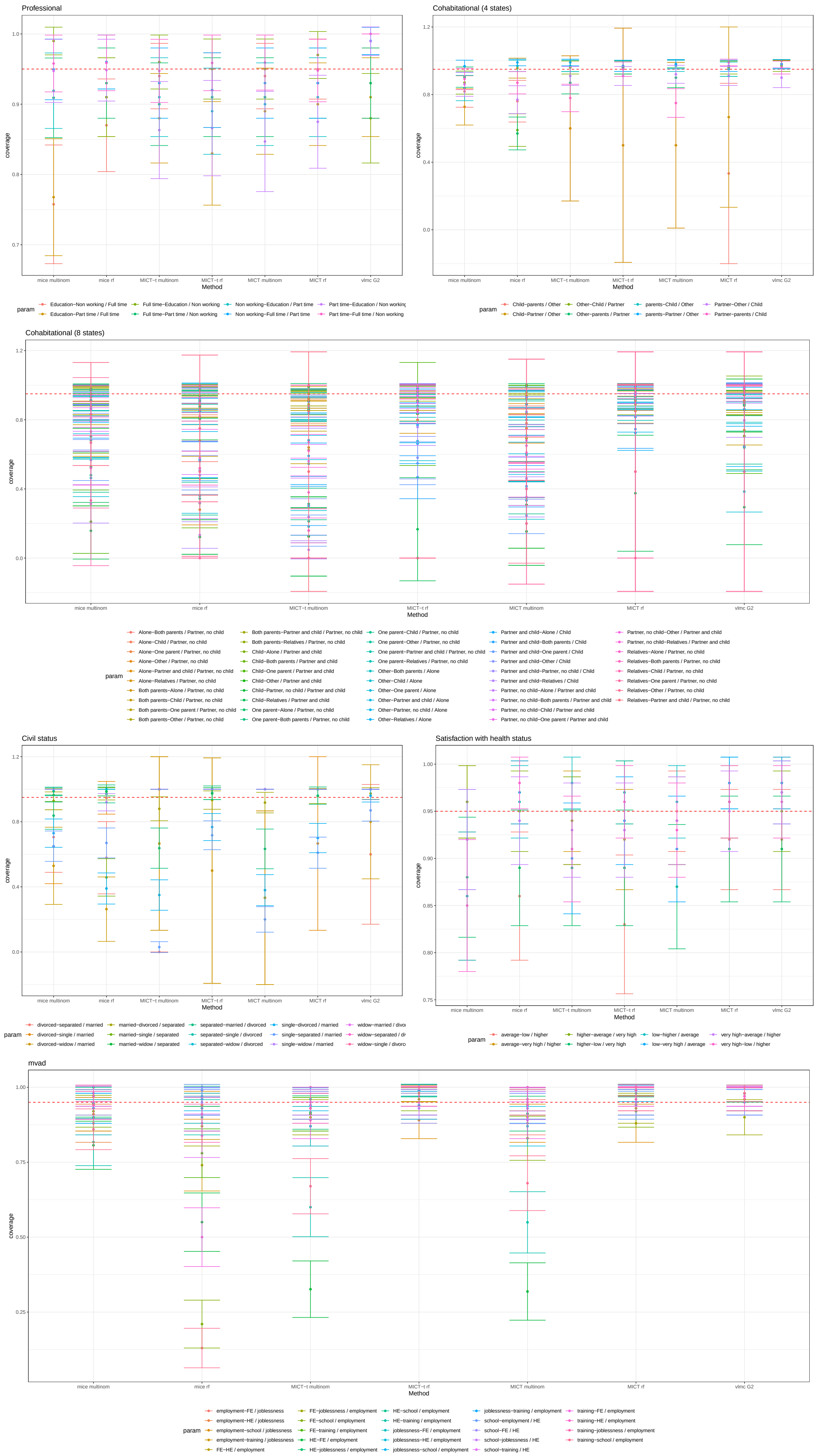

Figure A67: Results regarding the coverage of the sequencing parameters in the case of an small sample missing data process. Each panel displays the results of a dataset. The x-axis depicts the different imputation methods. The x-axis depicts the different imputation methods. The estimated coverage and its 95% Monte Carlo confidence intervals are shown for each relative risk. They are

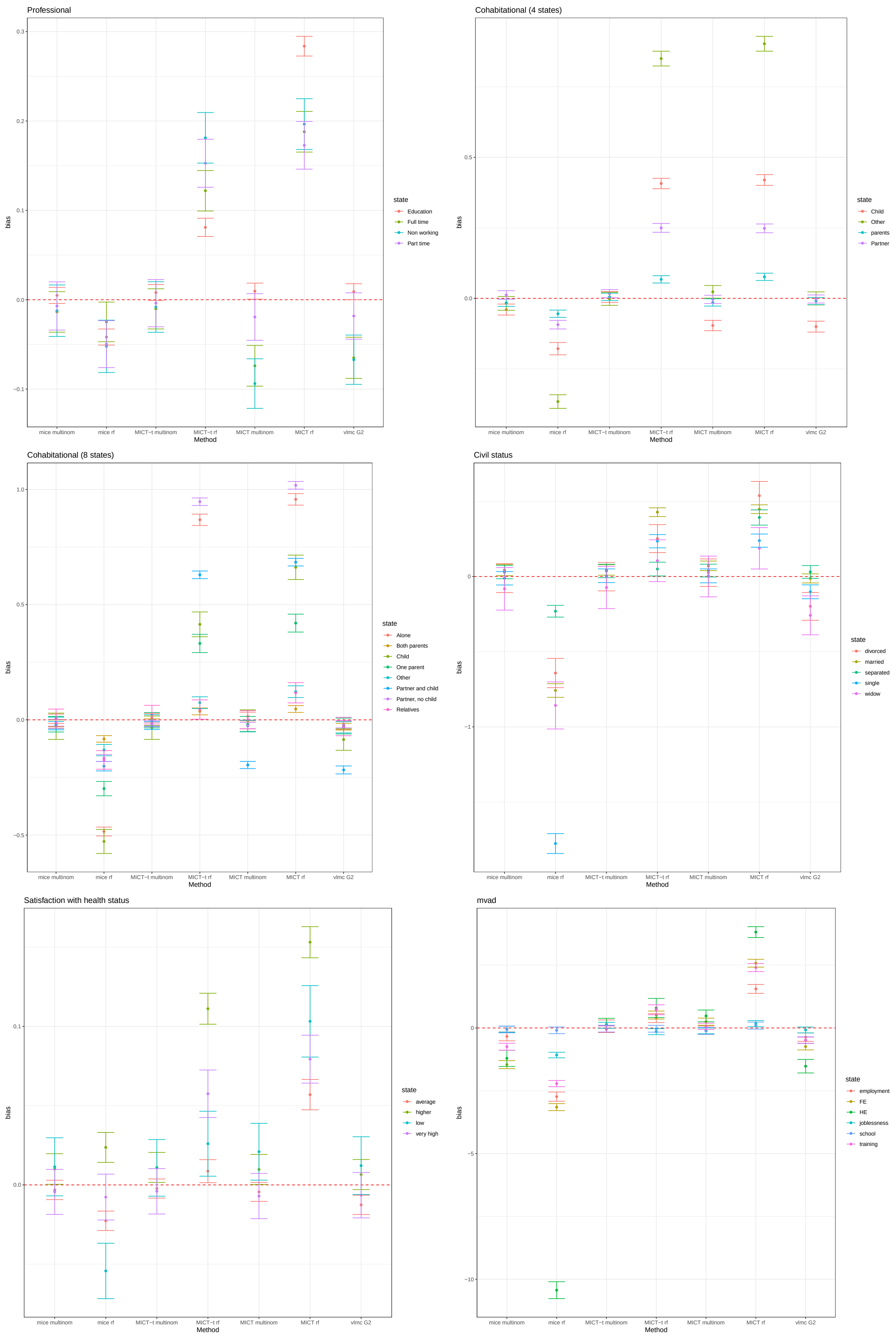

Figure A68: Results regarding the bias of the duration parameters in the case of an attrition process. Each panel displays the results of a dataset. The x-axis depicts the different imputation methods. The estimated bias and its 95% Monte Carlo confidence intervals are provided for the mean time spent in each state.

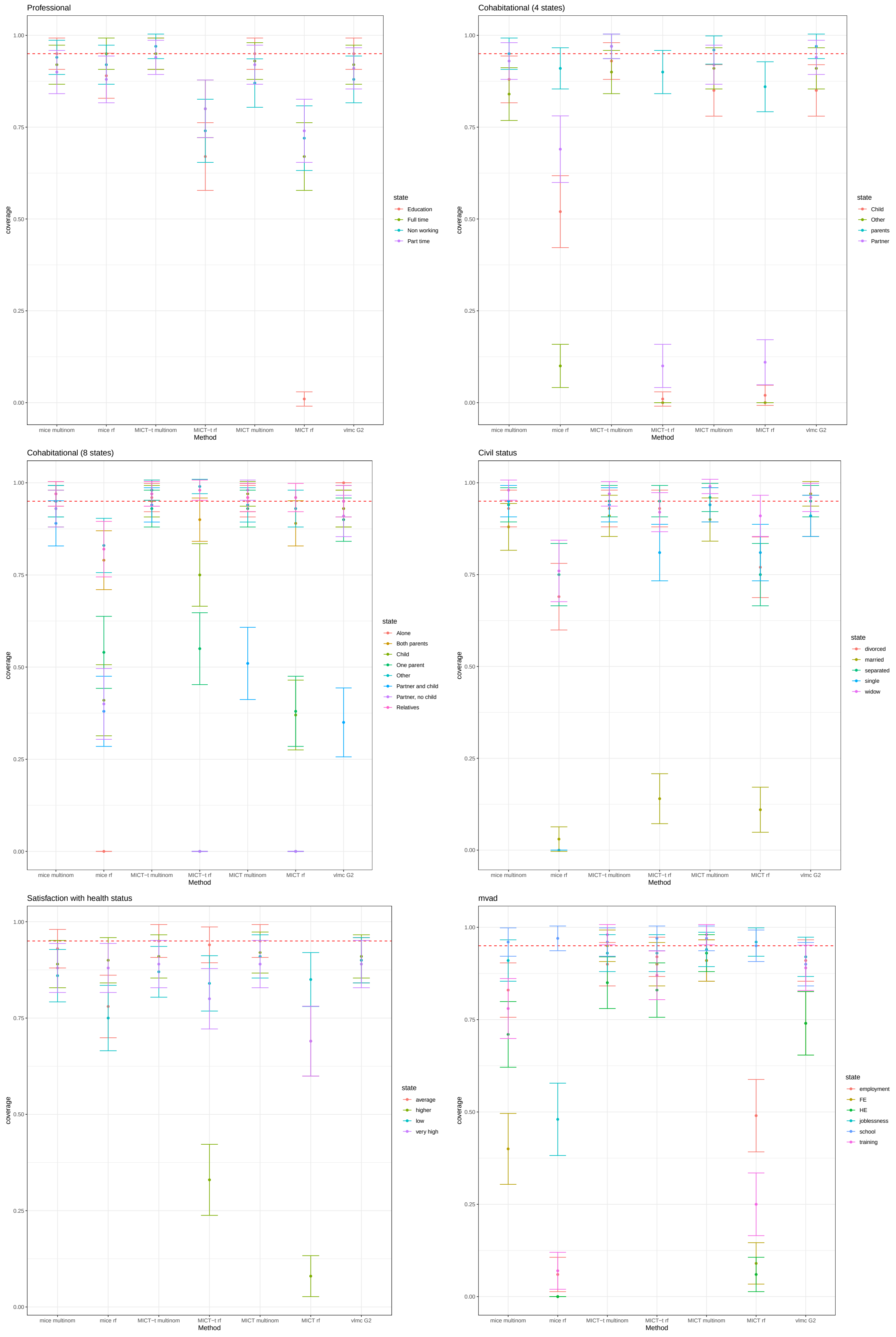

Figure A69: Results regarding the coverage of the duration parameters in the case of an attrition process. Each panel displays the results of a dataset. The x-axis depicts the different imputation methods. The estimated bias and its 95% Monte Carlo confidence intervals are provided for the mean time spent in each state.

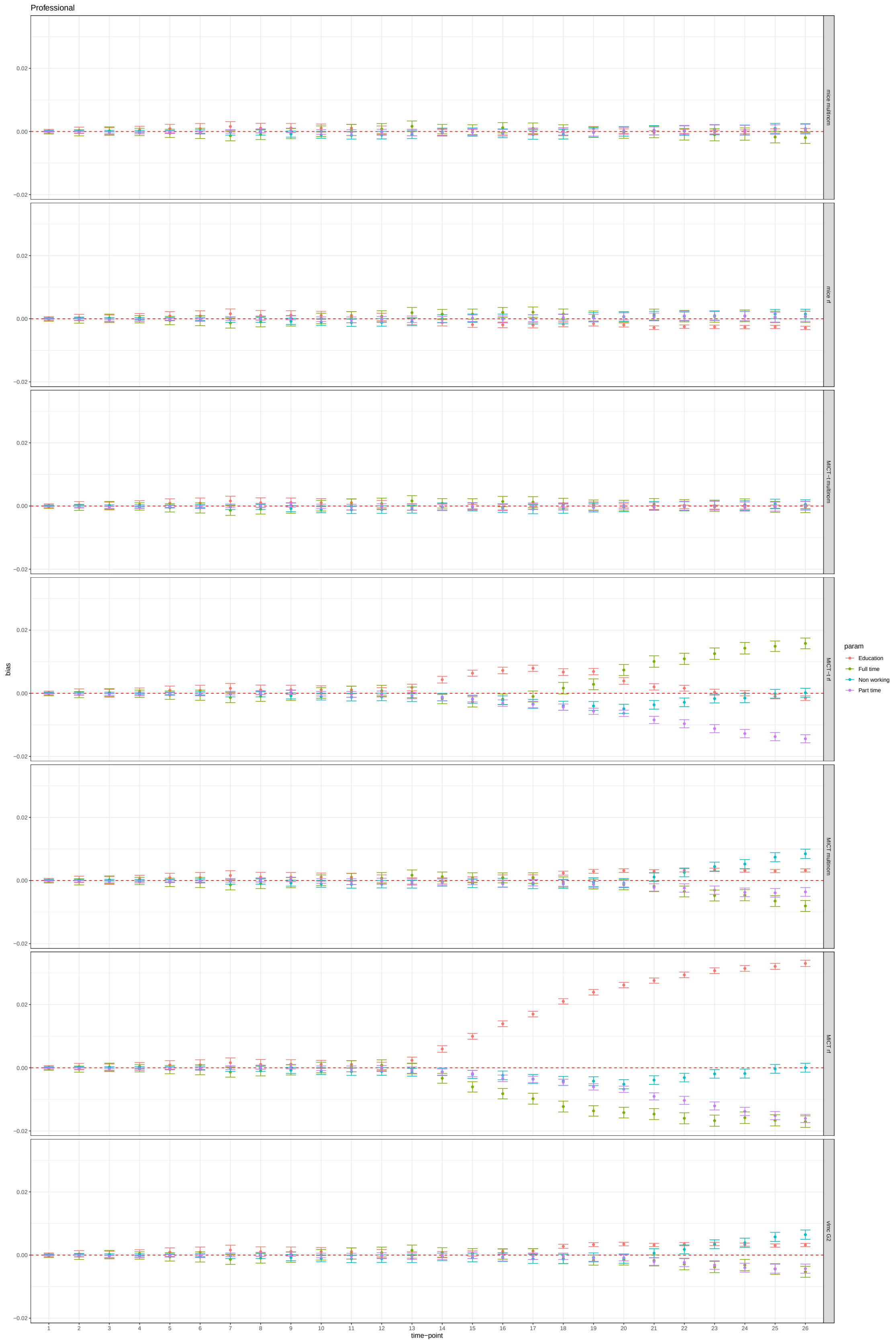

Figure A70: Results regarding the bias of the timing parameters in the case of an attrition process simulated on professional trajectories. Each panel displays the results of an imputation method. The estimated bias and its 95% Monte Carlo confidence intervals are shown for the probability of belonging to each state at each time point.

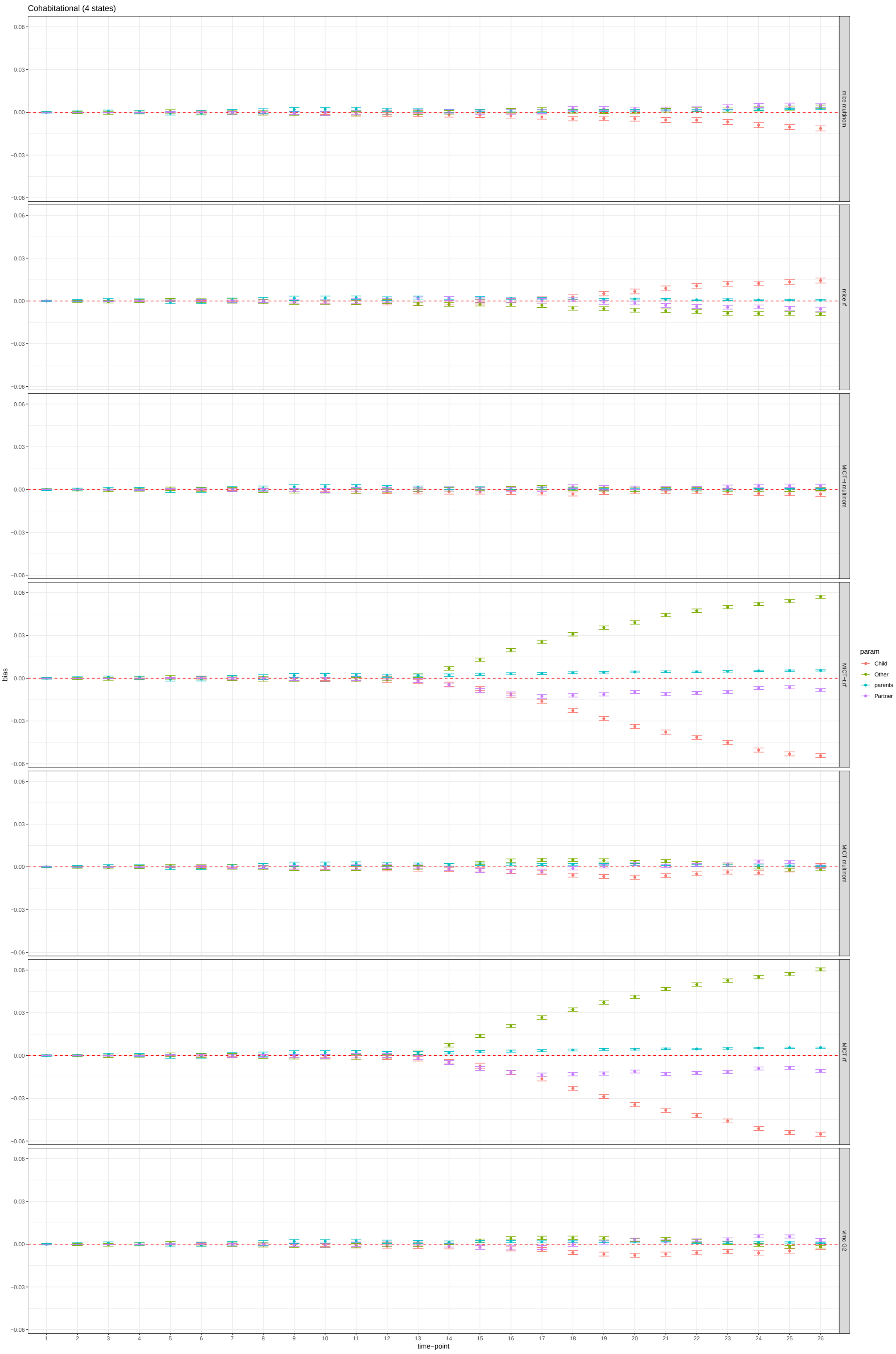

Figure A71: Results regarding the bias of the timing parameters in the case of an attrition process simulated on cohabitational status coded as four states. Each panel displays the results of an imputation method. The estimated bias and its 95% Monte Carlo confidence intervals are shown for the probability of belonging to each state at each time point.

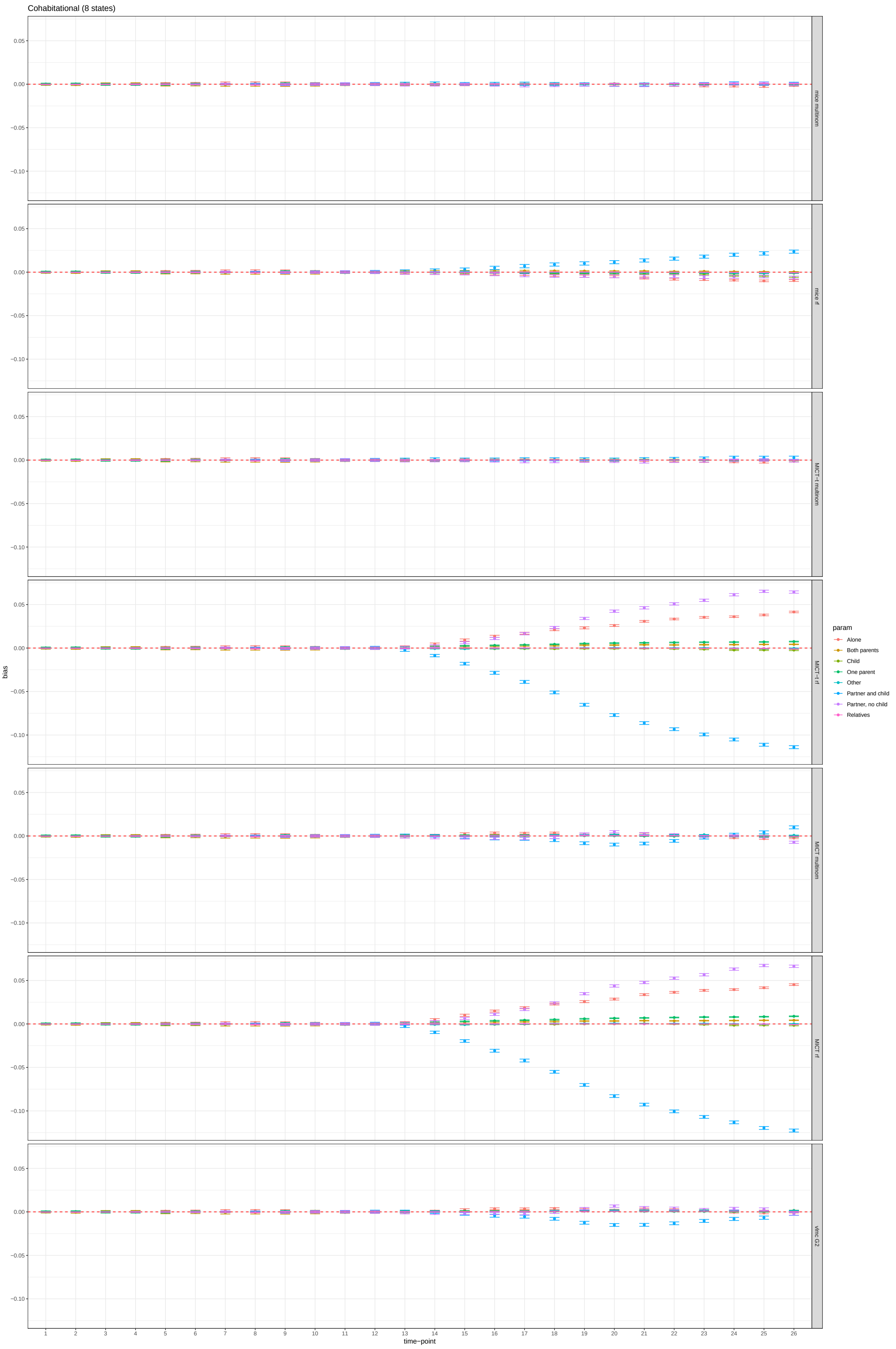

Figure A72: Results regarding the bias of the timing parameters in the case of an attrition process simulated on cohabitational status coded as eight states. Each panel displays the results of an imputation method. The estimated bias and its 95% Monte Carlo confidence intervals are shown for the probability of belonging to each state at each time point.

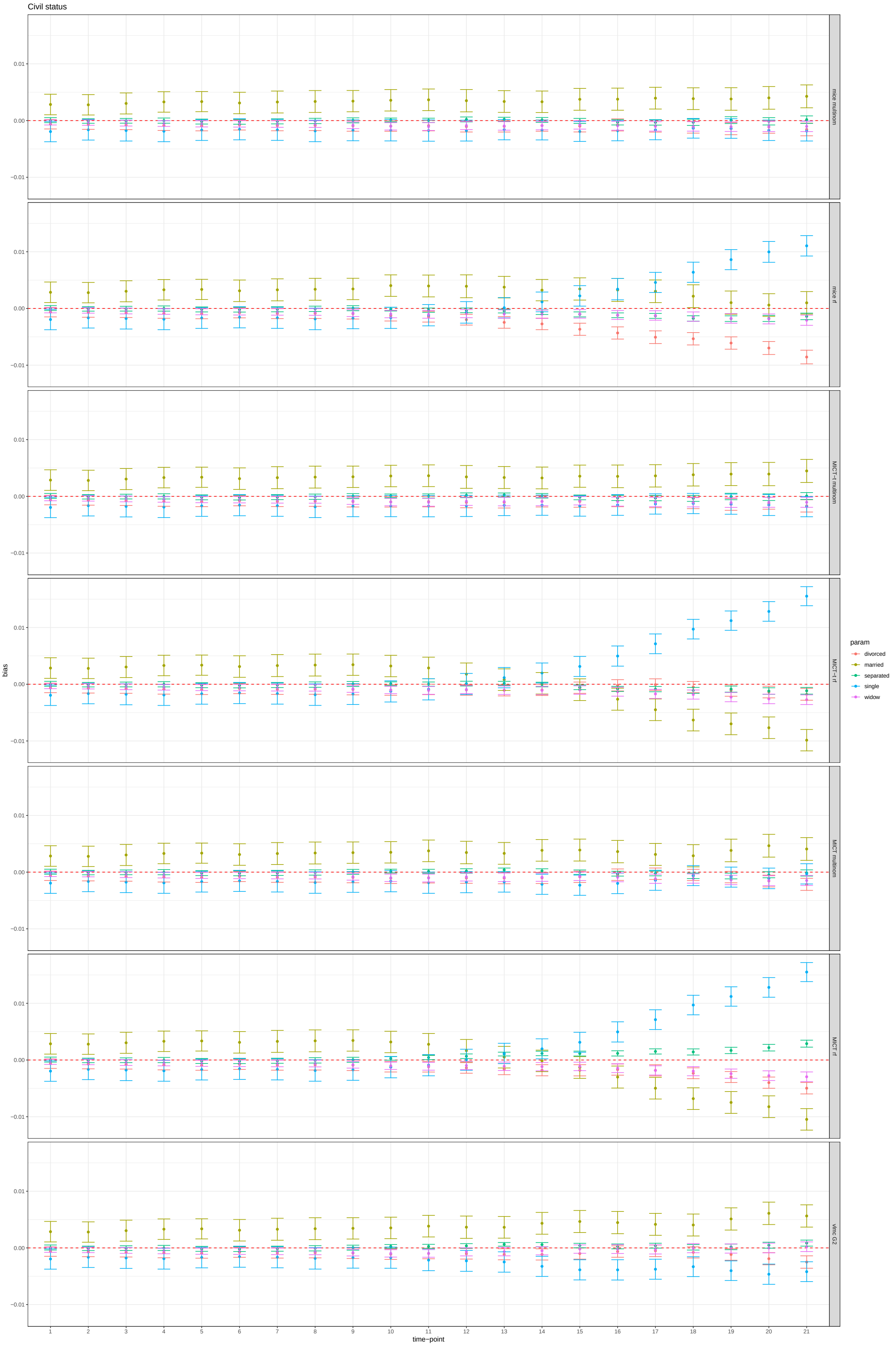

Figure A73: Results regarding the bias of the timing parameters in the case of an attrition process simulated on civil status. Each panel displays the results of an imputation method. The estimated bias and its 95% Monte Carlo confidence intervals are shown for the probability of belonging to each state at each time point.

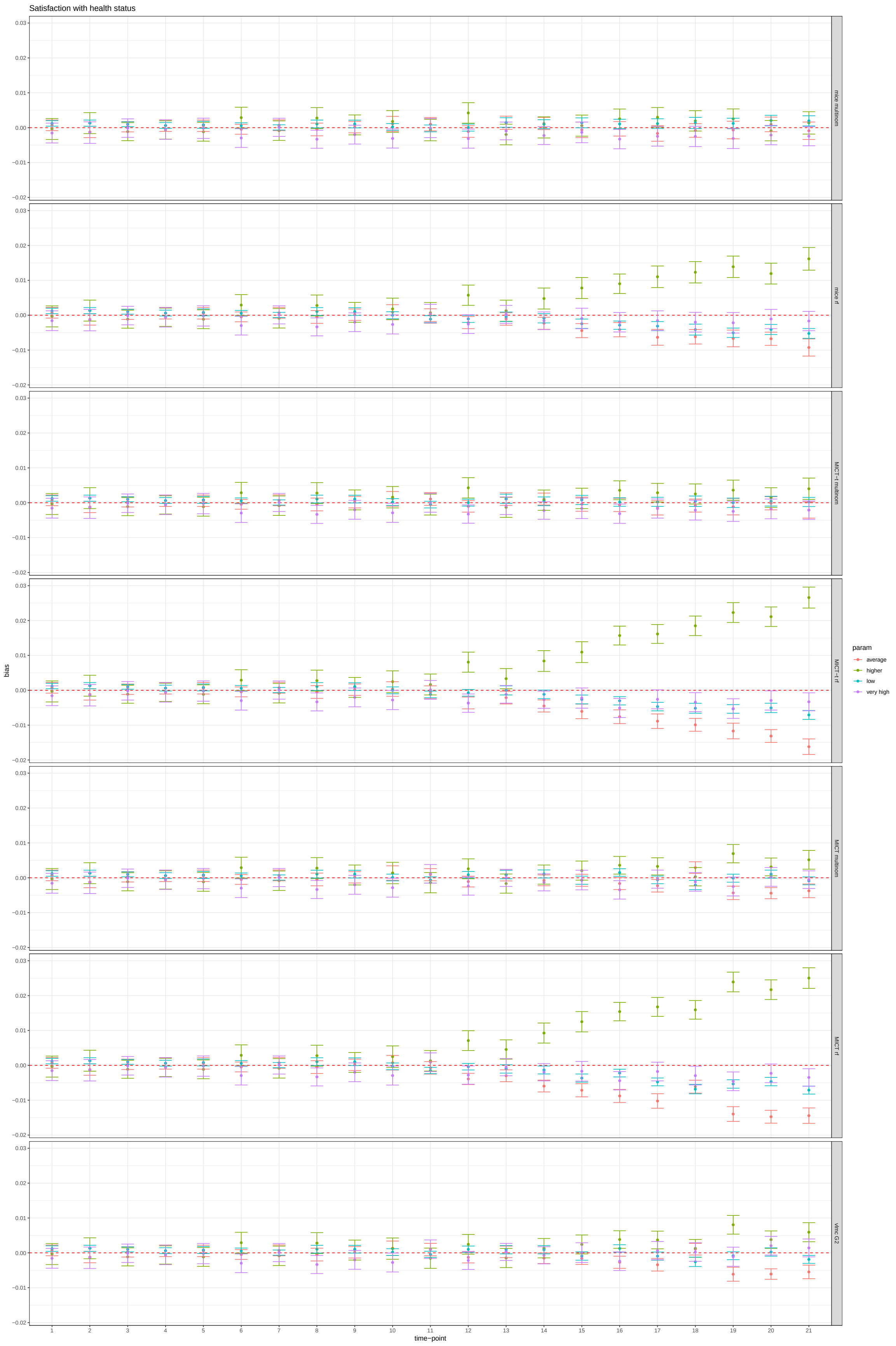

Figure A74: Results regarding the bias of the timing parameters in the case of an attrition process simulated on satisfaction with health status. Each panel displays the results of an imputation method. The estimated bias and its 95% Monte Carlo confidence intervals are shown for the probability of belonging to each state at each time point.

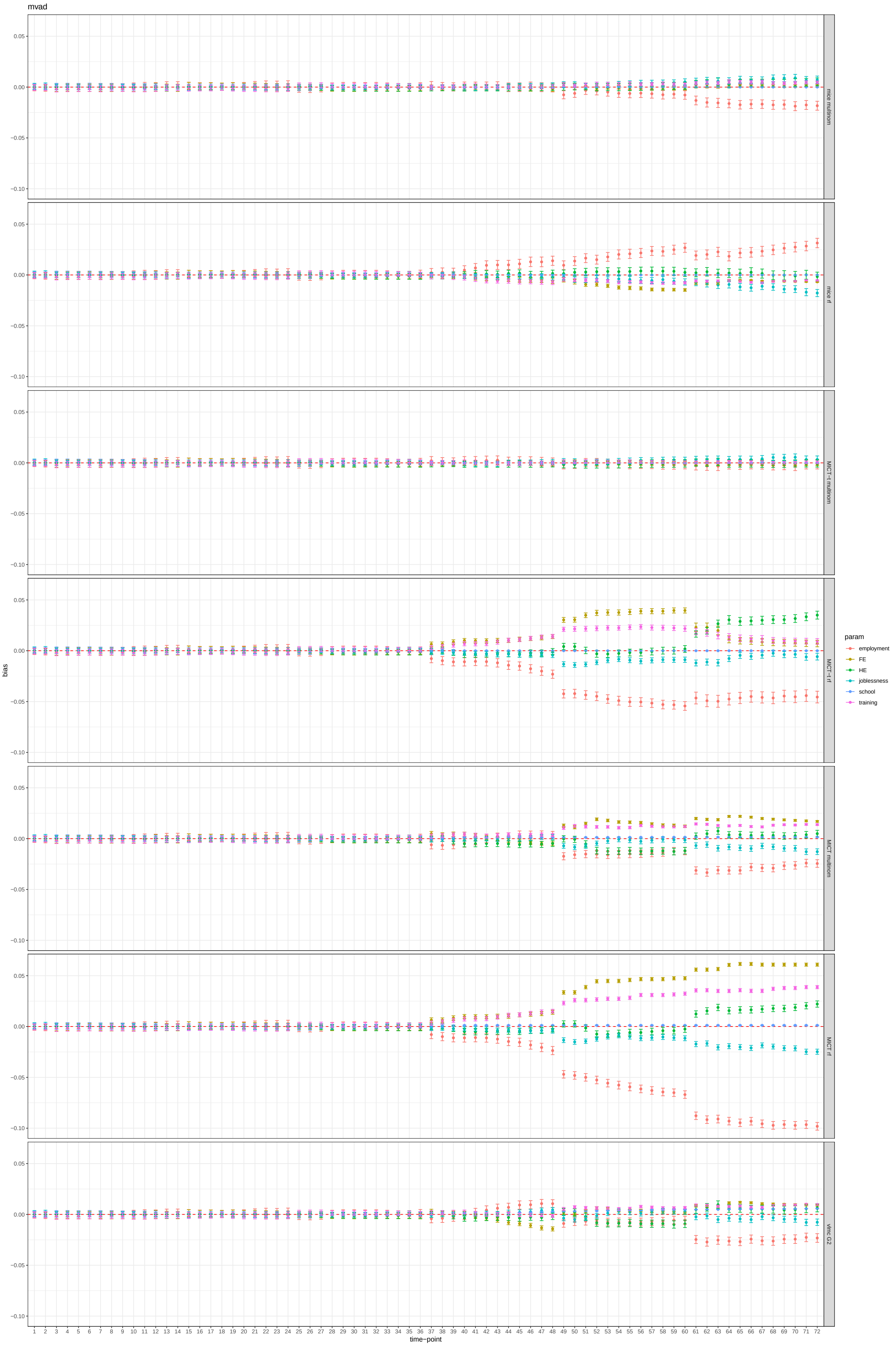

Figure A75: Results regarding the bias of the timing parameters in the case of an attrition process simulated on school-to-work transitions. Each panel displays the results of an imputation method. The estimated bias and its 95% Monte Carlo confidence intervals are shown for the probability of belonging to each state at each time point.

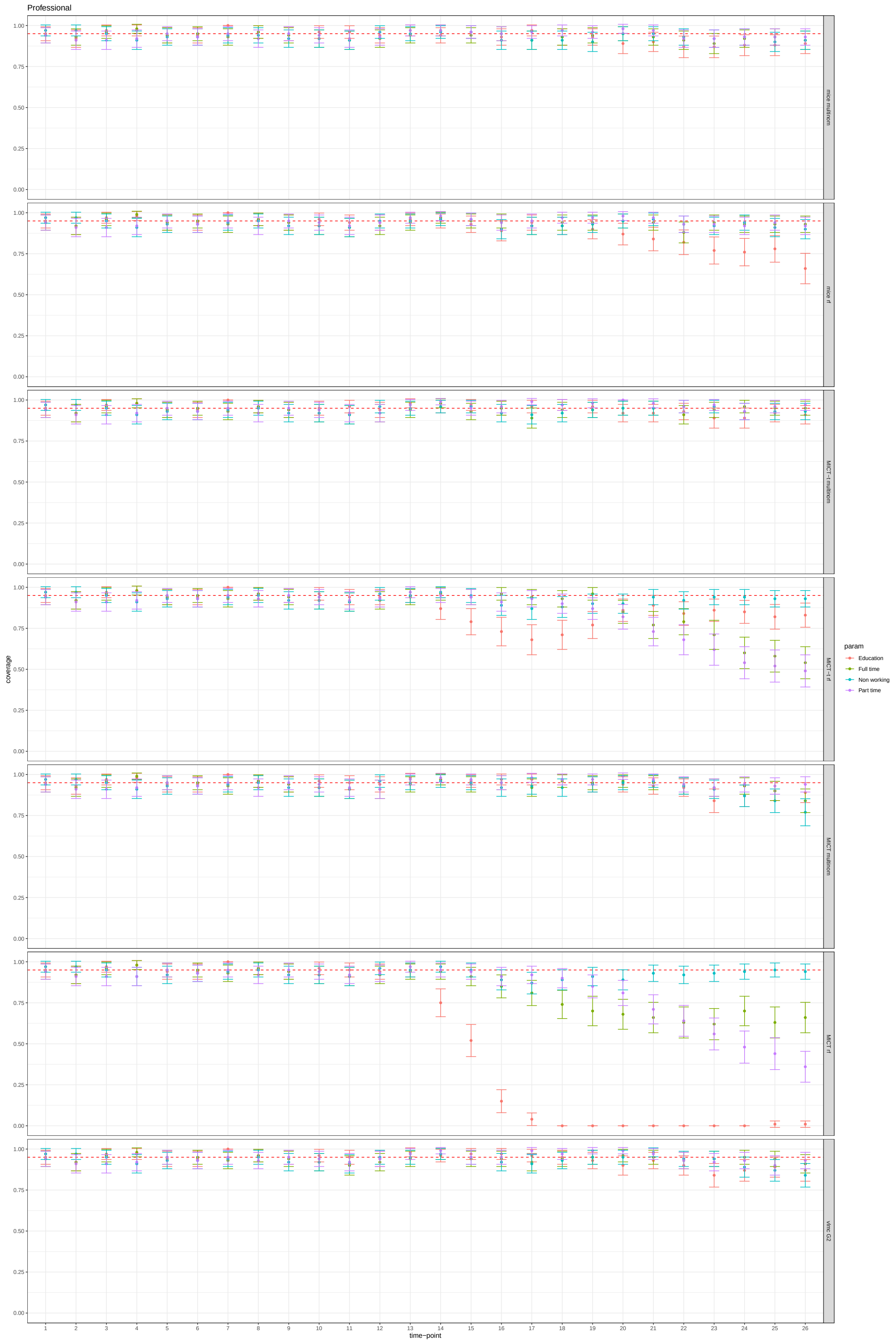

Figure A76: Results regarding the coverage of the timing parameters in the case of an attrition process simulated on professional trajectories. Each panel displays the results of an imputation method. The estimated coverage and its 95% Monte Carlo confidence intervals are shown for the probability of belonging to each state at each time point.

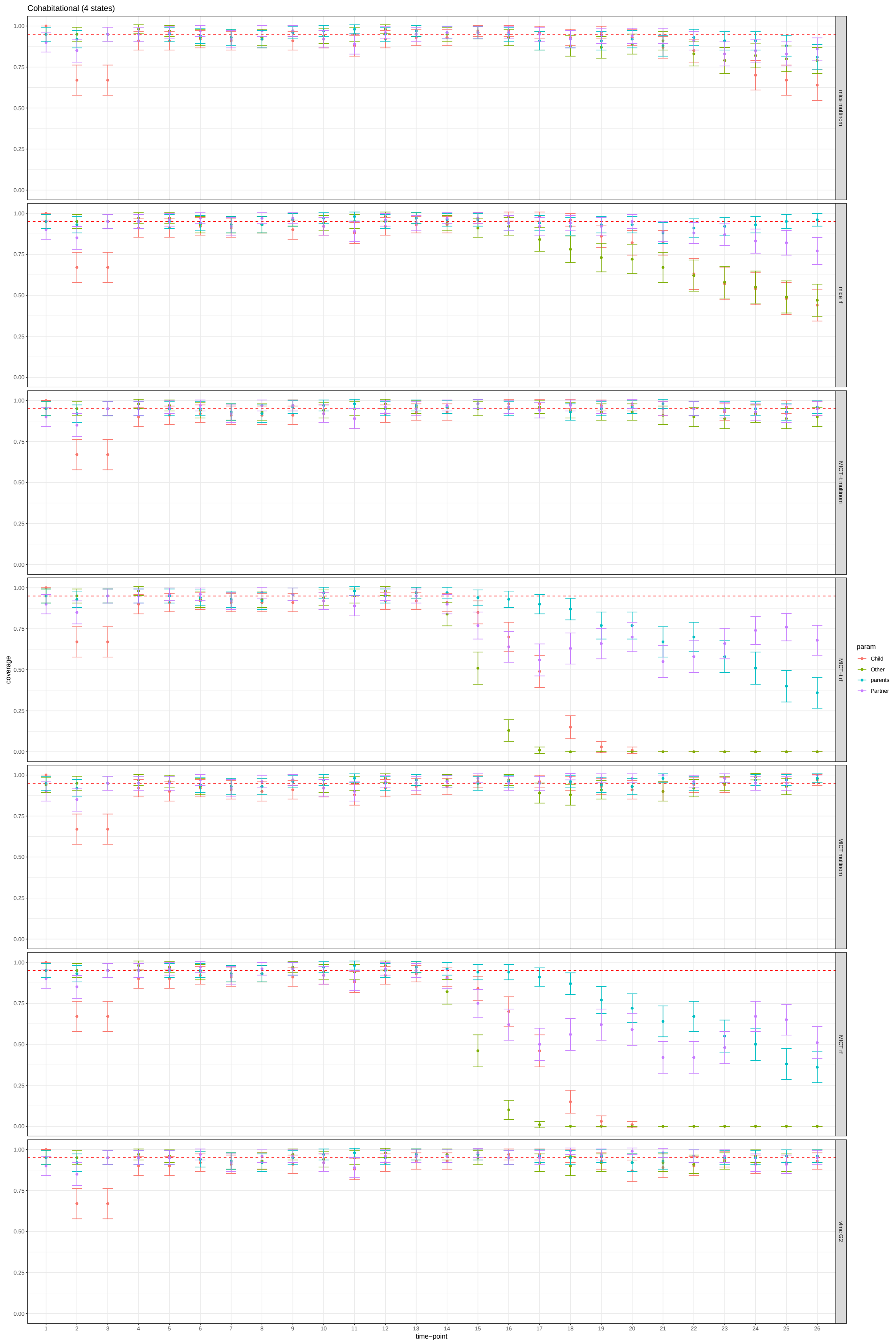

Figure A77: Results regarding the coverage of the timing parameters in the case of an attrition process simulated on cohabitational status coded as four states. Each panel displays the results of an imputation method. The estimated coverage and its 95% Monte Carlo confidence intervals are shown for the probability of belonging to each state at each time point.

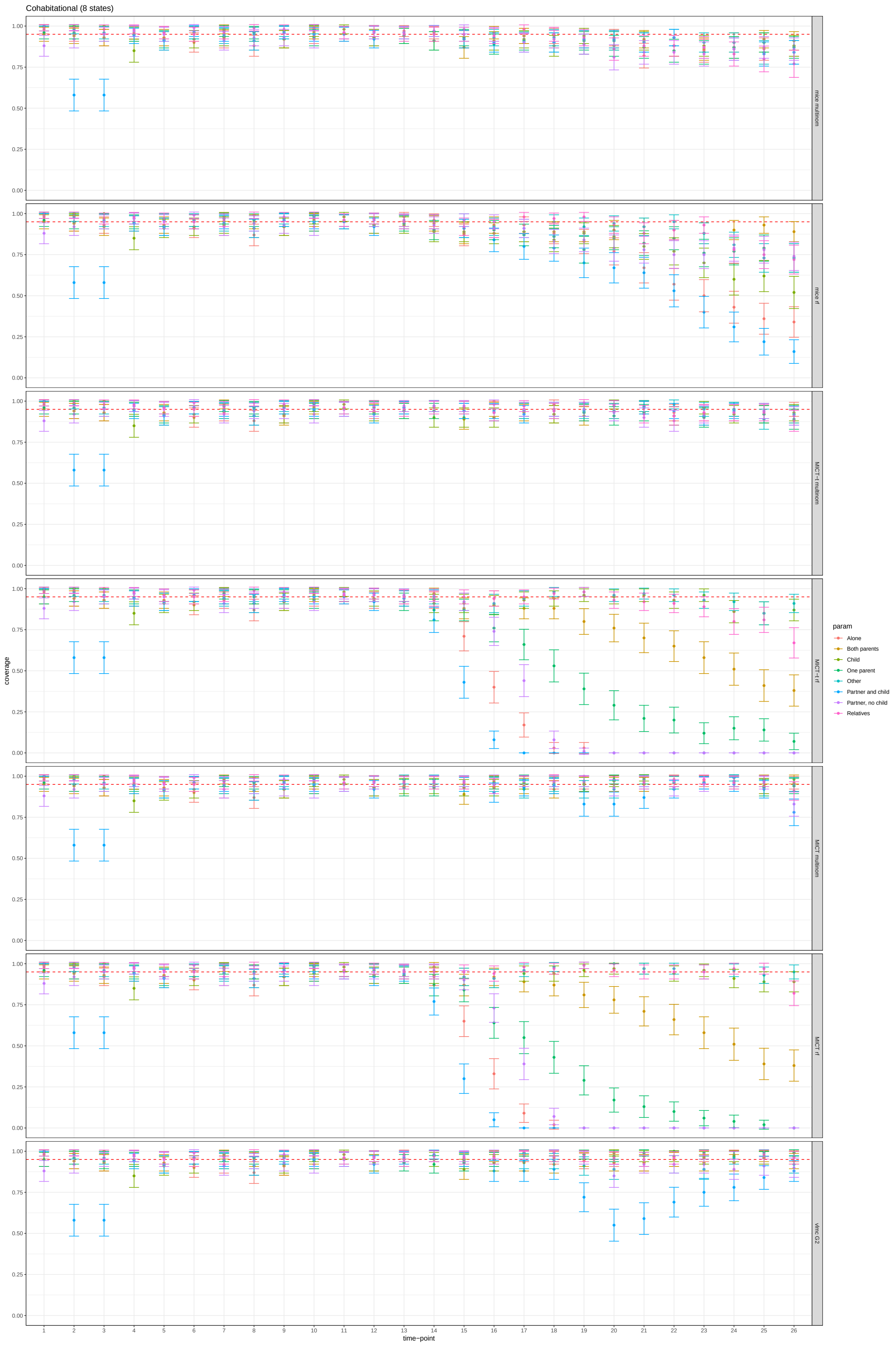

Figure A78: Results regarding the coverage of the timing parameters in the case of an attrition process simulated on cohabitational status coded as eight states. Each panel displays the results of an imputation method. The estimated coverage and its 95% Monte Carlo confidence intervals are shown for the probability of belonging to each state at each time point.
